# Global stomach cancer burden from high-sodium diet and smoking: 1990–2021 findings & 2040 projections

**DOI:** 10.3389/fonc.2026.1838243

**Published:** 2026-06-25

**Authors:** Xiaodie Zhou, Xiaoying Han, Shiliang Dong, Mini Han Wang, Ethan Zhiyuan Lin, Hoiman Ng, Chonin Cheang

**Affiliations:** 1 Gastrointestinal Disease Diagnosis and Treatment Center, Xiangyang Central Hospital, Affiliated Hospital of Hubei University of Arts and Science, Xiangyang, China; 2 Department of Gastrointestinal and Bariatric Surgery, The First Affiliated Hospital of Jinan University, Guangzhou, China; 3 Frontier Computing Center, Zhuhai Institute of Advanced Technology Chinese Academy of Sciences, Zhuhai, China; 4 The Faculty of Medicine, Chinese University of Hong Kong, Hong Kong, Hong Kong SAR, China; 5 OmniContext AI Lab, Hong Kong, Hong Kong SAR, China; 6 Clinical Laboratory, Kiang Wu Hospital, Macao, Macao SAR, China; 7 Macau Yinkui Hospital, Macao, Macao SAR, China; 8 Macau Society of Health Economics, Macao, Macao SAR, China

**Keywords:** global burden of disease (GBD), smoking, socio-demographic index (SDI), sodium intake, stomach cancer

## Abstract

**Background:**

Stomach cancer remains a major global health burden, and high sodium intake and smoking are key modifiable risk factors, yet their long-term and future contributions to global mortality and disability remain unclear.

**Objective:**

(1) assess the global burden of stomach cancer linked to high sodium intake and smoking from 1990 to 2021; (2) forecast the burden from 2021 to 2040.

**Methods:**

We used Global Burden of Disease 2021 data to calculate age- and sex-specific death and DALYs rates, percentage changes, and population attributable fractions for high sodium intake and smoking.

**Results:**

In 2021, 7.9% and 11.2% of global age-standardized stomach cancer deaths were attributed to high sodium intake and smoking, respectively, with men and older adults most affected. East Asia had the highest death and DALY rates, and High-income North America and Western Sub-Saharan Africa the lowest; Mongolia and Bolivia topped sodium-related rates, while Morocco and Nigeria were lowest. From 1990 to 2021, sodium-related mortality rose most in Egypt and fell most in South Korea; smoking-related deaths rose in Egypt and Lesotho but fell in Singapore. High-middle SDI regions bore the greatest burden, and projections to 2040 predict global declines in death and DALY rates for both risks.

**Conclusions:**

Our findings highlight the need for targeted, region- and gender-specific policies to curb stomach cancer risk from high sodium intake and smoking, providing policymakers with vital data for effective public health interventions.

## 1 Background

Stomach cancer, also known as gastric cancer, is the fifth most common type of cancer and the third-leading cause of cancer-related death globally (1), contributing significantly to cancer-related morbidity and mortality worldwide. The disease often progresses silently and is commonly diagnosed at advanced stages, leading to limited treatment options and poor prognosis in many cases. In addition to its health impact, stomach cancer imposes a severe financial burden on affected individuals and their families. A study conducted in China reported that the one-year out-of-pocket expenditure for a newly diagnosed stomach cancer patient was approximately $5,368, representing 63.8% of their household income from the previous year. Consequently, 79.2% of these families faced an unmanageable financial strain due to treatment costs, underscoring the economic challenges associated with this disease (2). The global burden of stomach cancer remains substantial. In 2019, stomach cancer resulted in approximately 1.3 million new cases, 950,000 deaths, and 22.2 million disability-adjusted life years (DALYs) (3), highlighting the urgent need for effective prevention and intervention strategies.

Dietary and lifestyle factors, including high-salt or preserved foods, smoking, alcohol use, and obesity, are recognized contributors to stomach cancer risk (4). High sodium intake, often associated with the consumption of processed and preserved foods, may contribute to stomach cancer by damaging the gastric mucosa, promoting inflammation, and increasing susceptibility to carcinogenic processes (5). Tobacco smoking further compounds the risk of stomach cancer, increasing the likelihood by 1.5 to 1.6 times compared to non-smokers (6). The tissue damage caused by tobacco smoke has been associated with epithelial-mesenchymal transition (EMT), a process linked to the initiation of gastric cancer and progression of carcinogenesis (7).

Although previous studies have examined stomach cancer burden, the combined contribution of high sodium intake and smoking across regions, sexes, age groups, and SDI levels remains insufficiently described using the latest GBD 2021 data. Therefore, this study used GBD 2021 estimates to assess stomach cancer deaths and DALYs attributable to high sodium intake and smoking from 1990 to 2021 at global, regional, and national levels. We also examined patterns by sex, age, and SDI, and projected the future burden to 2040. These findings may support targeted dietary, tobacco-control, and screening strategies across different socioeconomic settings.

## 2 Methods

### 2.1 Overview

The Global Burden of Disease (GBD) 2021 study provides a thorough evaluation of health loss associated with 369 diseases, injuries, and impairments, alongside 88 risk factors, across 204 countries and territories. By utilizing the latest epidemiological data and advanced standardized methodologies, the GBD study offers a detailed assessment spanning from January 1, 1990, to December 31, 2021 (8). The GBD database applies sophisticated techniques to manage missing data and account for confounding factors, ensuring accurate and comprehensive estimates. The design and methodological approach of GBD studies are well-documented in existing literature (8), providing a reliable foundation for understanding global health trends and risk factors. The sample size varies based on the availability of data from different sources. Data sources for the disease burden of stomach cancer were assessed from the Global Health website (http://ghdx.healthdata.org/gbd-results-tool).

Disability-adjusted life years (DALYs) are currently the most widely used and representative metric for assessing overall disease burden. DALYs are calculated by summing the years of life lost (YLLs) due to premature mortality and the years lived with disability (YLDs), providing a holistic view of both fatal and non-fatal health outcomes. In GBD 2021, the numbers of deaths and DALYs are expressed in absolute counts, with rates standardized per 100,000 population based on the updated global age structure (9). The GBD 2021 study estimated the prevalence of risk factor exposure and associated deaths, YLLs, YLDs, and DALYs across 23 age groups, ranging from early neonatal (0 to 6 days) to individuals aged 95 years and older. This analysis included data for both sexes individually and combined, covering 204 countries and territories, which were further organized into 21 regions and seven super-regions (9).

The GBD study aggregates data from diverse sources, including censuses, surveys, health records, and environmental monitors, identified through systematic reviews, official government and organizational websites, and input from a network of collaborators. For most health conditions, this data undergoes analysis using three primary models: the Cause of Death Ensemble model (CODEm), spatiotemporal Gaussian process regression (ST-GPR), and DisMod-MR. These models produce estimates stratified by age, sex, location, and year, ensuring a standardized and comprehensive approach to analyzing global health data (10).

The GBD data is accessible at https://www.healthdata.org/data-tools-practices/interactive-visuals/gbd-results. The detailed study validity protocol is available on the Institute of Health Measurement and Evaluation’s website (https://www.healthdata.org/gbd/about/protocol).

### 2.2 Definitions

According to the GBD definition, a diet high in sodium is characterized by an average 24-hour urinary sodium excretion exceeding 3 grams per day (11). Smoking is defined as the current daily or occasional use of any smoked tobacco product (12). In GBD 2021, diseases and injuries were organized into a four-level hierarchy. Level 1 consists of three main groups: communicable, maternal, neonatal, and nutritional diseases; non-communicable diseases; and injuries. Level 2 includes 22 specific disease and injury categories within these Level 1 groups, such as cardiovascular disease within the non-communicable diseases category. Level 3 further refines these categories into more specific causes, like stroke within the cardiovascular disease group. Level 4 identifies sub-causes of certain Level 3 conditions, such as ischemic stroke within the stroke category (13). Our study will primarily focus on Level 3 diseases.

To assess the disease burden in relation to social development, the findings were analyzed using the Socio-demographic Index (SDI). The SDI is a composite measure incorporating income per capita, years of schooling, and fertility rate among females under 25 years of age. It ranges from 0 (indicating low income, low education, and high fertility) to 1 (indicating high income, high education, and low fertility). Based on 2021 SDI values, we categorized the 204 countries and territories into five groups: low-SDI, low-middle-SDI, middle-SDI, high-middle-SDI, and high-SDI quintiles (14).

### 2.3 Statistical analysis

We estimated the disease burden attributable to diet high in sodium and smoking by calculating the number of deaths, DALYs, age-standardized rate (per 100,000 population), percentage change, and population attributable fraction (PAF), each with 95% uncertainty intervals (UIs). This analysis covered various categories, including age, sex, year, and location. UIs were derived from 1,000 draw-level estimates for each parameter, with 95% UIs defined by the 25th and 975th ranked values of these estimates. The age-standardized rate was calculated by adjusting the age distribution based on the GBD 2021 standard population. The PAF, obtained from GBD, represents the proportion of health outcomes, such as deaths and DALYs, in a population attributable to diet high in sodium and smoking. It indicates the percentage of these health outcomes that could be avoided if diet high in sodium and smoking, significant risk factors, were eliminated. A higher PAF suggests a greater number of deaths and DALYs attributed to diet high in sodium and smoking. Additionally, we analyzed the relationship between the disease burden attributable to diet high in sodium and smoking and the SDI, factoring in location and year. The codes used for statistical, analytical, processing, and estimation steps are available at https://ghdx.healthdata.org/gbd-2021/code. The supplementary data have been deposited in a publicly available database and are accessible at the following link: https://doi.org/10.5281/zenodo.17881307.

### 2.4 Projection model

To project the global stomach cancer burden attributable to high sodium intake and smoking from 2021 to 2040, we used a Bayesian age–period–cohort projection model implemented in RStudio 4.4. 1 (15). The model used historical age-specific GBD 2021 estimates from 1990 to 2021 as inputs and extrapolated past age, period, and cohort patterns to estimate future age-standardized death and DALY rates. The projection assumes that historical trends continue during the projection period. It does not incorporate future changes in sodium consumption, smoking prevalence, population structure, healthcare access, screening coverage, or policy interventions. Therefore, the projected estimates should be interpreted as descriptive trend-based projections rather than scenario-based forecasts.

## 3 Results

### 3.1 Age-specific trends in mortality and DALYs attributable to diet high in sodium and smoking in 2021

In 2021, approximately 7.928% (95% UI 0.000, 40.203) and 11.181% (95% UI 9.283,13.158) of all age-standardized deaths worldwide were attributable to diet high in sodium and smoking respectively. Approximately 7.924% (95% UI 0.000, 40.127) and 11.020% (95% UI 9.138-12.851) of all age-standardized deaths and DALYs worldwide were attributable to diet high in sodium and smoking respectively (**Tables 1**, **2**).

**Table 1:** Age-standardized deaths and DALYs attributable to diet high in sodium in 2021 and percentage change from 1990 to 2021, by gender, SDI quintile and 21GBD regions.

	Deaths				DALYs			
	2021 age-standardized rate per 100,000 people	Percentage change in age-standardized rate, 1990–2021	2021 age-standardized PAF	Percentage change in agestandardized PAF, 1990–2021	2021 age-standardized rate per 100,000 people	Percentage change in age-standardized rate, 1990–2021	2021 age-standardized PAF	Percentage change in agestandardized PAF, 1990–2021
Global	0.887(-0.000-4.370)	-0.491(-0.751--0.415)	7.928(-0.000-40.203)	0.290(-50.001-3.769)	20.783(-0.000-102.378)	-0.533(-0.828--0.462)	7.924(-0.000-40.127)	-0.175(-62.214-1.725)
Sex								
Male	1.292(-0.000-6.341)	-0.474(-0.864--0.355)	8.071(-0.000-40.643)	0.268(-73.552-5.552)	29.900(-0.000-146.648)	-0.519(-0.828--0.414)	8.074(-0.000-40.598)	0.031(-63.540-4.063)
Female	0.547(0.000-2.795)	-0.526(-0.871--0.458)	7.653(0.000-39.420)	-0.126(-73.433-3.094)	12.607(0.000-64.624)	-0.561(-0.791--0.495)	7.615(0.000-38.910)	-0.927(-50.090-1.003)
Socio-demographic index								
low	0.599(0.000-3.183)	-0.302(-0.734--0.215)	7.119(0.000-37.558)	-1.232(-63.794-0.448)	14.711(0.000-78.407)	-0.345(-0.755--0.256)	7.058(0.000-37.251)	-2.039(-65.846--0.189)
Low-middle	0.592(0.000-3.004)	-0.275(-0.729--0.162)	7.706(0.000-39.671)	-0.938(-63.503-0.668)	14.783(0.000-75.093)	-0.309(-0.791--0.212)	7.713(0.000-39.563)	-0.713(-67.795-1.762)
Middle	1.107(-0.000-5.430)	-0.519(-0.937--0.443)	8.075(-0.000-40.654)	-1.052(-84.560-0.064)	25.818(-0.000-127.576)	-0.563(-0.943--0.488)	8.072(-0.000-40.582)	-0.998(-84.244-0.098)
High-Middle	1.184(-0.000-5.791)	-0.515(-0.697--0.339)	7.935(-0.000-40.127)	1.322(-29.239-21.394)	28.139(-0.000-136.923)	-0.558(-0.789--0.396)	7.977(-0.000-40.236)	0.991(-53.597-17.995)
High	0.543(0.000-2.733)	-0.561(-0.865--0.526)	7.932(0.000-40.418)	1.730(-67.957-7.120)	11.603(0.000-58.092)	-0.612(-0.845--0.586)	7.929(0.000-40.344)	1.163(-59.445-4.222)
Region								
Andean Latin America	1.707(0.000-8.649)	-0.360(-0.784--0.180)	8.039(0.000-40.880)	-0.891(-64.093-2.666)	38.451(0.000-193.858)	-0.384(-0.789--0.214)	7.975(0.000-40.584)	-0.775(-63.969-3.061)
Australasia	0.251(0.000-1.410)	-0.483(-0.954--0.024)	6.418(0.000-35.988)	2.038(-90.941-89.448)	5.716(0.000-31.140)	-0.492(-0.954-0.050)	6.739(0.000-36.858)	2.395(-90.210-108.256)
Caribbean	0.578(0.000-3.099)	-0.384(-0.872--0.275)	7.456(0.000-39.202)	-1.695(-80.039-1.776)	14.237(0.000-76.674)	-0.351(-0.894--0.201)	7.328(0.000-38.625)	-1.211(-85.379-11.737)
Central Asia	0.898(0.000-4.604)	-0.578(-0.908--0.540)	7.774(0.000-39.961)	-4.203(-79.605--1.535)	23.075(0.000-119.442)	-0.602(-0.927--0.567)	7.731(0.000-39.776)	-4.198(-80.994--1.575)
Central Europe	0.712(0.000-3.491)	-0.537(-0.809--0.496)	8.307(0.000-41.409)	0.194(-55.001-2.795)	16.708(0.000-81.881)	-0.545(-0.884--0.505)	8.279(0.000-41.294)	0.317(-73.276-3.156)
Central Latin America	0.933(0.000-4.832)	-0.450(-0.796--0.354)	8.009(0.000-40.569)	-0.199(-63.982-9.634)	22.411(0.000-116.518)	-0.419(-0.854--0.316)	7.966(0.000-40.297)	-0.341(-75.885-7.510)
Central Sub-Saharan Africa	0.513(0.000-2.992)	-0.246(-0.468-3.414)	5.768(0.000-32.969)	2.615(-16.462-522.028)	12.538(0.000-73.888)	-0.261(-0.483-2.130)	5.717(0.000-32.825)	2.920(-25.519-374.875)
East Asia	1.763(-0.000-8.690)	-0.533(-0.883--0.416)	8.299(-0.000-41.394)	-0.167(-68.897-1.080)	41.092(-0.000-206.627)	-0.575(-0.842--0.457)	8.282(-0.000-41.282)	-0.063(-56.725-1.133)
Eastern Europe	0.923(0.000-4.736)	-0.589(-1.065--0.477)	7.574(0.000-39.604)	0.256(-116.867-25.954)	23.544(0.000-119.384)	-0.618(-0.998--0.509)	7.661(0.000-39.810)	0.211(-99.339-29.252)
Eastern Sub-Saharan Africa	0.538(0.000-2.793)	-0.399(-0.841--0.323)	7.587(0.000-38.853)	-5.939(-76.157--3.178)	12.943(0.000-67.732)	-0.446(-0.841--0.353)	7.381(0.000-38.131)	-7.580(-72.813--3.722)
High-income Asia Pacific	1.088(0.000-5.431)	-0.647(-0.889--0.622)	8.261(0.000-41.301)	-0.720(-69.436-0.213)	22.571(0.000-112.133)	-0.698(-0.913--0.672)	8.240(0.000-41.202)	-0.538(-70.900-0.280)
High-income North America	0.227(0.000-1.172)	-0.430(-0.471-3.011)	7.676(0.000-39.824)	7.456(2.061-661.902)	5.522(0.000-28.105)	-0.409(-0.447-3.418)	7.787(0.000-40.076)	7.777(1.912-697.997)
North Africa and Middle East	0.451(0.000-2.751)	-0.400(-0.756-0.395)	4.794(0.000-28.891)	-3.446(-58.303-120.907)	11.002(0.000-65.918)	-0.440(-0.696-0.223)	5.081(0.000-30.219)	-2.159(-48.166-112.150)
Oceania	1.059(0.000-5.520)	-0.221(-0.897-0.031)	7.883(0.000-40.150)	2.475(-84.083-14.811)	25.520(0.000-134.982)	-0.220(-0.896-0.083)	7.500(0.000-38.846)	3.967(-83.291-26.019)
South Asia	0.447(0.000-2.252)	-0.281(-0.401-0.267)	7.846(0.000-39.994)	0.253(-13.771-59.942)	11.449(0.000-57.604)	-0.327(-0.432-0.221)	7.913(0.000-40.108)	0.927(-7.052-64.178)
Southeast Asia	0.564(0.000-2.889)	-0.378(-0.732--0.215)	8.266(0.000-41.249)	-0.350(-56.457-2.621)	13.981(0.000-71.253)	-0.405(-0.779--0.244)	8.209(0.000-40.949)	-0.296(-64.350-2.785)
Southern Latin America	0.850(0.000-4.263)	-0.440(-0.757--0.084)	8.106(0.000-41.008)	-0.168(-55.707-54.438)	19.426(0.000-97.041)	-0.445(-0.714--0.109)	8.081(0.000-40.865)	-0.128(-49.945-51.425)
Southern Sub-Saharan Africa	0.450(0.000-2.475)	-0.184(-0.817--0.044)	6.302(0.000-34.864)	-7.150(-78.884--0.789)	11.519(0.000-62.182)	-0.212(-0.845--0.079)	6.472(0.000-35.326)	-7.853(-82.321--1.727)
Tropical Latin America	0.782(-0.000-4.012)	-0.506(-0.881--0.426)	7.995(-0.000-40.720)	-1.112(-75.394-14.122)	18.881(-0.000-96.829)	-0.494(-0.814--0.394)	7.949(-0.000-40.444)	-1.211(-63.392-18.828)
Western Europe	0.406(0.000-2.103)	-0.609(-0.881--0.476)	7.257(0.000-38.515)	1.623(-68.463-36.797)	8.935(0.000-45.979)	-0.609(-0.890--0.440)	7.408(0.000-38.930)	2.511(-70.652-44.525)
Western Sub-Saharan Africa	0.488(0.000-2.544)	-0.150(-0.337-0.934)	7.308(0.000-38.597)	2.117(-5.928-126.256)	11.316(0.000-59.283)	-0.193(-0.375-0.809)	7.268(0.000-38.480)	1.872(-9.963-131.150)

DALY, disability adjusted life-years; PAF, population attributable fraction.

**Table 2:** Age-standardized deaths and DALYs attributable to smoking in 2021 and percentage change from 1990 to 2019, by gender, SDI quintile and 21GBD regions.

	Deaths			DALYs				
	2021 age-standardized rate per 100,000 people	Percentage change in age-standardized rate, 1990–2021	2021 age-standardized PAF	Percentage change in agestandardized PAF, 1990–2021	2021 age-standardized rate per 100,000 people	Percentage change in age-standardized rate, 1990–2021	2021 age-standardized PAF	Percentage change in agestandardized PAF, 1990–2021
Global	1.254(0.981-1.605)	-0.553(-0.629--0.459)	11.181(9.283-13.158)	-0.592(-0.664--0.503)	29.006(22.746-37.324)	-12.303(-20.147--3.864)	11.020(9.138-12.851)	-13.289(-21.018--4.752)
Sex								
Male	2.562(1.990-3.298)	-0.547(-0.626--0.446)	15.959(13.456-18.706)	-0.586(-0.662--0.488)	57.567(44.842-74.594)	-14.184(-19.085--8.552)	15.488(13.071-18.018)	-14.434(-18.710--9.376)
Female	0.163(0.129-0.203)	-0.674(-0.717--0.628)	2.292(1.827-2.768)	-0.687(-0.725--0.644)	3.588(2.877-4.421)	-31.233(-37.654--24.333)	2.168(1.753-2.591)	-29.270(-35.139--23.309)
Socio-demographic index								
low	0.357(0.233-0.445)	-0.389(-0.480--0.256)	4.216(3.319-5.078)	-0.423(-0.506--0.303)	8.645(5.720-10.792)	-14.130(-22.995--5.662)	4.114(3.263-4.897)	-14.215(-22.786--5.416)
Low-middle	0.558(0.428-0.686)	-0.437(-0.514--0.336)	7.225(5.886-8.589)	-0.461(-0.532--0.362)	13.640(10.453-16.825)	-23.726(-28.810--17.546)	7.076(5.814-8.346)	-23.227(-28.227--17.336)
Middle	1.633(1.231-2.178)	-0.534(-0.642--0.388)	11.877(9.738-14.233)	-0.585(-0.685--0.452)	37.476(28.132-50.226)	-4.593(-16.876-9.340)	11.669(9.543-13.879)	-6.579(-18.103-6.821)
High-Middle	1.950(1.502-2.548)	-0.510(-0.604--0.392)	13.032(10.866-15.222)	-0.558(-0.644--0.438)	46.469(35.851-61.162)	1.880(-7.594-12.699)	13.129(10.956-15.248)	0.255(-8.182-10.021)
High	0.734(0.598-0.891)	-0.696(-0.722--0.669)	10.743(8.755-12.796)	-0.727(-0.747--0.703)	15.836(12.986-18.913)	-29.505(-33.828--24.706)	10.838(8.980-12.699)	-28.657(-32.676--24.456)
Region								
Andean Latin America	0.754(0.569-1.000)	-0.463(-0.566--0.325)	3.533(2.869-4.311)	-17.547(-24.899--9.110)	17.798(13.240-23.646)	-0.476(-0.581--0.346)	3.664(3.031-4.362)	-16.592(-23.866--8.840)
Australasia	0.238(0.187-0.303)	-0.702(-0.741--0.657)	6.096(4.884-7.499)	-41.050(-46.501--35.045)	5.656(4.548-6.958)	-0.701(-0.735--0.661)	6.663(5.443-8.031)	-39.513(-44.444--34.390)
Caribbean	0.480(0.368-0.595)	-0.499(-0.575--0.417)	6.174(5.033-7.584)	-20.264(-28.630--11.458)	11.567(8.892-14.190)	-0.488(-0.563--0.404)	5.919(4.872-7.203)	-22.431(-30.047--13.807)
Central Asia	1.141(0.918-1.384)	-0.555(-0.615--0.483)	9.871(8.159-11.479)	1.192(-7.474-9.698)	29.302(23.737-35.430)	-0.597(-0.652--0.529)	9.813(8.145-11.409)	-2.824(-10.493-4.757)
Central Europe	0.954(0.773-1.146)	-0.620(-0.655--0.581)	11.163(9.314-13.162)	-17.632(-22.163--13.258)	23.936(19.575-28.657)	-0.626(-0.659--0.588)	11.886(9.978-13.877)	-17.544(-21.711--13.585)
Central Latin America	0.467(0.369-0.579)	-0.669(-0.712--0.624)	3.995(3.282-4.730)	-40.241(-45.322--35.260)	11.443(9.021-14.132)	-0.655(-0.697--0.606)	4.049(3.358-4.760)	-41.082(-45.453--36.931)
Central Sub-Saharan Africa	0.255(0.168-0.343)	-0.389(-0.538--0.185)	2.855(2.200-3.539)	-16.972(-28.514--5.047)	6.883(4.511-9.345)	-0.386(-0.546--0.172)	3.125(2.452-3.853)	-14.555(-26.074--2.341)
East Asia	3.044(2.245-4.209)	-0.502(-0.634--0.321)	14.283(11.767-16.998)	5.754(-8.237-21.741)	70.004(51.492-97.495)	-0.554(-0.682--0.387)	14.050(11.548-16.666)	3.948(-8.514-18.188)
Eastern Europe	1.256(1.030-1.504)	-0.611(-0.659--0.563)	10.334(8.642-12.122)	-4.786(-12.627-2.758)	34.146(28.071-40.608)	-0.635(-0.680--0.591)	11.137(9.380-13.005)	-4.134(-10.976-2.739)
Eastern Sub-Saharan Africa	0.223(0.166-0.280)	-0.475(-0.553--0.356)	3.144(2.544-3.791)	-17.829(-25.007--10.391)	5.591(4.125-7.041)	-0.480(-0.560--0.359)	3.184(2.591-3.835)	-13.260(-21.425--4.552)
High-income Asia Pacific	1.361(1.100-1.683)	-0.770(-0.800--0.737)	10.374(8.425-12.517)	-35.024(-42.432--27.069)	28.051(23.136-33.920)	-0.799(-0.823--0.772)	10.273(8.453-12.158)	-33.770(-40.407--26.324)
High-income North America	0.355(0.278-0.442)	-0.631(-0.664--0.594)	12.029(9.553-14.645)	-30.462(-35.923--23.841)	8.161(6.581-9.935)	-0.644(-0.672--0.612)	11.512(9.356-13.855)	-35.122(-39.629--29.673)
North Africa and Middle East	0.889(0.560-1.112)	-0.470(-0.564--0.356)	9.411(7.549-11.257)	-14.671(-24.452--3.252)	19.840(12.532-24.809)	-0.514(-0.597--0.409)	9.108(7.306-10.842)	-15.057(-24.148--4.454)
Oceania	0.750(0.536-1.020)	-0.378(-0.523--0.198)	5.575(4.492-6.699)	-17.813(-26.778--8.651)	21.349(15.003-29.598)	-0.364(-0.519--0.158)	6.247(5.021-7.524)	-14.967(-23.613--4.678)
South Asia	0.376(0.282-0.481)	-0.485(-0.573--0.365)	6.553(5.290-7.822)	-29.094(-36.264--20.816)	9.041(6.856-11.689)	-0.522(-0.602--0.410)	6.202(5.015-7.395)	-29.182(-36.271--20.938)
Southeast Asia	0.613(0.482-0.786)	-0.480(-0.563--0.334)	8.956(7.329-10.506)	-16.874(-23.941--6.677)	15.306(11.901-19.745)	-0.483(-0.571--0.330)	8.952(7.304-10.521)	-13.670(-20.851--2.840)
Southern Latin America	0.754(0.606-0.921)	-0.539(-0.597--0.477)	7.181(5.873-8.585)	-18.178(-24.063--11.882)	20.036(16.328-24.229)	-0.546(-0.602--0.485)	8.309(6.913-9.780)	-18.577(-23.839--13.001)
Southern Sub-Saharan Africa	0.383(0.294-0.471)	-0.461(-0.539--0.372)	5.365(4.410-6.481)	-38.467(-44.470--31.124)	10.463(8.066-12.849)	-0.444(-0.522--0.357)	5.887(4.856-7.071)	-34.871(-40.709--27.903)
Tropical Latin America	0.741(0.587-0.925)	-0.740(-0.774--0.704)	7.578(6.089-9.294)	-48.101(-54.276--41.832)	17.285(13.934-21.212)	-0.743(-0.772--0.714)	7.267(5.920-8.810)	-49.951(-55.305--44.224)
Western Europe	0.569(0.456-0.688)	-0.727(-0.750--0.706)	10.207(8.205-12.207)	-28.886(-33.401--24.600)	12.747(10.429-15.256)	-0.725(-0.745--0.707)	10.588(8.642-12.488)	-27.901(-31.881--24.288)
Western Sub-Saharan Africa	0.142(0.102-0.182)	-0.306(-0.436--0.149)	2.120(1.722-2.552)	-16.942(-23.774--9.317)	3.545(2.499-4.529)	-0.326(-0.460--0.166)	2.268(1.859-2.711)	-15.169(-21.769--7.357)

DALY, disability adjusted life-years; PAF, population attributable fraction.

**Figure 1:**
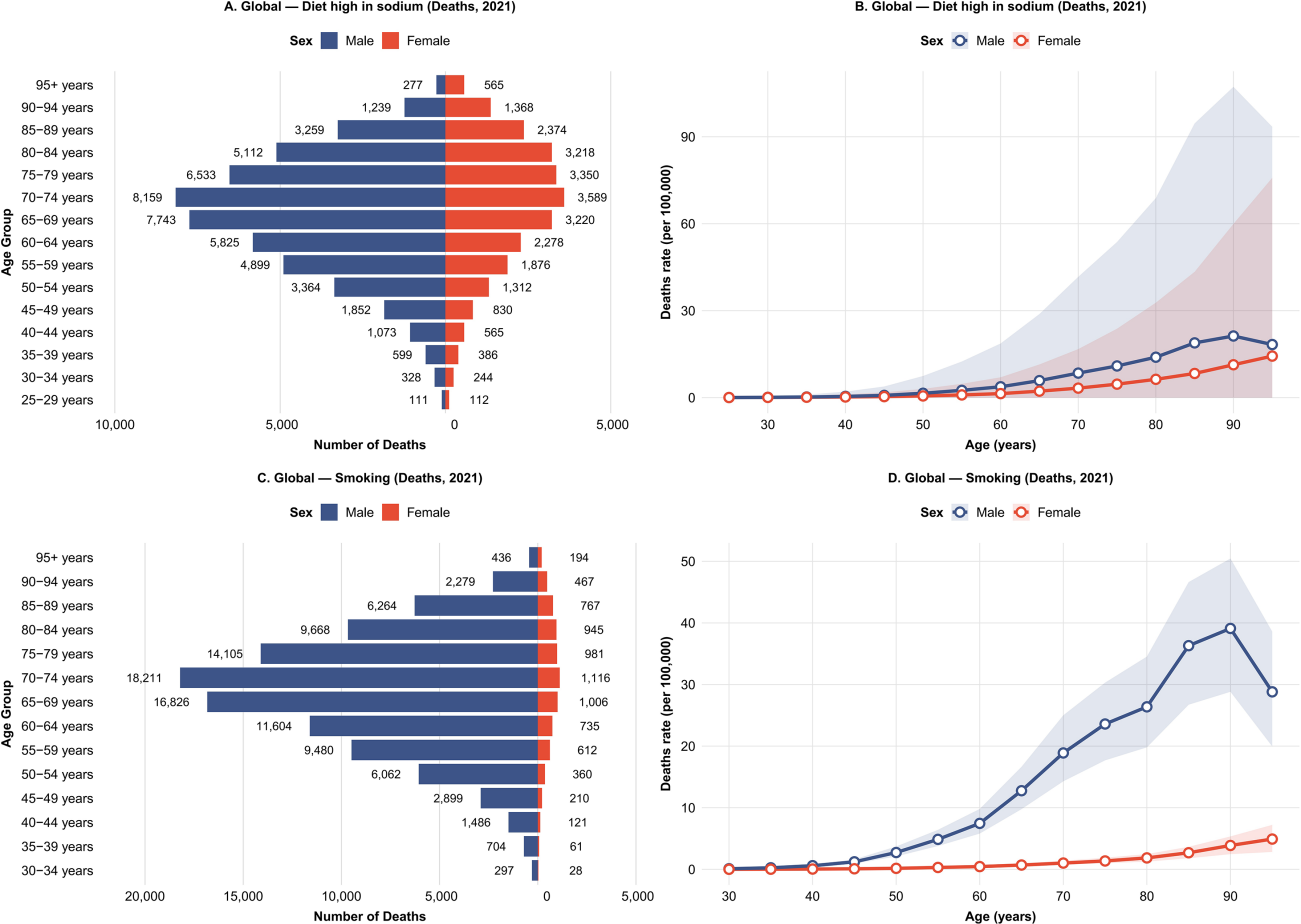
Age-specific numbers (bar plot) and rates (line plot) of deaths attributable to diet high in sodium [**(A)** number of death; **(B)** deaths rate] and smoking [**(C)** number of death; **(D)**: deaths rate] by sex in 2021.

**Figure 2:**
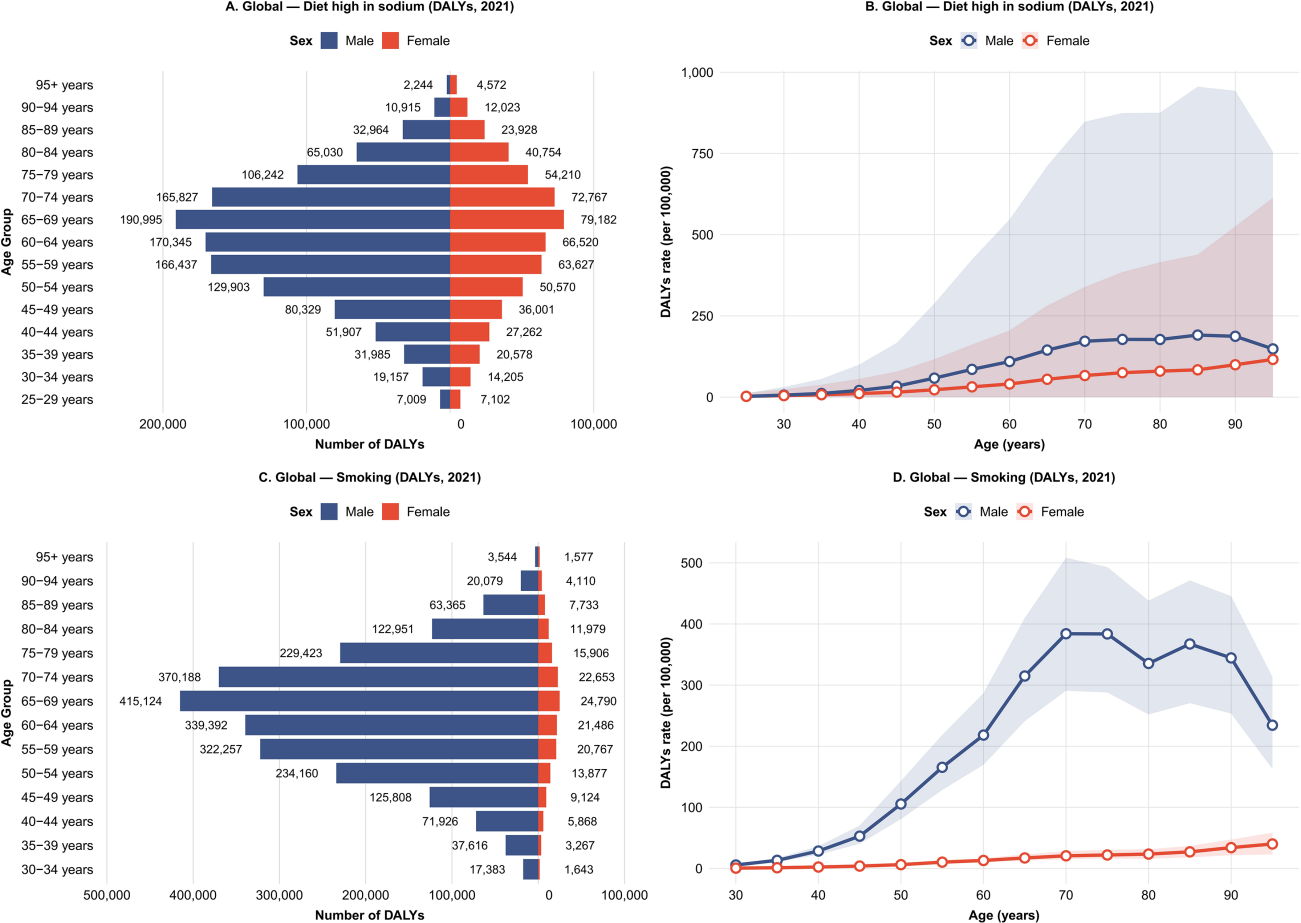
Age-specific numbers (bar plot) and rates (line plot) of DALYs attributable to diet high in sodium [**(A)** number of DALYs; **(B)** DALYs rate] and smoking [**(C)** number of DALYs; **(D)** DALYs rate] by sex in 2021.

In 2021, the age-specific number and rate of deaths attributable to a high-sodium diet are shown in **Figures 1B**, while the corresponding number and rate of DALYs are shown in **Figure 2**. The number of deaths and DALYs increased with age for both sexes, with the highest counts observed in the 70–74 age group for deaths and the 65–69 age group for DALYs. Males consistently showed higher counts than females across nearly all age groups. Similarly, the death rate and DALY rate attributable to high sodium intake rose with age, with the highest death rate observed in the 90–94 age group and the highest DALY rate in the 85–89 age group.

In 2021, the age-specific number and rate of deaths attributable to smoking are shown in **Figure 1D**, while the corresponding number and rate of DALYs are shown in **Figure 2**. The number of deaths and DALYs increased with age for both males and females, with the peak number of deaths occurring in the 70–74 age group and the peak DALYs in the 65–69 age group. Males showed consistently higher counts of deaths and DALYs than females across nearly all age groups. The death rate and DALY rate due to smoking also rose with age, reaching the highest rates in the 90–94 age group for deaths and the 70–74 age group for DALYs.

### 3.2 Regional variation in mortality and DALYs attributable to diet high in sodium and smoking in 2021

Across the 21 GBD regions in 2021, the highest age-standardized death rates of diet high in sodium and smoking were observed in the East Asia region (1.707 [95% UI 0.000, 8.649]) and (3.044 [95% UI 2.245, 4.209] per 100,000 population) respectively (**Tables 1**, **2**; **Supplementary Figures 1**, **S2B**). The highest DALYs rates were also observed in the East Asia region (41.092 [95% UI 0.000, 206.627] and (70.004 [95% UI 51.492, 97.495] per 100,000 population) respectively (**Tables 1**, **2**; **Supplementary Figures 1**, **S2D**).

The lowest age-standardized death rates of diet high in sodium and smoking were observed in the High-income North America region (0.227[95% UI 0.000, 1.172]) and in the Western Sub-Saharan Africa (0.142 [95% UI 0.102, 0.182] per 100,000 population) respectively (**Tables 1**, **2**; **Supplementary Figures 1**, **S2B**). The lowest DALYs rates were also observed in the High-income North America region 5.522 [95% UI 0.000, 28.105] and in the Western Sub-Saharan Africa (3.545 [95% UI 2.499, 4.529] per 100,000 population) respectively (**Tables 1**, **2**; **Supplementary Figures 1** and **S2D**).

**Figure 3:**
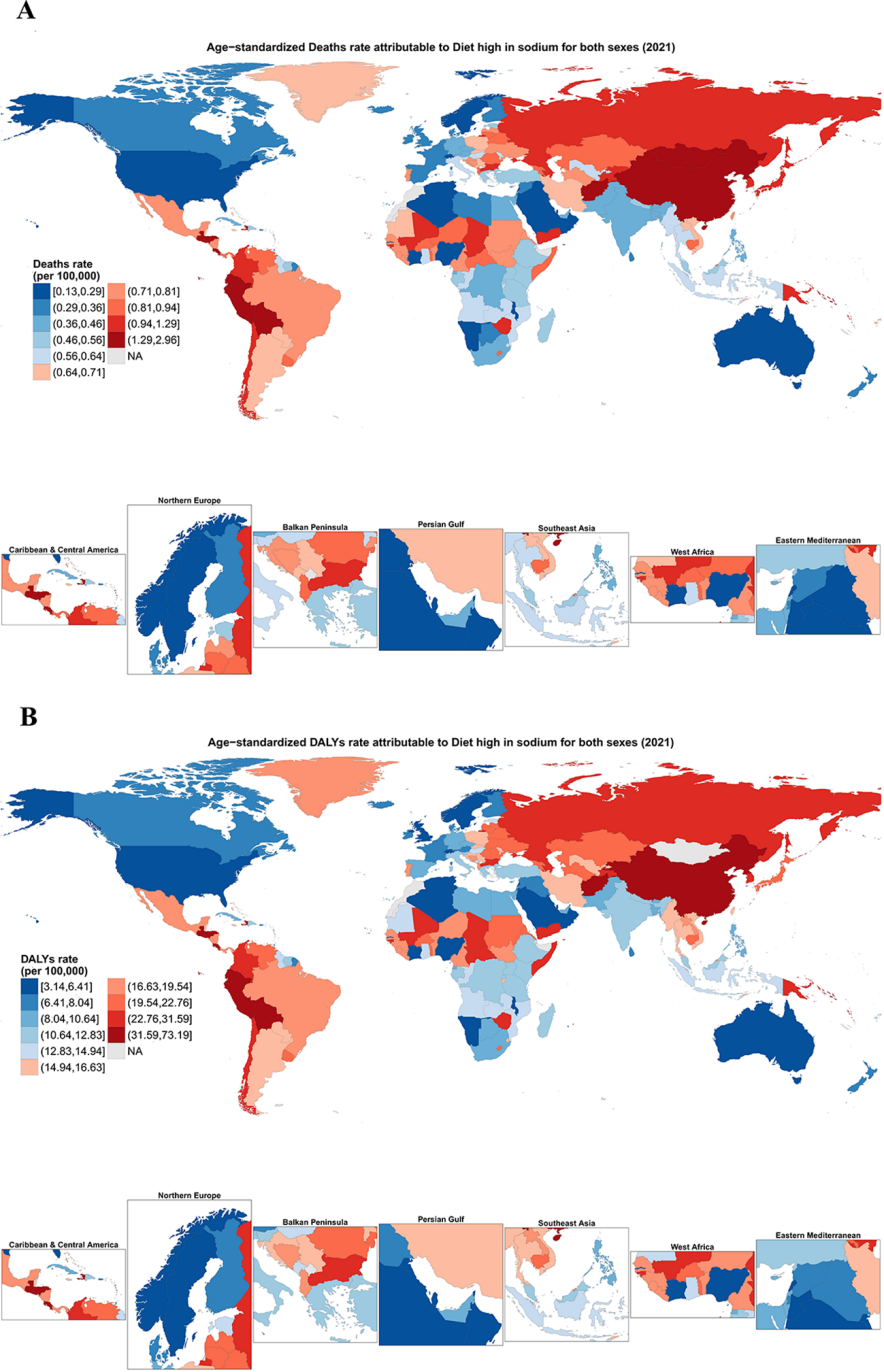
Age-standardized death and DALY rates attributable to diet high in sodium for both sexes combined in 2021. **(A)** deaths. **(B)** DALYs.

**Figure 3A** show the age-standardized death and DALY rates per 100,000 population attributable to a diet high in sodium across countries and territories. Higher death rates were indicated by darker shades of red, while lower rates were shown in blue shades. In 2021, the countries with the highest age-standardized death and DALY rates attributable to a diet high in sodium included Mongolia, with a death rate of 2.956 and a DALY rate of 73.195 per 100,000 people, and Bolivia, with a death rate of 2.584 and a DALY rate of 56.468 per 100,000 people. Conversely, countries with the lowest sodium-related death rates included Morocco and Namibia, with death rates of 0.127 and 0.175 per 100,000 people, respectively. The lowest sodium-related DALY rates were observed in Morocco and Nigeria, with DALY rates of 3.139 and 4.108 per 100,000 people, respectively (**Table 1**; **Figure 3**).

**Figure 4:**
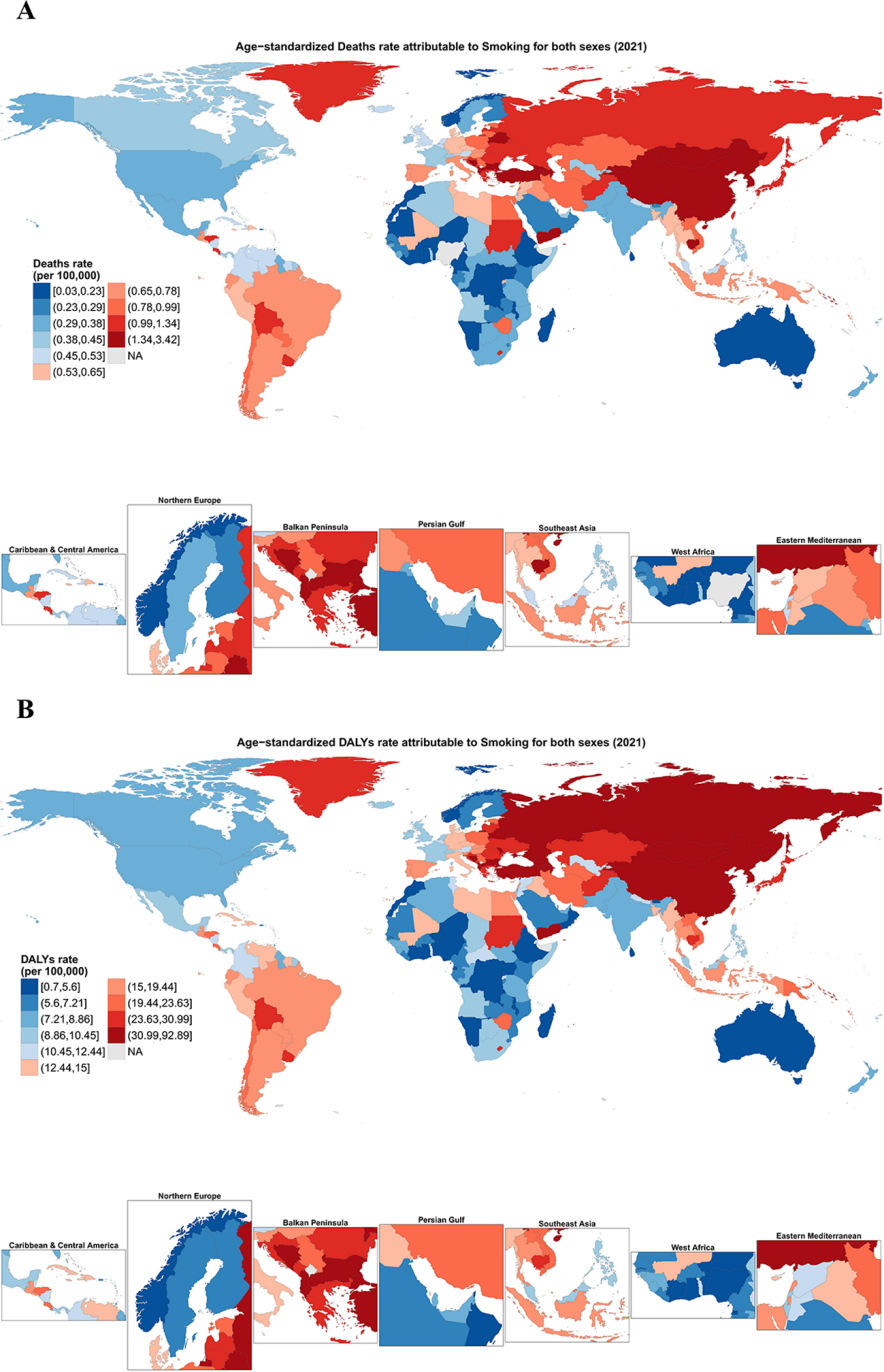
Age-standardized death and DALY rates attributable to smoking for both sexes combined in 2021. **(A)** deaths. **(B)** DALYs.

**Figures 4B** show the age-standardized death and DALY rates per 100,000 population attributable to smoking across countries and territories. Countries with the highest death rates included Mongolia, with a death rate of 3.417 and a DALY rate of 92.85 per 100,000 people, and China, with a death rate of 3.097 and a DALY rate of 71.071 per 100,000 people. Conversely, countries with the lowest smoking-related death and DALY rates included Nigeria, with a death rate of 0.028 and a DALY rate of 0.700 per 100,000 people, and Côte d’Ivoire, with a death rate of 0.090 and a DALY rate of 2.323 per 100,000 people, respectively (**Table 2**; **Figure 4**).

**Figure 1** illustrated the average annual percentage change (AAPC) in age-standardized death rates attributable to diet high in sodium across various countries from 1990 to 2021. Countries shaded in darker red experienced the highest increases in death rates, with Egypt showing an AAPC of 0.487 per 100,000 people. In contrast, countries shaded in blue showed a decline in death rates, with South Korea experiencing the largest decrease, with an AAPC of -0.759 per 100,000 people. **Supplementary Figure 10** showed the AAPC in age-standardized DALY rates attributable to diet high in sodium over the same period. Lesotho experiencing the largest increase, with an AAPC of 0.371 per 100,000 people. South Korea experiencing the largest decrease, with an AAPC of -0.797 per 100,000 people.

**Figure 1** illustrated the average annual percentage change (AAPC) in age-standardized death rates per 100,000 population attributable to smoking across various countries from 1990 to 2021. Egypt experiencing the largest increase, with an AAPC of 0.681 per 100,000 people. Singapore experiencing the largest decrease, with an AAPC of -0.849 per 100,000 people.

**Figure 1** showed the AAPC in age-standardized DALYs rates per 100,000 population attributable to smoking over the same period. Lesotho experiencing the largest increase, with an AAPC of 0.564 per 100,000 people. Singapore experiencing the largest decrease, with an AAPC of -0.864 per 100,000 people. These supplementary AAPC maps provide country-level trend information and help identify locations with increasing or decreasing attributable burden over the study period.

### 3.3 Relationship between SDI and the impact of diet high in sodium and smoking on disease burden

In 2021, the highest age-standardized rates of deaths related to diet high in sodium and smoking were observed in the high-middle SDI quintile, with 1.184 [95% UI 0.000, 5.791] deaths per 100,000 people for diet high in sodium and 1.950 [95% UI 1.502, 2.548] deaths per 100,000 people for smoking (**Tables 1**, **2**). The highest related DALYs were also seen in the high-middle SDI quintile, with 28.139 [95% UI 0.000, 136.923] DALYs per 100,000 people for diet high in sodium and 46.469 [95% UI 35.851, 61.162] DALYs per 100,000 people for smoking (**Tables 1**, **2**).

The lowest age-standardized death rates for diet high in sodium and smoking were found in the high SDI quintile, with 0.543 [95% UI 0.000, 2.733] deaths per 100,000 people for diet high in sodium, and in the low SDI quintile, with 0.357 [95% UI 0.233, 0.445] deaths per 100,000 people for smoking (**Tables 1**, **2**). The lowest age-standardized DALYs for diet high in sodium and smoking were also observed in the high SDI quintile, with 11.603 [95% UI 0.000, 58.092] DALYs per 100,000 people for diet high in sodium, and in the low SDI quintile, with 8.645 [95% UI 5.720, 10.792] DALYs per 100,000 people for smoking (**Tables 1**, **2**).

**Supplementary Figure 6** showed the age-standardized death rates per 100,000 population attributable to diet high in sodium (A) and smoking (B) globally and across five SDI groups from 1990 to 2021. Across all SDI regions, both high sodium and smoking-related death rates generally declined over time. The high-middle and middle SDI region consistently had higher death rates for both high sodium intake and smoking, while the low SDI region demonstrated lower death rates.

**Supplementary Figure 7** presented the age-standardized DALY rates per 100,000 population attributable to diet high in sodium (A) and smoking (B) globally and across the same five SDI groups over the same period. Similar to death rates, DALYs rates decreased across all SDI regions from 1990 to 2021. High-middle SDI regions had the highest DALYs rates for both high sodium intake and smoking, while low SDI regions had the lowest DALYs rates. Together, **Supplementary Figures 6**, **S7** show that high-middle and middle SDI regions consistently carried higher attributable burdens, although overall rates declined over time across SDI groups.

### 3.4 Prediction of age standardized rate by RStudio

**Supplementary Figure 8** showed the projected global death rates per 100,000 people from stomach cancer attributable to diet high in sodium (A) and smoking (B) from 2021 to 2040. The trend indicated a continued decline in death rates, with both factors contributing to a reduced burden of stomach cancer over the forecast period. Shaded regions around the trend lines represented uncertainty intervals, indicating potential variability in the estimates.

**Supplementary Figure 9** presented the forecast for global DALYs rates per 100,000 people from stomach cancer attributable to diet high in sodium (A) and smoking (B) from 2021 to 2040. Like death rates, DALYs rates were projected to decline steadily across the forecast period. Uncertainty intervals in shaded areas showed possible ranges in the forecast, highlighting confidence levels around the decreasing trend.

## 4 Discussion

Overall, these findings suggest several overarching patterns. First, stomach cancer burden attributable to high sodium intake and smoking remains concentrated among older adults, males, East Asia, and high-middle SDI regions. Second, although global age-standardized rates have generally declined, the persistence of high burdens in specific regions indicates that prevention progress has been uneven. Third, the projected decline to 2040 should be interpreted as a continuation of historical trends rather than evidence that no further policy action is needed. These patterns support the need for targeted sodium reduction, tobacco control, and screening strategies in high-burden populations.

### 4.1 Age and gender-related vulnerabilities in stomach cancer attributable to high sodium intake and smoking

Stomach cancer deaths attributed to high sodium intake and smoking increase with age for several reasons. Firstly, both high sodium intake and smoking have cumulative effects on the body. Over time, a diet high in sodium can severely damage the gastric mucosa, leading to hyperplasia of the gastric pit epithelium and increasing the probability of endogenous mutations that may progress to cancer (16). High salt intake can also accelerate the process of intestinal metaplasia, a precursor to early gastric cancer (16). Tobacco products contain carcinogens associated with gastric adenocarcinoma. These carcinogens make direct contact with the stomach mucosa when swallowed with saliva or bronchial secretions and indirectly reach the stomach via the bloodstream (17). Therefore, prolonged smoking results in extended exposure to these carcinogens, increasing the likelihood of developing stomach cancer over time. Secondly, aging is associated with physiological changes, such as weakened immune responses and slower cell repair processes (18), which make older individuals more susceptible to cancer development. High sodium intake and smoking can lead to chronic inflammation and cellular damage in the stomach lining (16, 19), and age-related declines in cellular repair further amplify this risk.

Stomach cancer deaths attributed to high sodium intake and smoking were found to be higher among males, which could be due to a combination of behavioral and biological factors. Males are generally more likely to smoke (20) and tend to consume diets with higher sodium content (21), which may partly explain the elevated rates of stomach cancer deaths among men. Additionally, biological differences between males and females, such as variations in hormone levels and immune responses, may affect susceptibility to the carcinogenic effects of sodium and smoking, potentially making males more vulnerable (22). Although the mechanisms underlying these sex differences remain unclear, further research may help identify more targeted prevention strategies. Furthermore, a previous study indicated that females were less vulnerable to the harmful effects of high sodium intake, as they maintained a stronger ability to regulate sodium balance even when adapting to high-salt diets (23). This physiological resilience among women suggested that future sodium reduction policies targeting men could be particularly beneficial, as men appeared more vulnerable to the adverse effects of high sodium intake.

### 4.2 Regional disparities in stomach cancer rates: the impact of high sodium diets and smoking in East Asia

East Asia had the highest stomach cancer death and DALYs rates attributable to diets high in sodium and smoking. In contrast, the lowest rates were found in High-Income North America and Western Sub-Saharan Africa regions. Several explanations were proposed: Firstly, traditional East Asian diets often include high-sodium foods such as pickled vegetables, soy sauce, and salted fish. This is especially pronounced in Mongolia, where harsh climates and limited agricultural output have led to a heavy reliance on processed and preserved foods, including salted tea, sausage, smoked meats, and local fast foods like Buuz (steamed dumplings) and Khuushuur (fried dumplings). A national study of a representative Mongolian sample in 2011 found that the average daily salt intake was 11.06 ± 5.99 g, more than double the World Health Organization’s recommendation of five grams (24). Tea alone accounted for 30% of total salt intake, with the remainder coming from meals and other processed foods (24). Additionally, the scarcity of fresh fruits and vegetables further exacerbates the dependence on salt-preserved foods in the region (25). Secondly, East Asia faces one of the world’s most significant tobacco epidemics, with nearly half of men in the region being cigarette smokers (26). China and Japan rank as the largest and fifth-largest tobacco-consuming countries worldwide (26).

In addition to sodium intake and smoking, regional disparities in stomach cancer burden may also reflect other etiological and healthcare-system factors. Helicobacter pylori infection remains a major risk factor for gastric cancer and may promote carcinogenesis through chronic inflammation, oxidative stress, DNA damage, and dysregulation of cancer-related signaling pathways (27). High sodium exposure may further aggravate this process by damaging the gastric mucosa, increasing inflammation, and enhancing the pathogenic effects of H. pylori (5). Alcohol use, obesity, genetic susceptibility, and other lifestyle-related factors may also contribute to regional differences in stomach cancer risk (4, 28). In addition, differences in early detection, endoscopic screening coverage, and timely access to treatment may partly explain why stomach cancer mortality and DALYs vary across regions (29). Therefore, the higher burden observed in East Asia should not be interpreted as being driven only by sodium intake and smoking, but rather as the result of multiple interacting risk factors and healthcare-system differences.

Therefore, future preventive strategies should prioritize policies that promote low-sodium diets. Strategies may include front-of-pack labeling schemes, taxation on high-salt foods, educating the public on lower-sodium options, and collaborating with the food industry to create culturally acceptable alternatives (30). Governments can implement large-scale education campaigns that focus on the link between high-sodium diets, smoking, and stomach cancer. Additionally, early screening programs, especially endoscopy, which remains the gold standard for detecting stomach cancer, would be crucial in identifying high-risk individuals (31). Advanced high-resolution endoscopic technologies are showing potential in diagnosing mucosal lesions and improving biopsy accuracy (31). Moreover, biomarker-based approaches, such as pepsinogen level testing, are being explored as cost-effective screening tools, particularly for early detection of intestinal metaplasia (31).

### 4.3 Contributing factors to the high stomach cancer burden in high-middle SDI regions

The high-middle SDI regions had the highest burden from both factors. The potential reasons for this could be attributed to advanced healthcare access. Improved healthcare infrastructure in high-middle SDI regions enabled higher screening rates and better diagnostic accuracy, allowing clinicians to attribute cancer deaths more precisely to factors like high sodium intake and smoking. This increased detection capability made the cancer burden in these regions more visible. In contrast, in low SDI regions, limited healthcare infrastructure and low screening rates (32) resulted in underreporting, and diet and smoking impacts on cancer were less understood. High SDI regions benefited from high screening rates, available treatment options, and comprehensive public health campaigns that helped prevent stomach cancer at early stage (33).

Secondly, in high-middle SDI regions, the rapid economic growth and urbanization led to greater availability of processed and convenience foods high in sodium, which became affordable and accessible dietary staples (34). Unlike low SDI regions, where limited infrastructure and lower income levels may restrict the access to processed foods, high-middle SDI regions faced a shift in dietary patterns that blended traditional, sodium-rich preserved foods with modern, processed options, amplifying sodium intake (35). However, the infrastructure in these regions often lacks sufficient support for healthy lifestyles, and healthcare services are frequently unable to detect diseases early or intervene promptly (31). High SDI regions managed this shift better through regulatory measures, such as sodium limits on processed foods and awareness programs, helping to mitigate cancer risks from high sodium intake. Therefore, comprehensive measures should be planned and implemented in high-middle SDI regions to prevent further increases in disease burden and to strengthen healthcare services.

Future strategies for high-middle SDI regions should emphasize strengthening preventive health campaigns that promote low-sodium diets and smoking cessation programs by raising public awareness about the cancer risks associated with high sodium intake and smoking. Expanding healthcare infrastructure to support accessible screening programs, with investments in endoscopic and biomarker-based tools, would enhance early detection and intervention. Additionally, establishing support systems that encourage healthy lifestyles, such as public recreational spaces and community programs focused on nutrition, is recommended to help address modifiable cancer risk factors effectively.

### 4.4 Limitations

Several limitations should be acknowledged. Firstly, this study was based on GBD estimates rather than individual-level clinical data; therefore, the findings reflect population-level associations and cannot establish individual causality. Some estimates, especially those for high sodium intake, had wide uncertainty intervals. This likely reflects uncertainty in exposure measurement, risk attribution, and data availability across locations. Such wide intervals reduce the precision of these estimates and indicate that comparisons between regions or countries should be interpreted cautiously. Secondly, although high sodium intake and smoking were evaluated as major attributable risks, other important factors, such as H. pylori infection, alcohol use, obesity, genetic susceptibility, screening coverage, and treatment access, were not fully incorporated into the main attribution analysis. Thirdly, the estimation models used for projections to 2040 relied on historical data from 1990 to 2021 and included assumptions that might not have accounted for future shifts in dietary habits, smoking prevalence, healthcare access, or policy interventions. As a result, the projections may overestimate or underestimate future burden if major sodium reduction policies, smoking cessation programs, screening expansion, or healthcare improvements occur during the projection period. Lastly, the discussion section primarily proposed plausible underlying explanations for observed trends and results, based on the analysis of GBD data. It was crucial to note that these explanations were not intended to investigate potential macro-level associations and relationships.

## 5 Conclusion

The findings revealed that high sodium intake and smoking were major contributors to stomach cancer, especially in high-middle SDI regions and East Asia, underscoring the need for targeted interventions like sodium reduction, anti-smoking policies, and improved healthcare. Region-specific, gender-sensitive strategies were recommended to address these risk factors and support policymakers in mitigating this global health burden.
