# Supplementary material for: Global stomach cancer burden from high-sodium diet and smoking: 1990–2021 findings & 2040 projections
Source: Front Oncol. 2026 Jun 25;16:1838243. doi: 10.3389/fonc.2026.1838243 (PMC13347218; doi:10.3389/fonc.2026.1838243)
Supplement: Supplementary file 1 [file DataSheet1.docx]

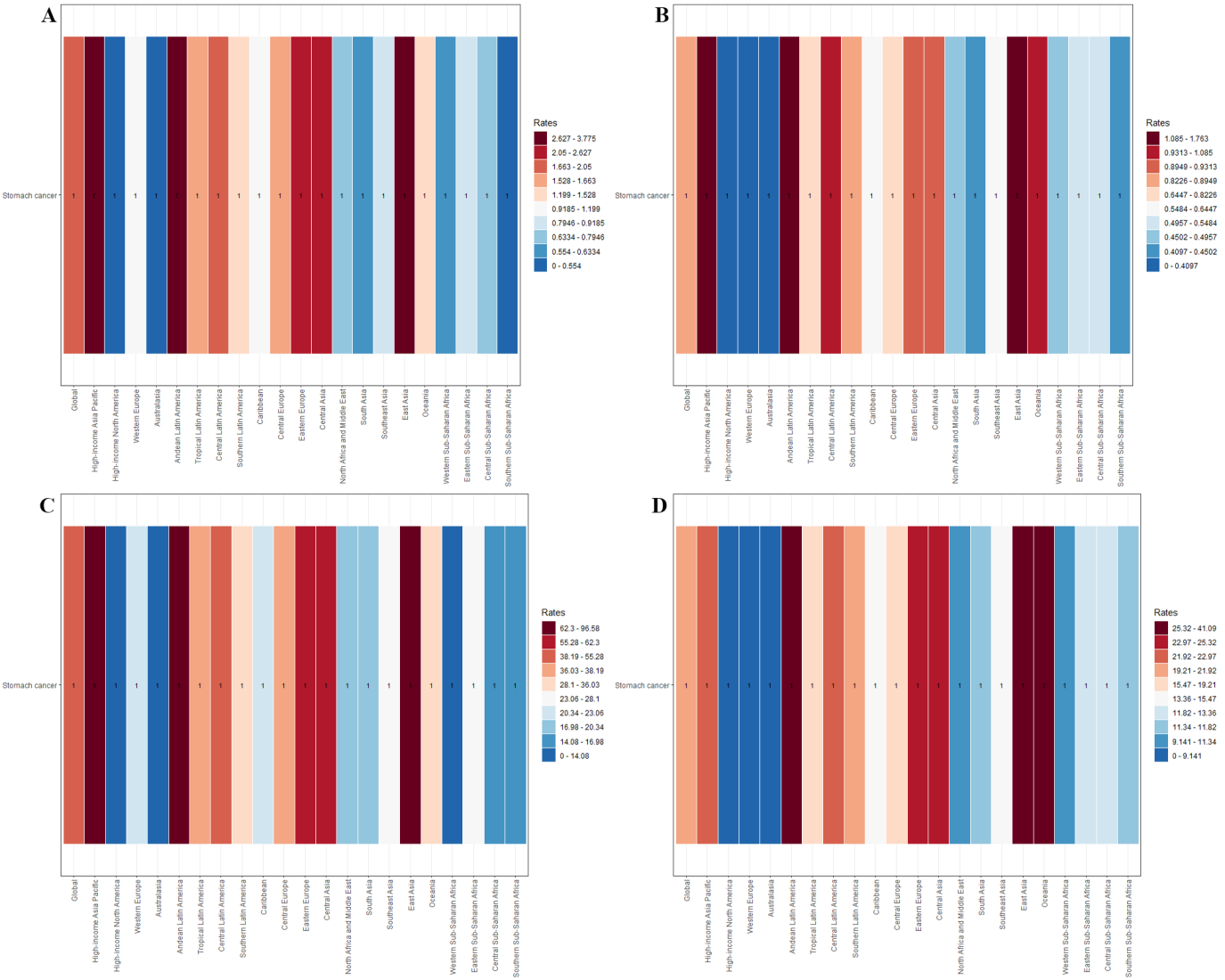


Figure S1. Age-standardized deaths (A: 1990; B: 2021) and DALYs(C: 1990; D: 2021) attributable to diet high in sodium across 21 GBD regions for both sexes.


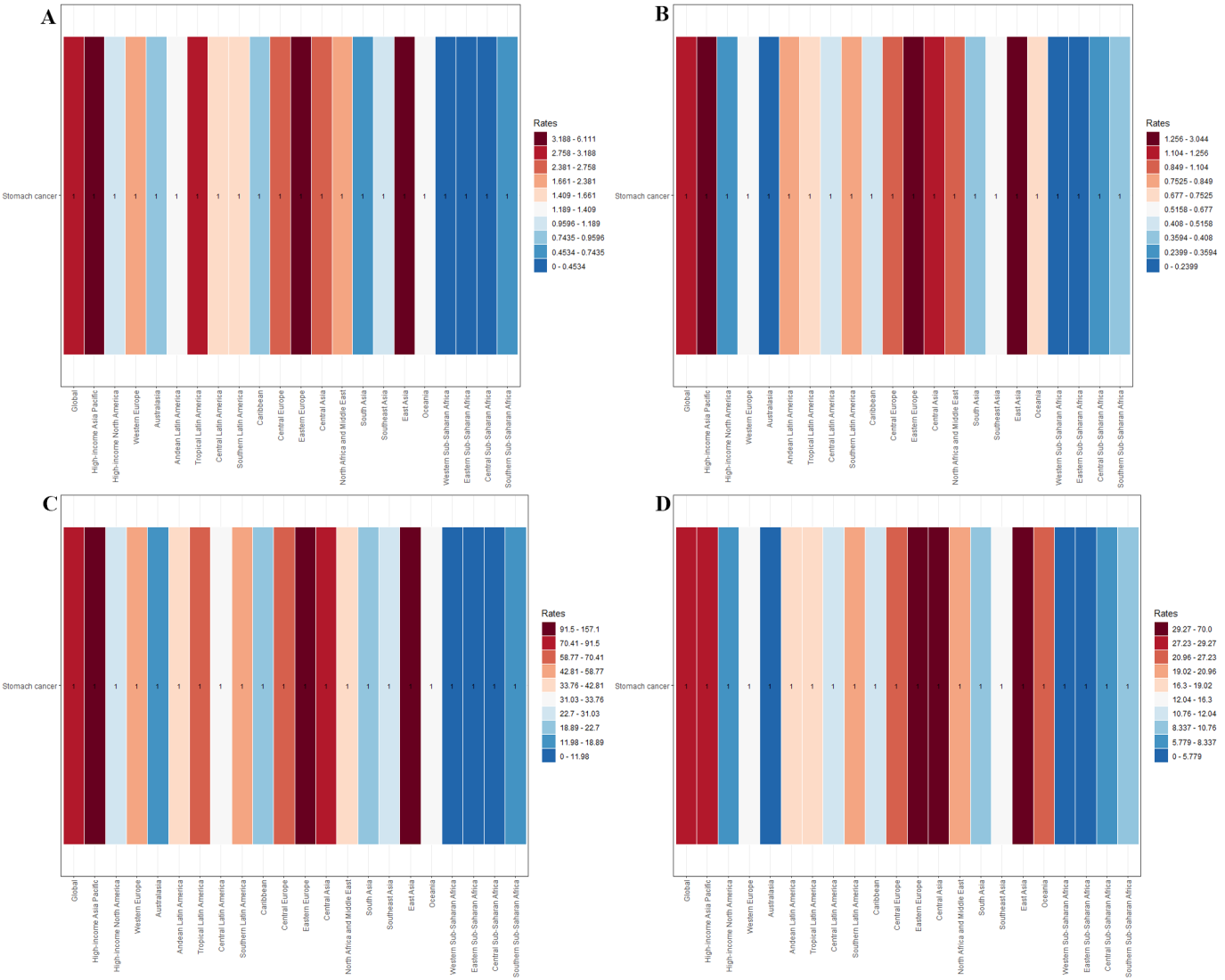


Figure S2. Age-standardized deaths(A: 1990; B: 2021) and DALYs(C: 1990; D: 2021) attributable to smoking across 21 GBD regions for both sexes.


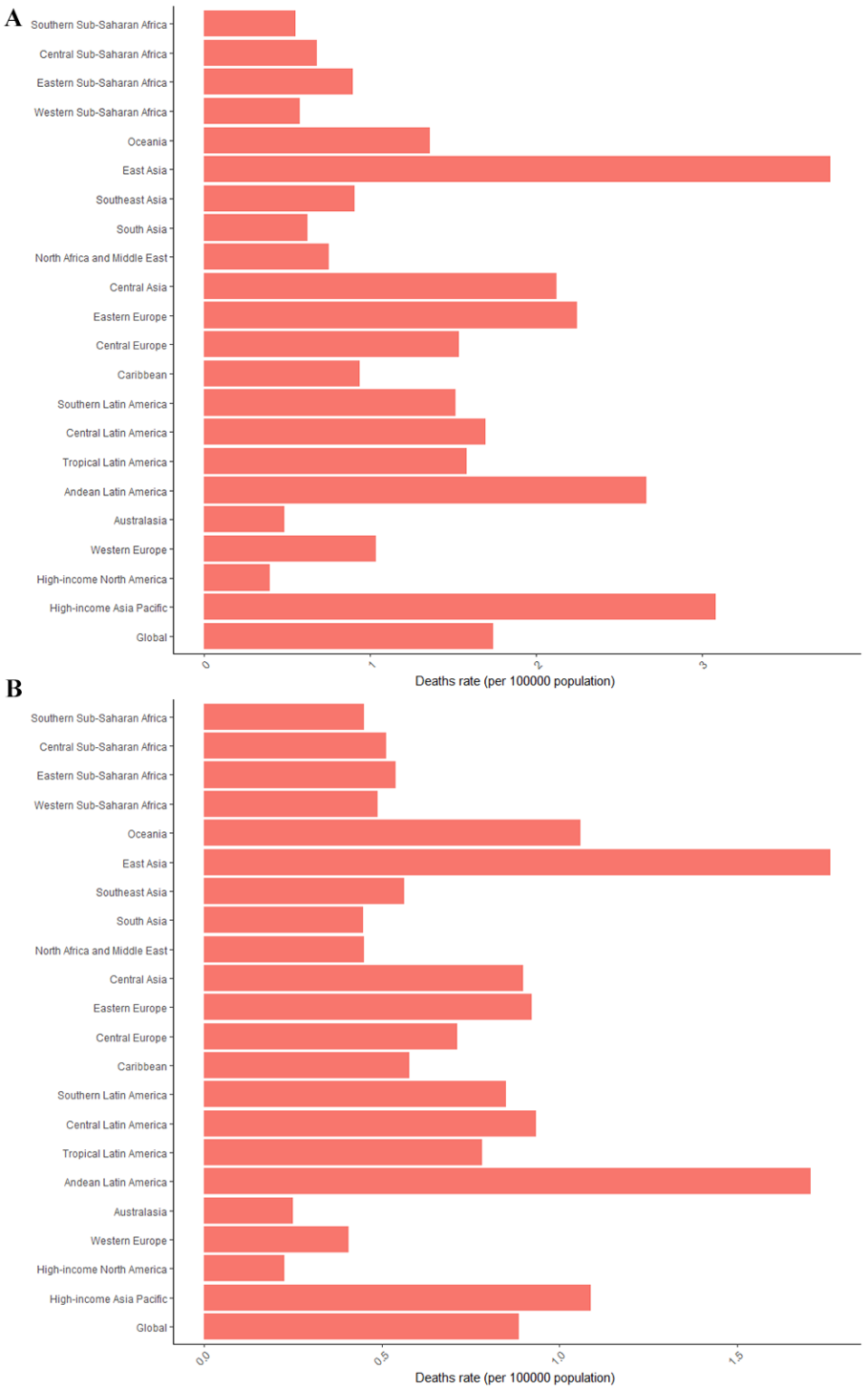


Figure S3. Age-standardized deaths attributable to diet high in sodium across 21 GBD regions for both sexes combined in (A)1990; (B)2021


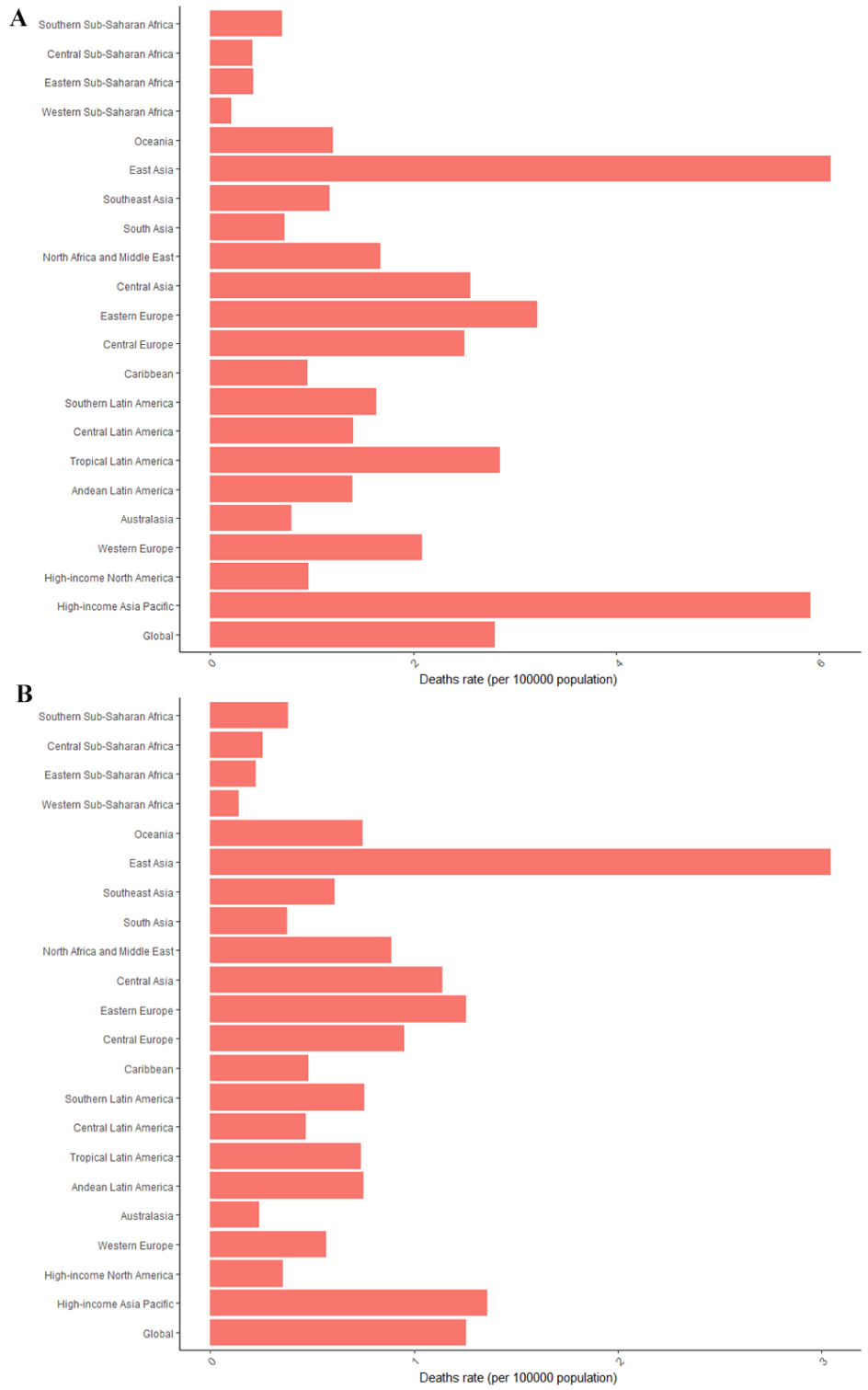


Figure S4. Age-standardized deaths attributable to smoking across 21 GBD regions for both sexes combined in (A)1990; (B)2021


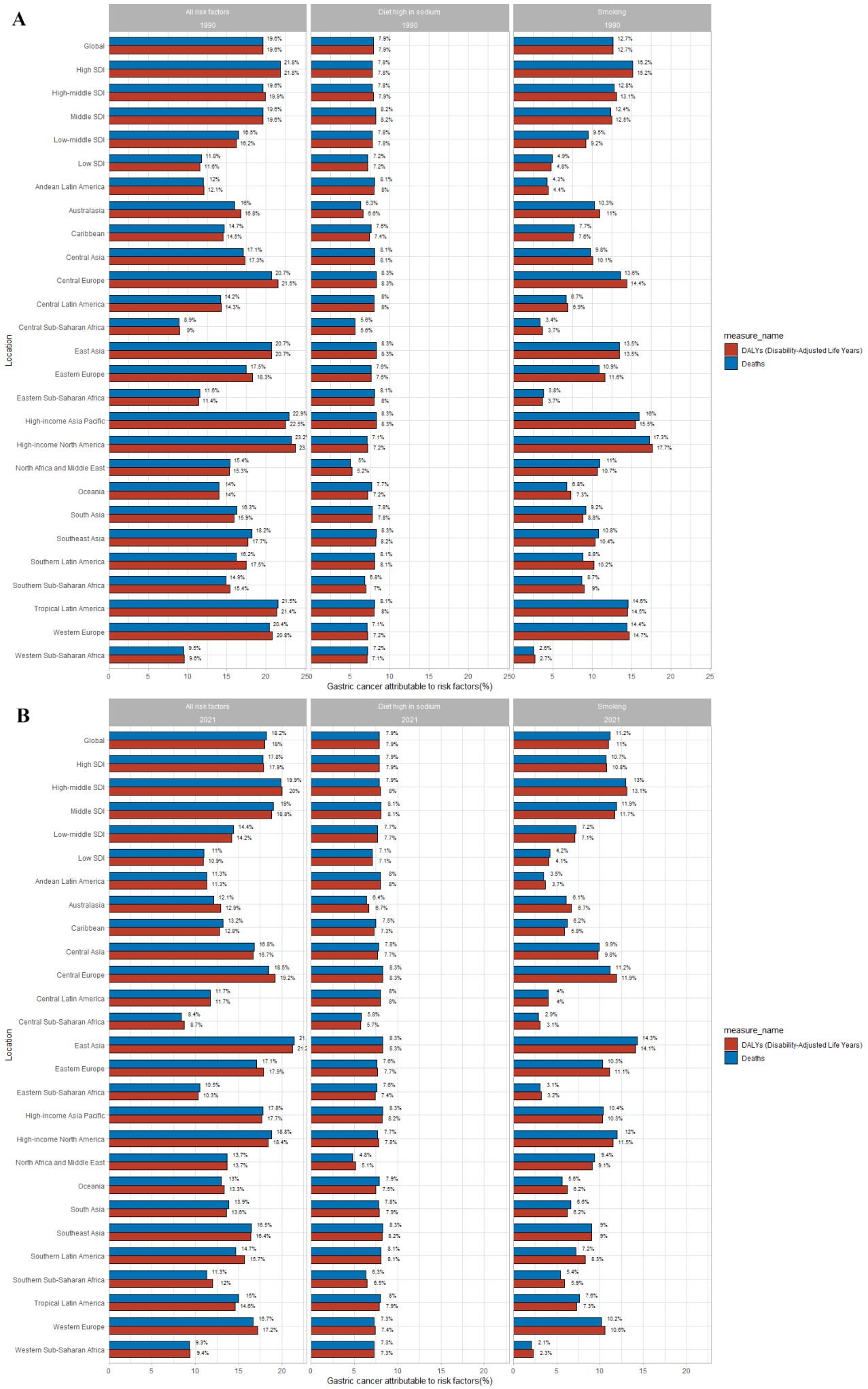


Figure S5. Age-standardized deaths and DALYs attributable to diet high in sodium and smoking across 5 SDI and 21 GBD regions for both sexes combined in (A)1990; (B)2021.

A


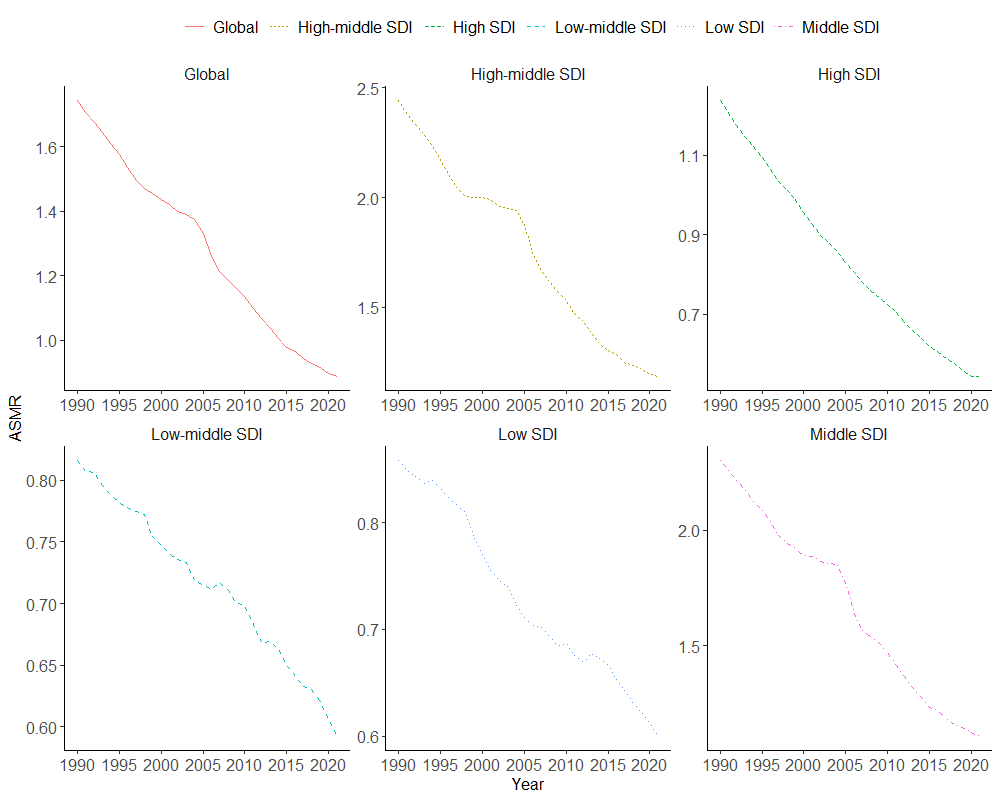


B


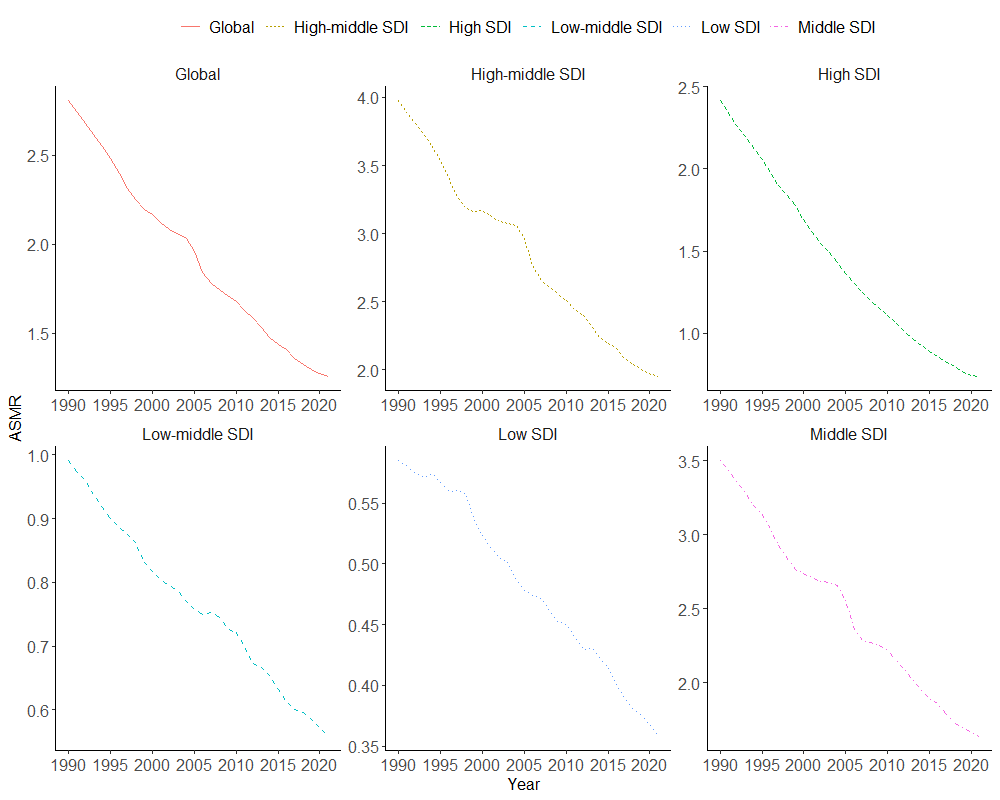


Figure S6. Age-standardized death rates attributable to diet high in sodium and smoking across 5 SDI regions for both sexes combined 1990-2021. (A) diet high in sodium; (B) smoking.

A


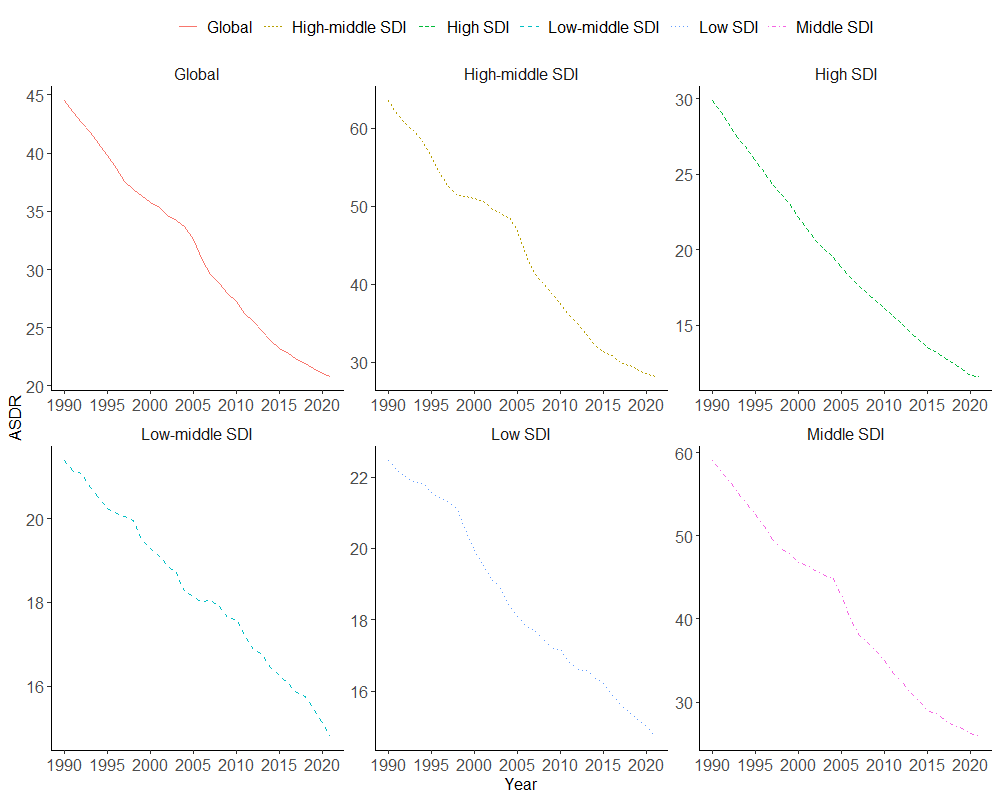


B


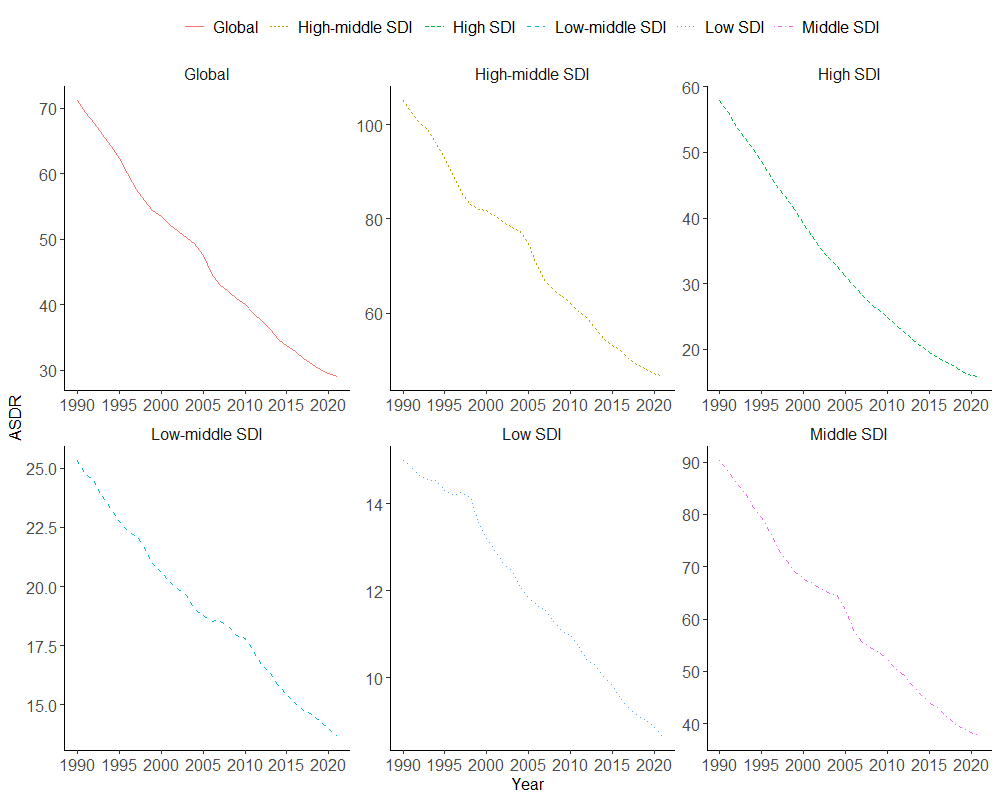


Figure S7. Age-standardized DALY rates attributable to diet high in sodium and smoking across 5 SDI regions for both sexes combined 1990-2021. (A) diet high in sodium; (B) smoking.

A


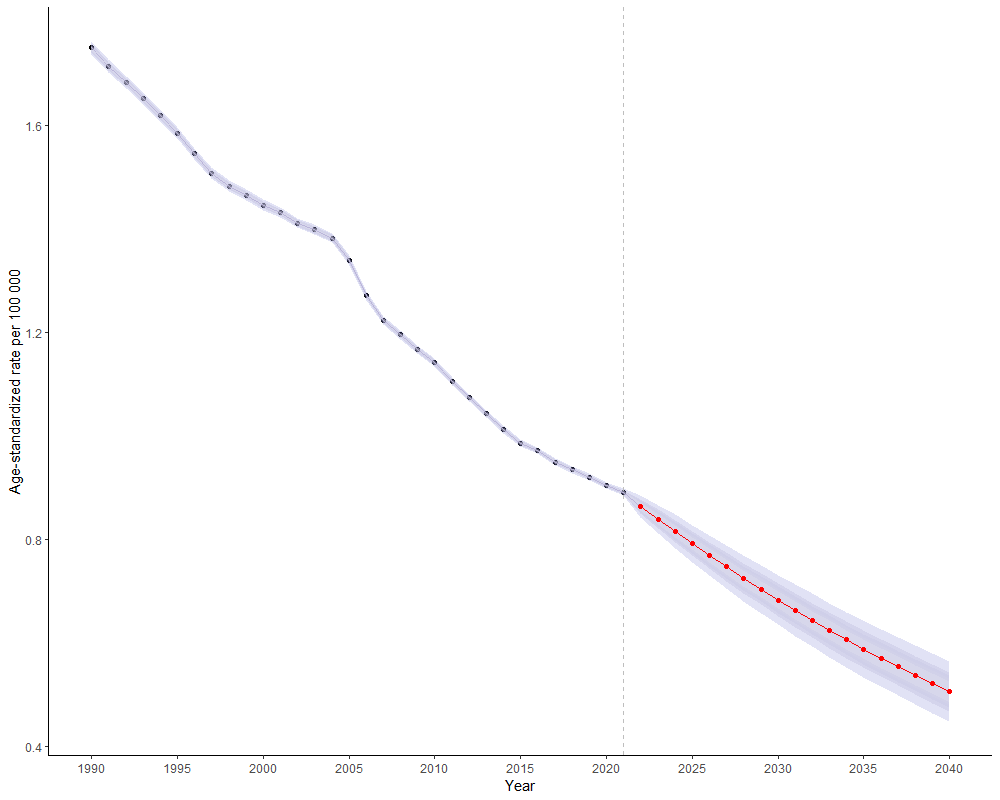


B


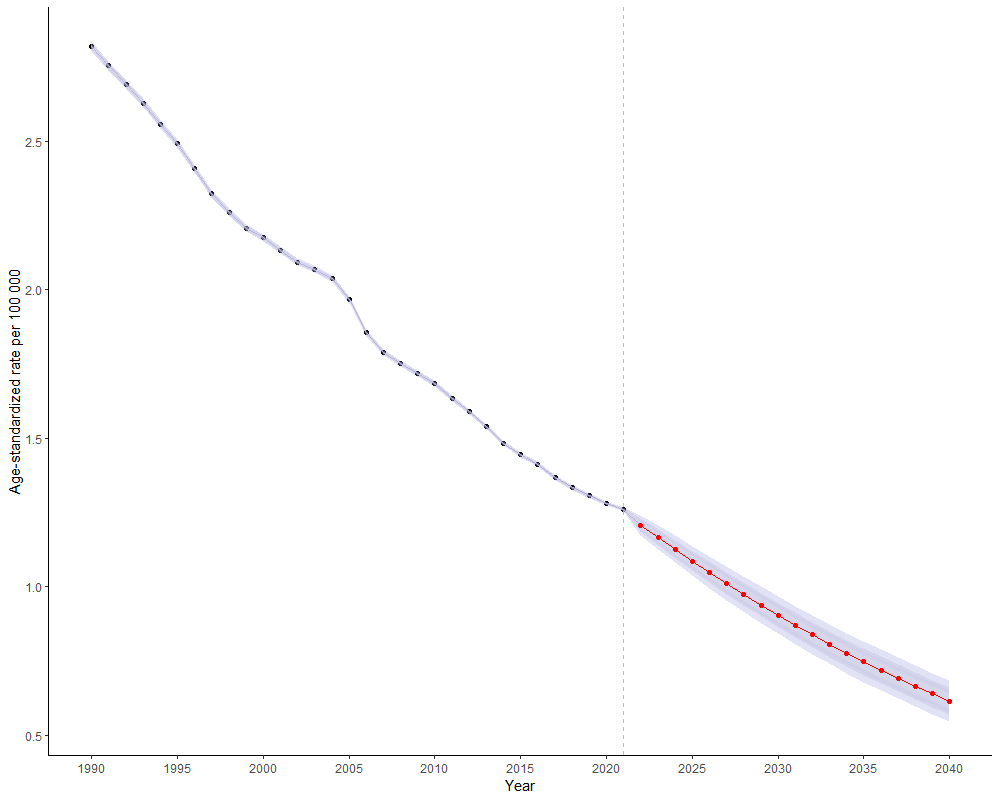


Figure S8. Forecast of the global burden of stomach cancer death rates attributable to diet high in sodium and smoking from 2021 to 2040 (per 100,000 people). (A) diet high in sodium; (B) smoking.

A


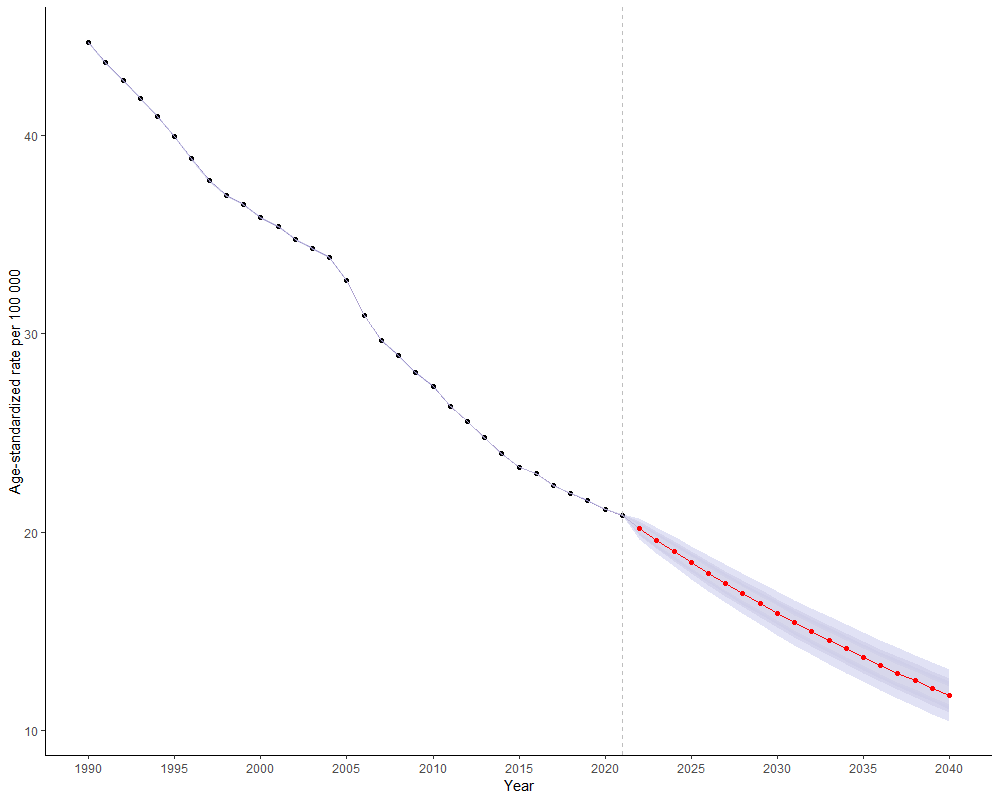


B


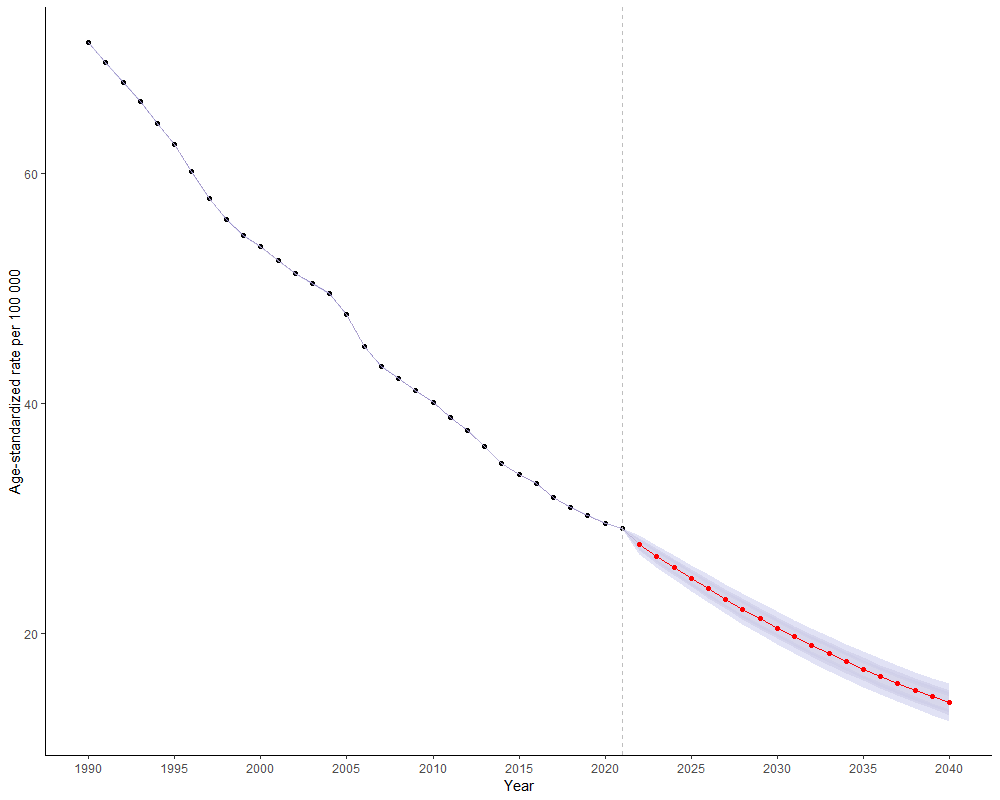


Figure S9. Forecast of the global burden of stomach cancer DALY rates attributable to diet high in sodium and smoking from 2021 to 2040 (per 100,000 people). (A) diet high in sodium; (B) smoking.

A


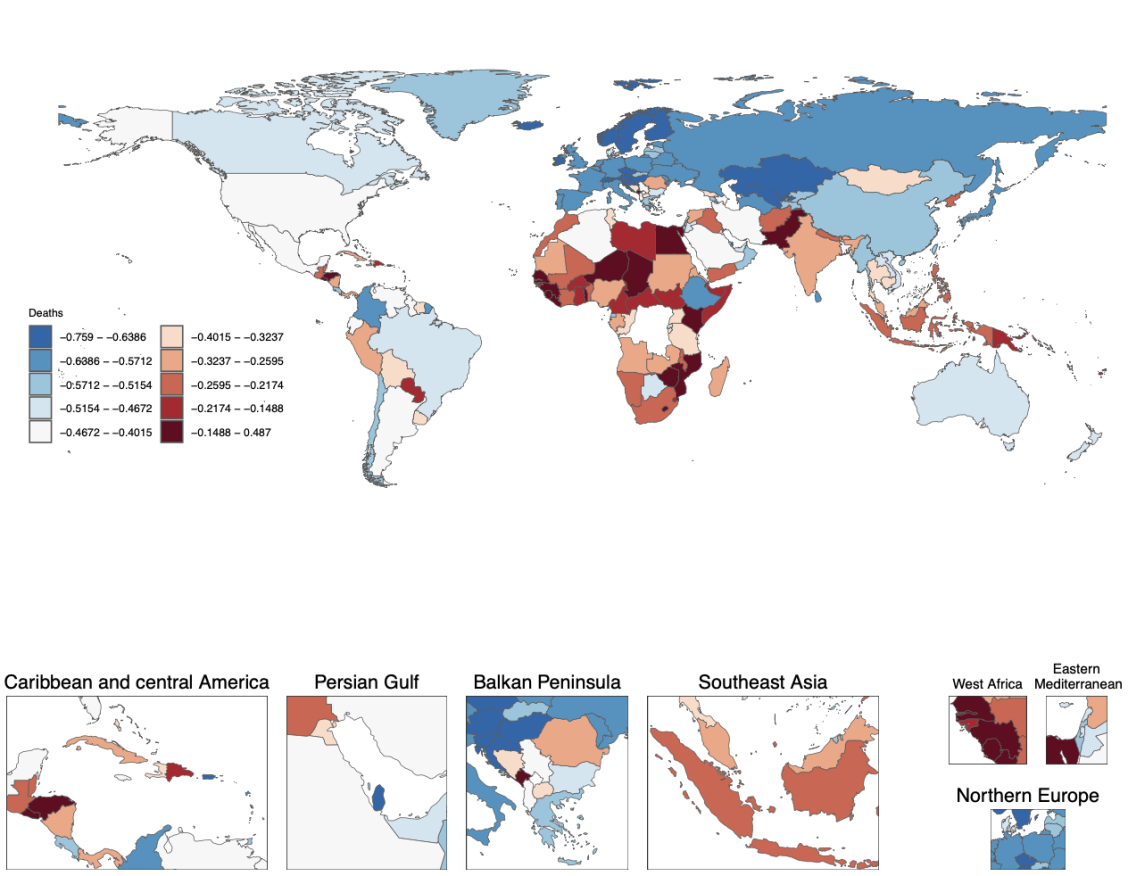


B


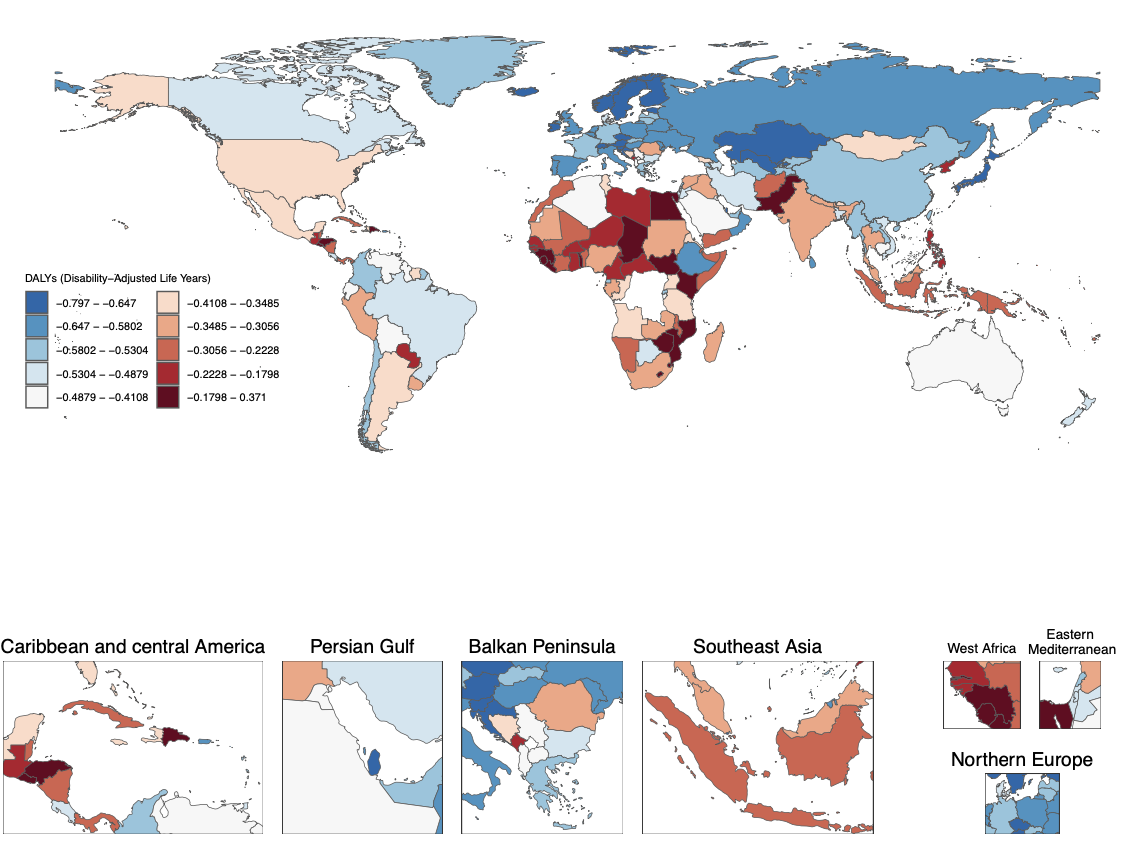


Figure S10. Average annual percentage change of age-standardized death and DALY rates attributable to diet high in sodium for both sexes combined from 1990 to 2021. (A) Deaths. (B) DALYs.

A


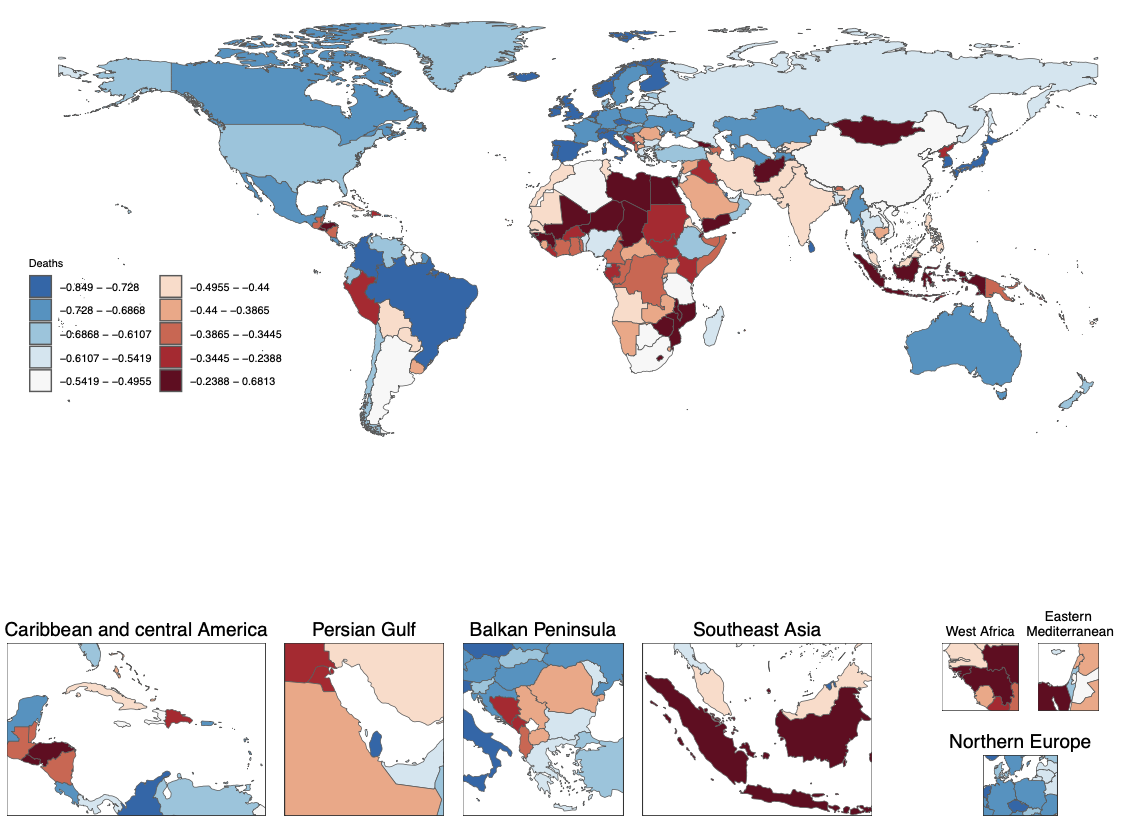


B


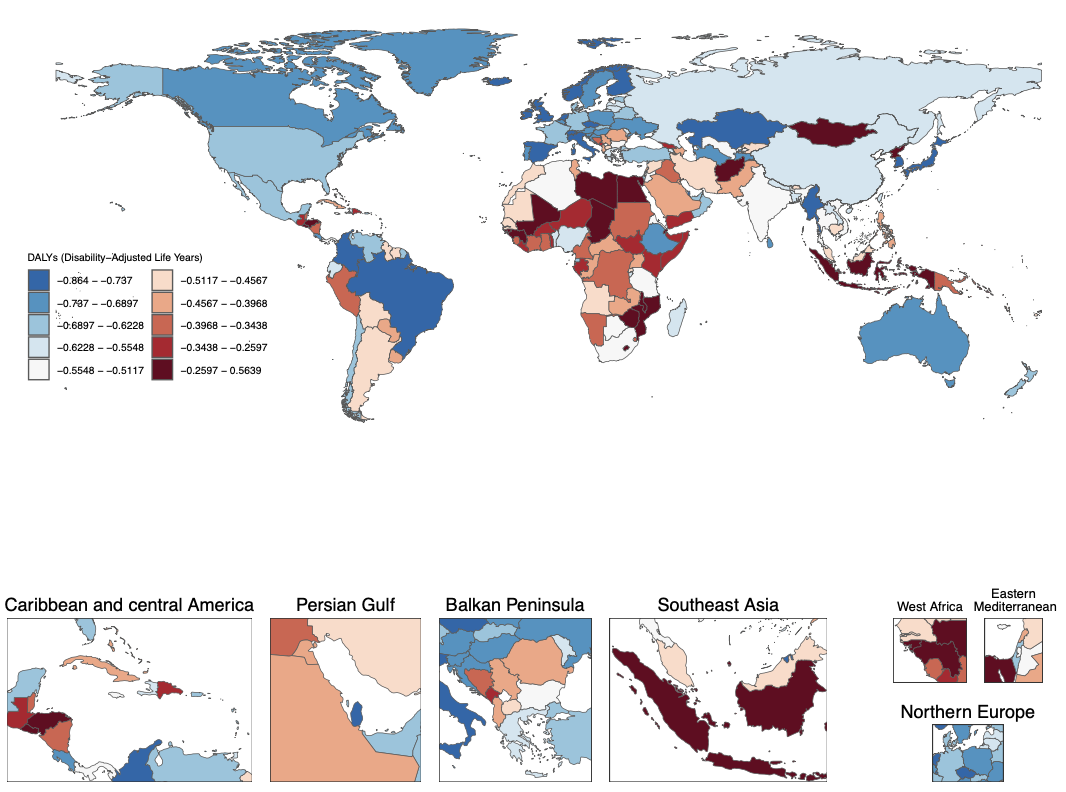


Figure S11. Average annual percentage change of age-standardized death and DALY rates attributable to smoking for both sexes combined from 1990 to 2021. (A) Deaths. (B) DALYs.
