# Supplementary material for: Global stomach cancer burden from high-sodium diet and smoking: 1990–2021 findings & 2040 projections
Source: Front Oncol. 2026 Jun 25;16:1838243. doi: 10.3389/fonc.2026.1838243 (PMC13347218; doi:10.3389/fonc.2026.1838243)
Supplement: Supplementary file 2 [file DataSheet2.docx]

**Table S1. Age-standardized deaths and DALYs attributable to diet high in sodium in 2021 and percentage change from 1990 to 2021, by gender, SDI quintile and 204 countries.**

|  | Deaths | | | |  | DALYs | | | |  |
| --- | --- | --- | --- | --- | --- | --- | --- | --- | --- | --- |
|  | 2021 age-standardized  rate per  100,000 people | Percentage  change in age-standardized rate, 1990–2021 | 2021 age-standardized PAF | Percentage  change in age standardized  PAF, 1990–2021 |  | 2021 age-standardized  rate per  100,000 people | Percentage  change in age-standardized rate, 1990–2021 | 2021 age-standardized PAF | Percentage  change in age standardized  PAF, 1990–2021 |  |
|  |  |  |  |  |  |  |  |  |  |  |
|  |  |  |  |  |  |  |  |  |  |  |
| Global | 0.887(-0.000-4.370) | -0.491(-0.751--0.415) | 7.928(-0.000-40.203) | 0.290(-50.001-3.769) |  | 20.783(-0.000-102.378) | -0.533(-0.828--0.462) | 7.924(-0.000-40.127) | -0.175(-62.214-1.725) |  |
| Sex |  |  |  |  |  |  |  |  |  |  |
| Male | 1.292(-0.000-6.341) | -0.474(-0.864--0.355) | 8.071(-0.000-40.643) | 0.268(-73.552-5.552) |  | 29.900(-0.000-146.648) | -0.519(-0.828--0.414) | 8.074(-0.000-40.598) | 0.031(-63.540-4.063) |  |
| Female | 0.547(0.000-2.795) | -0.526(-0.871--0.458) | 7.653(0.000-39.420) | -0.126(-73.433-3.094) |  | 12.607(0.000-64.624) | -0.561(-0.791--0.495) | 7.615(0.000-38.910) | -0.927(-50.090-1.003) |  |
| Socio-demographic index |  |  |  |  |  |  |  |  |  |  |
| low | 0.599(0.000-3.183) | -0.302(-0.734--0.215) | 7.119(0.000-37.558) | -1.232(-63.794-0.448) |  | 14.711(0.000-78.407) | -0.345(-0.755--0.256) | 7.058(0.000-37.251) | -2.039(-65.846--0.189) |  |
| Low-middle | 0.592(0.000-3.004) | -0.275(-0.729--0.162) | 7.706(0.000-39.671) | -0.938(-63.503-0.668) |  | 14.783(0.000-75.093) | -0.309(-0.791--0.212) | 7.713(0.000-39.563) | -0.713(-67.795-1.762) |  |
| Middle | 1.107(-0.000-5.430) | -0.519(-0.937--0.443) | 8.075(-0.000-40.654) | -1.052(-84.560-0.064) |  | 25.818(-0.000-127.576) | -0.563(-0.943--0.488) | 8.072(-0.000-40.582) | -0.998(-84.244-0.098) |  |
| High-Middle | 1.184(-0.000-5.791) | -0.515(-0.697--0.339) | 7.935(-0.000-40.127) | 1.322(-29.239-21.394) |  | 28.139(-0.000-136.923) | -0.558(-0.789--0.396) | 7.977(-0.000-40.236) | 0.991(-53.597-17.995) |  |
| High | 0.543(0.000-2.733) | -0.561(-0.865--0.526) | 7.932(0.000-40.418) | 1.730(-67.957-7.120) |  | 11.603(0.000-58.092) | -0.612(-0.845--0.586) | 7.929(0.000-40.344) | 1.163(-59.445-4.222) |  |
| Countries |  |  |  |  |  |  |  |  |  |  |
| Afghanistan | 1.587(0.000-10.321) | -0.232(-1.224-2.182) | 4.727(-0.000-28.818) | -6.750(-120.760-237.385) |  | 42.896(-0.000-275.010) | -0.252(-1.330-1.880) | 4.866(-0.000-29.606) | -5.644(-135.637-230.153) |  |
| Albania | 0.875(0.000-4.515) | -0.403(-0.895--0.186) | 8.334(0.000-41.517) | -0.035(-83.359-0.353) |  | 19.050(-0.000-99.745) | -0.430(-0.928--0.229) | 8.307(-0.000-41.409) | 0.103(-87.173-0.548) |  |
| Algeria | 0.182(0.000-1.087) | -0.430(-1.009-1.423) | 4.866(-0.000-29.410) | -2.828(-101.616-381.011) |  | 4.216(-0.000-25.389) | -0.447(-1.006-1.027) | 5.233(-0.000-31.000) | -1.677(-101.051-292.940) |  |
| American Samoa | 1.262(-0.000-6.606) | -0.134(-1.029-3.040) | 7.166(-0.000-37.815) | 3.723(-103.632-343.023) |  | 28.692(-0.000-156.552) | -0.144(-0.829-3.259) | 6.640(-0.000-35.966) | 3.017(-80.101-383.260) |  |
| Andorra | 0.457(0.000-2.660) | -0.486(-0.967-0.939) | 6.710(0.000-36.522) | -0.923(-92.428-269.430) |  | 9.749(0.000-56.367) | -0.507(-1.027-0.900) | 6.911(0.000-37.258) | -0.297(-104.405-243.431) |  |
| Angola | 0.601(0.000-3.349) | -0.319(-0.905-5.695) | 6.861(0.000-37.089) | 8.557(-84.887-1036.266) |  | 14.376(0.000-81.791) | -0.354(-0.944-3.410) | 6.694(0.000-36.524) | 8.392(-90.046-687.653) |  |
| Antigua and Barbuda | 0.760(-0.000-4.014) | -0.429(-1.059--0.107) | 7.574(-0.000-39.342) | -1.593(-110.210-56.314) |  | 16.164(-0.000-85.678) | -0.467(-1.024-0.050) | 7.462(-0.000-38.832) | -1.316(-104.304-88.139) |  |
| Argentina | 0.676(0.000-3.426) | -0.413(-1.169-0.019) | 8.083(0.000-40.909) | 0.012(-129.875-66.638) |  | 15.846(0.000-80.222) | -0.406(-1.226-0.043) | 8.053(0.000-40.748) | 0.091(-140.145-69.244) |  |
| Armenia | 0.841(0.000-4.370) | -0.524(-1.014--0.453) | 7.804(0.000-40.058) | -3.794(-103.042--0.824) |  | 20.070(0.000-104.484) | -0.576(-1.029--0.530) | 7.753(0.000-39.872) | -3.931(-106.558--1.022) |  |
| Australia | 0.234(0.000-1.336) | -0.470(-0.981-0.191) | 6.199(-0.000-35.263) | 2.982(-96.375-136.762) |  | 5.318(-0.000-29.708) | -0.482(-0.962-0.245) | 6.544(-0.000-36.146) | 3.150(-92.077-140.716) |  |
| Austria | 0.371(0.000-1.879) | -0.717(-1.024--0.366) | 8.034(0.000-40.839) | 0.964(-108.840-125.842) |  | 8.037(0.000-40.825) | -0.710(-1.030--0.375) | 7.964(0.000-40.616) | 0.929(-110.259-117.483) |  |
| Azerbaijan | 1.117(-0.000-5.951) | -0.461(-0.952--0.331) | 7.789(-0.000-40.116) | -4.176(-90.998--1.073) |  | 27.105(-0.000-143.446) | -0.515(-1.005--0.387) | 7.735(-0.000-39.842) | -4.242(-100.963--1.097) |  |
| Bahrain | 0.328(0.000-1.995) | -0.550(-1.021-1.489) | 4.839(-0.000-28.549) | -5.446(-104.594-481.597) |  | 6.924(-0.000-42.206) | -0.580(-1.040-1.290) | 5.229(-0.000-30.477) | -3.054(-109.926-392.611) |  |
| Bangladesh | 0.428(0.000-2.197) | -0.432(-1.081-4.723) | 8.006(0.000-40.736) | 6.193(-116.693-884.979) |  | 10.423(0.000-53.926) | -0.488(-0.711-3.913) | 7.958(0.000-40.528) | 6.287(-29.741-882.748) |  |
| Barbados | 0.660(0.000-3.735) | -0.442(-1.016-1.840) | 6.800(-0.000-37.256) | -0.675(-103.676-377.685) |  | 14.465(-0.000-82.287) | -0.456(-1.101-1.095) | 6.817(-0.000-37.435) | -0.414(-119.643-271.239) |  |
| Belarus | 0.844(-0.000-4.511) | -0.609(-1.003-0.091) | 6.821(-0.000-36.253) | -1.061(-100.885-175.325) |  | 22.550(-0.000-118.457) | -0.620(-0.986-0.279) | 6.957(-0.000-36.609) | -0.545(-95.996-207.029) |  |
| Belgium | 0.356(-0.000-1.804) | -0.631(-0.970--0.576) | 7.952(-0.000-40.699) | -0.990(-92.043-16.884) |  | 7.564(-0.000-38.483) | -0.614(-0.987--0.554) | 7.897(-0.000-40.418) | -0.737(-96.682-13.527) |  |
| Belize | 0.701(0.000-3.662) | -0.250(-0.904-0.231) | 7.491(0.000-39.369) | -1.721(-86.801-58.151) |  | 16.511(0.000-86.389) | -0.234(-1.317-0.505) | 7.364(0.000-38.967) | -1.301(-141.508-88.003) |  |
| Benin | 0.731(0.000-3.789) | -0.223(-1.039-0.994) | 7.595(0.000-39.471) | 1.396(-105.219-153.534) |  | 16.665(0.000-86.709) | -0.261(-1.057-0.838) | 7.550(0.000-39.353) | 1.038(-107.757-144.831) |  |
| Bermuda | 0.373(-0.000-2.010) | -0.632(-0.976--0.340) | 7.655(-0.000-39.623) | -0.836(-93.270-76.676) |  | 7.894(-0.000-42.508) | -0.647(-0.982--0.327) | 7.562(-0.000-39.221) | -0.495(-94.826-89.600) |  |
| Bhutan | 0.443(0.000-2.397) | -0.286(-0.657-5.412) | 8.003(0.000-40.821) | 6.018(-41.973-846.164) |  | 10.494(0.000-57.196) | -0.368(-0.640-4.391) | 7.965(0.000-40.602) | 6.204(-12.983-892.721) |  |
| Bolivia | 2.584(0.000-13.232) | -0.381(-1.038--0.159) | 8.034(0.000-40.829) | -0.961(-105.027-7.082) |  | 56.468(0.000-288.198) | -0.422(-0.994--0.195) | 7.980(0.000-40.619) | -0.843(-98.753-7.226) |  |
| Bosnia and Herzegovina | 0.725(-0.000-3.720) | -0.368(-0.659--0.206) | 8.330(-0.000-41.504) | -0.122(-36.809-0.553) |  | 16.633(-0.000-84.942) | -0.410(-0.724--0.251) | 8.303(-0.000-41.390) | -0.111(-46.832-0.307) |  |
| Botswana | 0.359(0.000-2.014) | -0.505(-0.966--0.241) | 6.330(-0.000-35.679) | -7.269(-93.716-31.512) |  | 8.624(0.000-48.601) | -0.530(-0.932--0.208) | 6.548(0.000-36.169) | -6.988(-88.146-53.220) |  |
| Brazil | 0.784(-0.000-4.021) | -0.510(-0.884--0.429) | 7.994(-0.000-40.693) | -1.124(-75.955-14.737) |  | 18.952(-0.000-97.305) | -0.498(-0.820--0.395) | 7.947(-0.000-40.414) | -1.230(-64.342-19.130) |  |
| Brunei | 0.841(0.000-4.305) | -0.570(-0.878--0.464) | 8.300(0.000-41.415) | -0.081(-73.425-2.531) |  | 19.529(0.000-98.367) | -0.582(-0.903--0.478) | 8.274(0.000-41.279) | 0.163(-75.673-2.152) |  |
| Bulgaria | 0.942(-0.000-4.634) | -0.500(-0.832--0.393) | 8.323(-0.000-41.458) | -0.174(-68.854-0.164) |  | 22.765(-0.000-112.125) | -0.493(-0.823--0.387) | 8.288(-0.000-41.307) | -0.194(-67.685-0.221) |  |
| Burkina Faso | 0.872(0.000-4.859) | -0.153(-0.811-2.613) | 7.349(0.000-38.329) | 2.244(-75.419-347.773) |  | 20.057(0.000-112.787) | -0.187(-0.770-3.022) | 7.320(0.000-38.256) | 2.063(-70.890-368.355) |  |
| Burundi | 0.621(0.000-3.180) | -0.401(-0.999--0.263) | 7.896(0.000-40.271) | -3.896(-99.866--1.133) |  | 14.942(0.000-77.393) | -0.446(-1.012--0.314) | 7.665(0.000-39.485) | -5.741(-101.883--1.919) |  |
| Cambodia | 0.863(0.000-4.274) | -0.380(-0.924--0.183) | 8.274(0.000-41.310) | -0.295(-87.798-0.307) |  | 21.059(0.000-104.416) | -0.426(-0.990--0.220) | 8.214(0.000-40.988) | -0.233(-97.905-0.430) |  |
| Cameroon | 0.760(0.000-4.292) | -0.173(-1.621-1.903) | 7.338(0.000-39.231) | 2.041(-168.663-275.268) |  | 17.733(-0.000-100.645) | -0.197(-1.092-2.122) | 7.306(-0.000-39.029) | 1.859(-113.763-289.946) |  |
| Canada | 0.302(0.000-1.555) | -0.499(-1.125-0.401) | 7.795(0.000-40.205) | -0.072(-124.916-169.315) |  | 6.632(0.000-33.546) | -0.507(-1.204-0.448) | 7.866(0.000-40.300) | 0.433(-144.452-194.632) |  |
| Cape Verde | 1.778(0.000-9.626) | -0.231(-1.094-1.815) | 7.355(-0.000-38.719) | 2.861(-110.561-301.288) |  | 37.716(-0.000-205.608) | -0.282(-1.133-2.070) | 7.351(-0.000-38.739) | 2.949(-120.261-392.106) |  |
| Central African Republic | 0.895(0.000-5.124) | -0.190(-0.702-4.983) | 6.794(0.000-36.706) | 8.238(-55.993-699.569) |  | 22.891(0.000-132.242) | -0.202(-0.930-3.487) | 6.655(0.000-36.064) | 8.142(-89.911-538.495) |  |
| Chad | 0.996(0.000-5.479) | 0.159(-0.846-3.717) | 7.363(0.000-38.628) | 2.415(-84.554-305.767) |  | 23.573(0.000-130.088) | 0.131(-0.733-3.982) | 7.329(0.000-38.571) | 2.129(-74.143-333.132) |  |
| Chile | 1.230(0.000-6.180) | -0.560(-1.006--0.322) | 8.132(0.000-41.146) | -0.432(-101.391-45.579) |  | 27.132(0.000-135.975) | -0.571(-1.064--0.361) | 8.112(0.000-41.033) | -0.471(-115.278-46.123) |  |
| China | 1.783(-0.000-8.808) | -0.537(-0.887--0.418) | 8.299(-0.000-41.421) | -0.177(-69.600-0.983) |  | 41.458(-0.000-208.590) | -0.579(-0.844--0.457) | 8.284(-0.000-41.321) | -0.063(-56.602-1.127) |  |
| Colombia | 1.117(-0.000-5.701) | -0.572(-0.814--0.442) | 8.260(-0.000-41.324) | 0.058(-55.238-17.873) |  | 27.158(-0.000-139.115) | -0.553(-0.899--0.410) | 8.217(-0.000-41.098) | 0.060(-75.398-18.408) |  |
| Comoros | 0.553(0.000-2.827) | -0.311(-0.953--0.115) | 7.942(0.000-40.545) | -3.370(-94.340--0.924) |  | 13.106(0.000-68.403) | -0.359(-0.974--0.163) | 7.723(0.000-39.886) | -5.052(-95.923--1.725) |  |
| Congo | 0.608(-0.000-3.392) | -0.355(-1.078-4.273) | 6.842(-0.000-37.296) | 8.913(-115.800-781.985) |  | 14.527(-0.000-82.319) | -0.390(-1.012-2.540) | 6.672(-0.000-36.868) | 8.533(-102.682-498.695) |  |
| Cook Islands | 0.493(0.000-2.483) | -0.468(-1.041--0.308) | 7.972(0.000-40.539) | 1.528(-108.529-15.712) |  | 11.120(0.000-57.420) | -0.462(-1.056--0.282) | 7.614(0.000-39.481) | 3.046(-112.137-28.985) |  |
| Costa Rica | 1.357(-0.000-6.870) | -0.543(-1.043--0.363) | 8.159(-0.000-41.212) | -0.387(-110.061-35.704) |  | 32.066(-0.000-161.832) | -0.525(-0.972--0.330) | 8.126(-0.000-40.975) | -0.510(-94.123-40.197) |  |
| Cote d'Ivoire | 0.259(0.000-1.349) | -0.238(-0.872-1.029) | 7.512(0.000-39.480) | 0.276(-86.104-152.560) |  | 6.168(0.000-32.870) | -0.251(-1.160-0.930) | 7.462(0.000-39.335) | 0.074(-121.752-139.722) |  |
| Croatia | 0.712(-0.000-3.533) | -0.646(-0.901--0.570) | 8.332(-0.000-41.491) | -0.145(-70.399-0.368) |  | 15.638(-0.000-77.577) | -0.654(-0.980--0.581) | 8.306(-0.000-41.375) | -0.186(-94.201-0.192) |  |
| Cuba | 0.415(0.000-2.153) | -0.313(-1.062--0.052) | 7.535(-0.000-39.471) | -1.513(-109.252-29.284) |  | 9.471(-0.000-48.882) | -0.297(-1.055-0.042) | 7.442(-0.000-39.190) | -0.383(-108.508-52.657) |  |
| Cyprus | 0.438(0.000-2.306) | -0.507(-0.751-0.946) | 7.401(0.000-38.851) | -0.111(-42.100-284.916) |  | 8.451(0.000-44.603) | -0.503(-0.774-0.918) | 7.524(0.000-39.152) | 0.901(-54.611-296.047) |  |
| Czech Republic | 0.457(-0.000-2.260) | -0.678(-0.892--0.610) | 8.329(-0.000-41.501) | -0.181(-65.989-0.418) |  | 10.225(-0.000-50.642) | -0.682(-0.926--0.621) | 8.302(-0.000-41.383) | -0.251(-72.860-0.121) |  |
| Democratic Republic of the Congo | 0.458(0.000-2.791) |  | 5.233(0.000-31.241) |  |  | 11.203(0.000-67.873) |  | 5.217(0.000-31.084) |  |  |
| Denmark | 0.350(0.000-1.833) | -0.491(-0.944-0.227) | 7.589(0.000-39.642) | 1.496(-89.760-136.457) |  | 7.710(0.000-40.233) | -0.517(-0.931-0.211) | 7.684(0.000-39.812) | 1.675(-85.381-156.515) |  |
| Djibouti | 0.596(0.000-3.105) | -0.209(-0.894-0.028) | 7.917(0.000-40.262) | -3.856(-88.080--1.203) |  | 13.991(0.000-74.778) | -0.253(-0.905-0.009) | 7.706(0.000-39.408) | -5.679(-90.386--2.014) |  |
| Dominica | 1.510(-0.000-7.980) | -0.318(-1.157--0.064) | 7.504(-0.000-39.104) | -1.539(-123.619-31.529) |  | 33.289(-0.000-175.284) | -0.307(-1.068-0.072) | 7.414(-0.000-38.903) | -0.728(-110.446-52.819) |  |
| Dominican Republic | 0.515(-0.000-2.761) | -0.200(-1.003-0.063) | 7.493(-0.000-39.380) | -1.455(-100.403-16.649) |  | 12.450(-0.000-66.095) | -0.160(-1.026-0.299) | 7.372(-0.000-38.705) | -0.363(-102.844-39.209) |  |
| Ecuador | 1.497(0.000-7.643) | -0.413(-1.017--0.210) | 8.037(0.000-40.723) | -0.985(-103.106-22.948) |  | 33.621(0.000-171.722) | -0.419(-0.877--0.229) | 7.970(0.000-40.460) | -0.972(-78.484-22.378) |  |
| Egypt | 0.361(0.000-2.160) | 0.487(-1.024-5.130) | 5.077(0.000-29.860) | -4.104(-101.332-240.496) |  | 8.869(0.000-53.200) | 0.349(-1.248-3.830) | 5.341(0.000-31.186) | -4.383(-115.894-216.740) |  |
| El Salvador | 1.424(0.000-7.231) | -0.115(-0.955-0.518) | 8.127(0.000-41.052) | -0.011(-94.867-56.770) |  | 34.848(0.000-178.142) | -0.123(-0.882-0.512) | 8.097(0.000-40.823) | 0.356(-87.042-53.175) |  |
| Equatorial Guinea | 0.448(0.000-2.518) | -0.536(-1.098-5.166) | 6.895(0.000-37.886) | 10.100(-121.108-1217.804) |  | 10.348(0.000-59.066) | -0.580(-1.028-2.201) | 6.713(0.000-37.029) | 9.654(-105.367-622.083) |  |
| Eritrea | 0.801(-0.000-4.291) | -0.298(-1.024--0.133) | 7.913(-0.000-40.219) | -3.720(-103.137--1.173) |  | 19.767(-0.000-107.305) | -0.361(-1.019--0.174) | 7.691(-0.000-39.357) | -5.634(-102.578--1.921) |  |
| Estonia | 0.560(0.000-3.142) | -0.613(-0.997-0.212) | 5.667(-0.000-32.519) | 0.507(-99.206-237.683) |  | 13.419(-0.000-74.980) | -0.651(-0.987-0.222) | 5.820(-0.000-33.163) | 0.527(-96.536-264.167) |  |
| Ethiopia | 0.525(-0.000-2.770) | -0.597(-0.994--0.518) | 7.869(-0.000-40.058) | -3.726(-98.863--0.599) |  | 12.630(-0.000-67.111) | -0.644(-0.993--0.570) | 7.638(-0.000-39.140) | -5.485(-98.148--1.748) |  |
| Federated States of Micronesia | 1.354(0.000-6.904) | -0.245(-0.950-0.044) | 7.875(0.000-40.051) | 2.394(-93.278-17.272) |  | 33.304(0.000-175.381) | -0.246(-0.966-0.158) | 7.484(0.000-38.545) | 4.109(-95.668-29.585) |  |
| Fiji | 0.653(0.000-3.466) | -0.197(-1.052-0.153) | 7.969(0.000-40.550) | 2.945(-107.432-19.909) |  | 14.674(0.000-79.242) | -0.231(-1.062-0.198) | 7.609(0.000-39.371) | 5.241(-109.125-31.668) |  |
| Finland | 0.307(-0.000-1.598) | -0.679(-1.034--0.269) | 7.347(-0.000-38.750) | 0.492(-110.216-125.751) |  | 6.678(-0.000-34.869) | -0.696(-1.045--0.343) | 7.484(-0.000-39.132) | 0.707(-114.082-119.466) |  |
| France | 0.331(-0.000-1.732) | -0.572(-0.992-1.332) | 7.357(-0.000-38.887) | 2.815(-97.902-454.870) |  | 7.473(-0.000-39.319) | -0.537(-0.933-2.055) | 7.384(-0.000-38.920) | 4.443(-82.628-587.605) |  |
| Gabon | 0.546(0.000-3.036) | -0.310(-0.975-5.814) | 6.902(0.000-37.191) | 8.428(-94.770-936.247) |  | 12.747(0.000-72.332) | -0.336(-0.891-3.287) | 6.733(0.000-36.558) | 8.322(-79.330-613.625) |  |
| Georgia | 0.924(0.000-4.719) | -0.341(-1.010--0.268) | 7.791(0.000-40.115) | -3.981(-101.481--0.685) |  | 22.660(0.000-117.214) | -0.405(-0.965--0.335) | 7.755(0.000-39.962) | -3.825(-94.576--0.512) |  |
| Germany | 0.437(0.000-2.195) | -0.593(-0.717-0.162) | 7.534(0.000-39.554) | 2.622(-27.139-191.119) |  | 10.094(0.000-50.470) | -0.578(-0.812-0.153) | 7.686(0.000-39.793) | 2.756(-54.915-167.819) |  |
| Ghana | 0.614(0.000-3.132) | -0.163(-1.566-5.325) | 7.914(0.000-40.664) | 4.648(-181.580-702.982) |  | 13.806(0.000-71.197) | -0.210(-1.229-4.904) | 7.882(0.000-40.530) | 4.808(-134.991-654.063) |  |
| Greece | 0.551(0.000-2.867) | -0.547(-0.724-0.843) | 7.494(0.000-39.672) | 1.322(-39.309-314.499) |  | 12.219(0.000-62.730) | -0.531(-0.677-1.043) | 7.606(0.000-39.882) | 1.856(-29.748-324.029) |  |
| Greenland | 0.679(0.000-3.532) | -0.559(-1.181-1.055) | 7.812(0.000-40.293) | 2.400(-144.386-347.562) |  | 17.004(-0.000-87.850) | -0.575(-1.098-1.043) | 7.885(-0.000-40.327) | 2.233(-122.857-388.883) |  |
| Grenada | 0.639(-0.000-3.348) | -0.419(-1.013--0.127) | 7.459(-0.000-39.211) | -1.099(-102.017-45.981) |  | 14.340(-0.000-75.828) | -0.458(-1.067-0.099) | 7.370(-0.000-38.729) | -0.053(-110.628-95.136) |  |
| Guam | 0.381(-0.000-1.963) | -0.487(-0.955--0.408) | 7.736(-0.000-39.597) | -2.277(-92.327-1.333) |  | 10.639(-0.000-56.352) | -0.321(-0.904--0.210) | 7.342(-0.000-38.409) | -1.709(-85.007-3.762) |  |
| Guatemala | 1.977(0.000-10.245) | -0.253(-0.894-0.103) | 8.095(0.000-40.985) | -0.182(-86.504-46.865) |  | 46.771(0.000-242.029) | -0.206(-1.115-0.107) | 8.046(0.000-40.671) | 0.137(-113.594-36.640) |  |
| Guinea | 0.714(0.000-3.807) | -0.102(-1.098-3.044) | 7.337(0.000-38.965) | 2.112(-112.718-310.101) |  | 17.441(0.000-94.453) | -0.116(-1.110-2.735) | 7.282(0.000-38.655) | 1.830(-113.871-311.713) |  |
| Guinea-Bissau | 1.181(0.000-6.361) | -0.167(-1.018-2.294) | 7.339(-0.000-38.965) | 1.715(-102.169-286.530) |  | 28.791(-0.000-155.739) | -0.212(-0.908-2.119) | 7.298(-0.000-38.674) | 1.518(-87.849-312.613) |  |
| Guyana | 0.594(-0.000-3.264) | -0.440(-1.046--0.180) | 7.431(-0.000-39.029) | -2.002(-108.899-30.990) |  | 14.737(-0.000-82.089) | -0.419(-0.912-0.063) | 7.320(-0.000-38.557) | -1.707(-84.601-75.956) |  |
| Haiti | 1.381(-0.000-7.329) | -0.360(-0.983-0.112) | 7.440(-0.000-39.166) | -1.230(-97.624-78.092) |  | 32.741(-0.000-174.866) | -0.380(-0.959-0.180) | 7.305(-0.000-38.481) | -0.816(-92.673-63.514) |  |
| Honduras | 1.679(-0.000-8.554) | 0.171(-1.097-0.562) | 8.114(-0.000-40.798) | -0.171(-107.517-26.002) |  | 37.281(0.000-189.210) | 0.055(-1.064-0.448) | 8.096(0.000-40.657) | 0.324(-105.480-23.201) |  |
| Hungary | 0.590(-0.000-2.897) | -0.651(-0.900--0.585) | 8.340(-0.000-41.519) | -0.046(-70.917-0.197) |  | 14.024(-0.000-68.817) | -0.645(-0.897--0.576) | 8.318(-0.000-41.415) | -0.013(-67.100-0.280) |  |
| Iceland | 0.331(0.000-1.738) | -0.681(-0.925--0.307) | 7.622(0.000-39.604) | 0.061(-76.083-100.544) |  | 6.915(0.000-36.080) | -0.699(-0.899--0.307) | 7.704(0.000-39.899) | 0.676(-69.385-117.270) |  |
| India | 0.455(0.000-2.307) | -0.279(-0.403-0.261) | 7.824(0.000-39.930) | -0.948(-12.199-53.886) |  | 11.762(0.000-59.398) | -0.325(-0.438-0.174) | 7.918(0.000-40.143) | -0.086(-6.997-57.630) |  |
| Indonesia | 0.615(0.000-3.112) | -0.227(-0.873-0.001) | 8.252(0.000-41.277) | -0.389(-85.946-1.301) |  | 14.581(0.000-73.624) | -0.300(-0.949--0.099) | 8.192(0.000-41.033) | -0.238(-93.768-1.181) |  |
| Iran | 0.664(0.000-4.044) | -0.461(-0.863-1.198) | 5.008(0.000-29.733) | -2.910(-75.631-281.599) |  | 15.548(0.000-92.327) | -0.489(-0.848-0.753) | 5.313(0.000-31.163) | -2.105(-70.694-242.605) |  |
| Iraq | 0.263(0.000-1.594) | -0.258(-0.976-1.549) | 5.040(-0.000-30.080) | -5.640(-96.829-208.890) |  | 6.617(-0.000-39.906) | -0.331(-0.953-1.291) | 5.285(-0.000-31.239) | -3.584(-92.551-228.639) |  |
| Ireland | 0.311(0.000-1.693) | -0.659(-1.018--0.084) | 6.721(-0.000-36.330) | -0.109(-105.442-154.561) |  | 6.532(-0.000-35.121) | -0.670(-1.053--0.043) | 6.905(-0.000-37.066) | 0.299(-116.523-181.231) |  |
| Israel | 0.386(0.000-1.991) | -0.527(-0.927-0.626) | 7.478(0.000-39.411) | 0.968(-84.304-256.389) |  | 8.234(0.000-42.159) | -0.525(-0.937-0.608) | 7.588(0.000-39.591) | 1.795(-86.090-238.487) |  |
| Italy | 0.563(-0.000-2.900) | -0.632(-1.030--0.577) | 7.718(-0.000-39.846) | -1.158(-108.221-13.709) |  | 11.954(-0.000-60.433) | -0.643(-1.029--0.594) | 7.853(-0.000-40.246) | -0.713(-108.299-11.681) |  |
| Jamaica | 0.690(0.000-3.743) | -0.419(-0.997--0.073) | 7.554(0.000-39.548) | -1.613(-99.372-48.033) |  | 15.729(0.000-85.801) | -0.395(-0.989-0.065) | 7.451(0.000-39.062) | -1.263(-98.625-79.111) |  |
| Japan | 1.092(0.000-5.463) | -0.612(-0.936--0.598) | 8.244(0.000-41.269) | -1.030(-83.042-0.087) |  | 22.258(0.000-111.560) | -0.661(-0.946--0.651) | 8.220(0.000-41.169) | -1.068(-84.318--0.002) |  |
| Jordan | 0.194(0.000-1.167) | -0.488(-0.981-1.606) | 4.991(-0.000-29.869) | -3.089(-96.500-386.154) |  | 4.687(-0.000-27.713) | -0.526(-0.983-1.838) | 5.341(-0.000-31.155) | -0.609(-96.242-486.919) |  |
| Kazakhstan | 0.837(0.000-4.357) | -0.694(-0.982--0.652) | 7.764(0.000-39.987) | -4.287(-94.598--1.376) |  | 21.605(0.000-112.227) | -0.707(-0.978--0.666) | 7.735(0.000-39.891) | -4.120(-93.650--1.209) |  |
| Kenya | 0.476(-0.000-2.726) | -0.077(-0.827-0.317) | 5.778(-0.000-32.289) | -11.644(-85.984-22.454) |  | 11.205(-0.000-64.411) | -0.120(-0.738-0.197) | 5.595(-0.000-31.551) | -12.057(-75.934-10.962) |  |
| Kiribati | 1.729(0.000-8.992) | -0.086(-0.943-0.279) | 7.850(0.000-40.093) | 2.525(-94.318-15.932) |  | 42.637(-0.000-225.765) | -0.102(-0.956-0.312) | 7.428(-0.000-38.565) | 4.114(-94.477-29.017) |  |
| Kuwait | 0.184(0.000-1.001) | -0.365(-0.948-0.447) | 7.125(0.000-37.567) | 2.531(-89.422-138.115) |  | 3.984(0.000-21.485) | -0.430(-0.993-0.174) | 7.265(0.000-38.135) | 2.259(-99.189-103.851) |  |
| Kyrgyzstan | 1.189(0.000-6.195) | -0.534(-0.921--0.459) | 7.802(0.000-40.279) | -3.975(-83.556--0.789) |  | 31.595(0.000-165.389) | -0.566(-0.944--0.496) | 7.787(0.000-40.159) | -3.676(-87.900--0.583) |  |
| Laos | 0.708(0.000-3.569) | -0.510(-0.944--0.334) | 8.269(0.000-41.360) | -0.374(-86.492-0.411) |  | 17.382(0.000-88.007) | -0.554(-0.927--0.377) | 8.201(0.000-41.014) | -0.469(-84.381-0.371) |  |
| Latvia | 0.777(0.000-4.050) | -0.551(-0.931--0.050) | 7.187(0.000-38.701) | 1.386(-84.170-113.635) |  | 19.651(-0.000-100.561) | -0.576(-0.997--0.144) | 7.301(-0.000-38.964) | 1.672(-99.330-102.655) |  |
| Lebanon | 0.265(0.000-1.586) | -0.507(-0.990-0.814) | 4.894(-0.000-28.593) | -3.067(-98.023-280.364) |  | 6.222(-0.000-36.594) | -0.546(-0.941-1.198) | 5.206(-0.000-30.251) | -1.819(-86.324-402.579) |  |
| Lesotho | 0.814(0.000-4.672) | 0.272(-0.888-1.277) | 6.490(-0.000-35.998) | -4.874(-89.203-76.512) |  | 21.790(0.000-122.946) | 0.371(-0.838-1.675) | 6.703(0.000-36.890) | -4.524(-87.146-94.053) |  |
| Liberia | 0.745(0.000-4.107) | -0.097(-0.929-3.548) | 7.338(0.000-39.089) | 1.954(-92.755-382.394) |  | 17.252(0.000-95.142) | -0.118(-0.909-2.771) | 7.298(0.000-38.837) | 1.621(-89.224-332.129) |  |
| Libya | 0.333(0.000-2.064) | -0.193(-1.041-2.211) | 5.052(-0.000-30.486) | -3.209(-104.636-298.807) |  | 8.461(-0.000-52.157) | -0.197(-1.047-2.024) | 5.295(-0.000-31.732) | -2.654(-104.756-252.556) |  |
| Lithuania | 0.813(0.000-4.170) | -0.558(-0.997-0.219) | 7.582(-0.000-39.699) | 0.173(-99.405-175.435) |  | 20.245(-0.000-104.251) | -0.577(-0.955-0.150) | 7.557(-0.000-39.566) | 0.321(-89.618-175.366) |  |
| Luxembourg | 0.332(0.000-1.742) | -0.665(-1.154--0.238) | 7.447(0.000-39.304) | 0.549(-140.344-123.357) |  | 6.678(0.000-34.694) | -0.698(-1.119--0.331) | 7.563(0.000-39.599) | 1.083(-135.215-121.627) |  |
| Macedonia | 1.167(-0.000-5.721) | -0.363(-0.678--0.185) | 8.333(-0.000-41.497) | -0.103(-47.042-0.535) |  | 24.907(-0.000-122.516) | -0.430(-0.752--0.251) | 8.308(-0.000-41.385) | -0.094(-54.105-0.623) |  |
| Madagascar | 0.507(0.000-2.599) | -0.307(-0.968--0.139) | 7.913(0.000-40.304) | -3.642(-95.205--1.064) |  | 12.501(0.000-64.659) | -0.335(-0.898--0.170) | 7.685(0.000-39.466) | -5.333(-85.307--1.621) |  |
| Malawi | 0.254(0.000-1.294) | -0.234(-0.968--0.058) | 7.873(0.000-40.292) | -4.218(-95.677--1.076) |  | 6.208(0.000-31.889) | -0.256(-0.970--0.052) | 7.637(0.000-39.447) | -6.105(-95.799--2.006) |  |
| Malaysia | 0.526(0.000-2.669) | -0.291(-0.914--0.185) | 8.289(0.000-41.406) | -0.380(-87.492-0.397) |  | 12.290(0.000-62.325) | -0.325(-0.930--0.225) | 8.261(0.000-41.259) | -0.305(-89.568-0.407) |  |
| Maldives | 0.233(0.000-1.181) | -0.709(-1.001--0.619) | 8.281(0.000-41.403) | -0.360(-100.199-0.348) |  | 5.063(0.000-25.887) | -0.753(-0.998--0.671) | 8.231(0.000-41.185) | -0.378(-99.349-0.318) |  |
| Mali | 1.245(0.000-6.522) | -0.232(-1.062-2.025) | 7.332(0.000-38.766) | 2.005(-107.655-310.986) |  | 30.416(0.000-159.737) | -0.259(-0.990-1.977) | 7.280(0.000-38.343) | 1.794(-98.554-318.604) |  |
| Malta | 0.365(0.000-1.865) | -0.648(-0.966--0.020) | 7.776(0.000-40.219) | 1.554(-89.738-171.861) |  | 7.881(0.000-40.120) | -0.650(-1.115--0.099) | 7.905(0.000-40.489) | 1.624(-134.576-154.358) |  |
| Marshall Islands | 1.405(0.000-7.274) | -0.228(-0.948-0.023) | 7.859(0.000-40.065) | 1.895(-93.114-17.446) |  | 34.902(0.000-184.280) | -0.220(-1.049-0.096) | 7.456(0.000-38.706) | 3.335(-104.767-24.374) |  |
| Mauritania | 0.665(0.000-3.580) | -0.263(-0.949-1.675) | 7.335(0.000-39.034) | 1.987(-91.438-296.254) |  | 14.695(0.000-79.355) | -0.319(-0.909-1.499) | 7.308(0.000-38.939) | 1.689(-86.836-241.493) |  |
| Mauritius | 0.824(0.000-4.078) | -0.380(-0.944--0.330) | 8.274(0.000-41.319) | -0.457(-90.523-0.254) |  | 20.675(0.000-102.347) | -0.369(-0.942--0.312) | 8.217(0.000-41.032) | -0.647(-91.037-0.090) |  |
| Mexico | 0.712(-0.000-3.701) | -0.449(-1.026--0.023) | 7.741(-0.000-39.712) | -0.183(-104.389-75.491) |  | 17.135(-0.000-89.360) | -0.384(-0.973-0.213) | 7.711(-0.000-39.494) | -0.155(-95.677-92.505) |  |
| Moldova | 0.580(-0.000-3.154) | -0.588(-0.965-0.041) | 6.907(-0.000-36.905) | -0.958(-90.751-143.857) |  | 15.629(-0.000-84.417) | -0.594(-0.992--0.007) | 7.009(-0.000-37.282) | -0.394(-98.048-154.733) |  |
| Monaco | 0.531(-0.000-2.811) | -0.402(-1.087-1.112) | 7.472(-0.000-39.158) | 0.645(-115.947-246.444) |  | 11.579(-0.000-60.307) | -0.412(-1.034-1.828) | 7.582(-0.000-39.346) | 1.242(-107.426-310.761) |  |
| Mongolia | 2.956(0.000-15.681) | -0.346(-1.011--0.185) | 7.824(0.000-40.139) | -3.509(-101.937--1.129) |  | 73.195(0.000-380.582) | -0.372(-1.013--0.211) | 7.785(0.000-39.925) | -3.398(-102.037--0.862) |  |
| Montenegro | 0.656(-0.000-3.283) | -0.091(-0.731-0.117) | 8.334(-0.000-41.518) | -0.043(-69.497-0.253) |  | 14.080(-0.000-70.682) | -0.197(-0.692-0.000) | 8.309(-0.000-41.414) | 0.044(-59.568-0.366) |  |
| Morocco | 0.127(0.000-0.785) | -0.250(-0.998-1.054) | 5.116(-0.000-30.927) | -2.990(-99.761-144.743) |  | 3.139(-0.000-18.980) | -0.287(-0.994-1.093) | 5.292(-0.000-31.744) | -2.765(-99.186-180.503) |  |
| Mozambique | 0.639(0.000-3.342) | -0.086(-1.000-0.137) | 7.951(0.000-40.643) | -3.654(-100.039--1.251) |  | 14.208(-0.000-74.860) | -0.092(-1.026-0.123) | 7.755(-0.000-40.057) | -5.470(-102.450--1.964) |  |
| Myanmar | 0.611(0.000-3.024) | -0.533(-1.038--0.354) | 8.267(0.000-41.262) | -0.252(-108.366-0.394) |  | 14.979(0.000-75.450) | -0.575(-0.978--0.398) | 8.196(0.000-40.938) | -0.158(-95.641-0.661) |  |
| Namibia | 0.175(0.000-0.975) | -0.256(-0.946-0.208) | 6.401(0.000-35.916) | -6.840(-91.530-48.018) |  | 4.583(-0.000-25.001) | -0.263(-0.929-0.183) | 6.643(-0.000-36.496) | -6.092(-87.852-44.693) |  |
| Nauru | 1.639(-0.000-8.289) | -0.207(-1.020-0.065) | 7.841(-0.000-39.904) | 2.211(-102.580-11.115) |  | 41.586(-0.000-217.062) | -0.191(-0.856-0.149) | 7.447(-0.000-38.345) | 3.860(-81.230-21.494) |  |
| Nepal | 0.495(0.000-2.505) | -0.246(-1.692-5.765) | 8.006(0.000-40.496) | 6.120(-190.265-890.629) |  | 12.081(-0.000-61.325) | -0.311(-0.894-5.682) | 7.967(-0.000-40.338) | 6.437(-72.227-941.158) |  |
| Netherlands | 0.340(0.000-1.808) | -0.618(-1.054-0.534) | 7.316(0.000-38.653) | 5.995(-116.046-328.494) |  | 7.054(-0.000-36.753) | -0.635(-1.010-0.193) | 7.413(-0.000-38.852) | 6.063(-103.649-260.149) |  |
| New Zealand | 0.339(0.000-1.836) | -0.515(-1.106-0.063) | 7.383(0.000-39.059) | 0.818(-122.266-118.468) |  | 7.793(0.000-41.759) | -0.521(-1.179-0.079) | 7.546(0.000-39.403) | 1.091(-137.293-125.463) |  |
| Nicaragua | 0.789(0.000-3.991) | -0.307(-0.669-0.232) | 8.145(0.000-41.092) | -0.069(-50.684-56.499) |  | 18.947(0.000-96.482) | -0.305(-0.676-0.135) | 8.123(0.000-40.905) | 0.251(-51.249-49.430) |  |
| Niger | 0.848(0.000-4.685) | -0.125(-0.860-2.538) | 7.337(0.000-38.946) | 1.962(-83.557-297.871) |  | 19.281(0.000-105.245) | -0.181(-0.836-3.022) | 7.310(0.000-38.841) | 1.757(-79.609-372.662) |  |
| Nigeria | 0.180(0.000-0.971) | -0.296(-0.716-0.586) | 6.848(0.000-36.730) | 0.048(-59.369-115.313) |  | 4.108(0.000-22.854) | -0.342(-0.766-0.432) | 6.791(0.000-36.567) | -0.452(-63.862-104.526) |  |
| Niue | 0.919(0.000-4.571) | -0.205(-0.879-0.039) | 7.949(0.000-40.465) | 1.982(-82.702-14.933) |  | 20.960(0.000-106.925) | -0.212(-0.854-0.089) | 7.557(0.000-39.216) | 3.336(-80.343-27.324) |  |
| North Korea | 1.795(-0.000-9.257) | -0.220(-0.763-0.050) | 8.315(-0.000-41.412) | -0.113(-73.134-0.868) |  | 48.474(-0.000-249.150) | -0.206(-0.679-0.084) | 8.280(-0.000-41.220) | -0.091(-64.642-0.585) |  |
| Northern Mariana Islands | 1.096(0.000-5.548) | -0.203(-1.092-0.040) | 7.994(0.000-40.555) | 2.044(-113.645-14.688) |  | 24.143(0.000-123.292) | -0.209(-1.087-0.093) | 7.658(0.000-39.423) | 4.110(-113.575-29.423) |  |
| Norway | 0.263(0.000-1.409) | -0.659(-1.042--0.409) | 6.918(0.000-37.251) | -1.018(-112.225-76.001) |  | 5.449(0.000-28.975) | -0.683(-1.045--0.474) | 7.067(0.000-37.909) | -0.619(-114.324-66.634) |  |
| Oman | 0.278(0.000-1.663) | -0.543(-1.000-0.330) | 5.177(-0.000-31.130) | -5.837(-100.080-139.608) |  | 6.409(-0.000-37.746) | -0.590(-1.002-0.218) | 5.492(-0.000-32.300) | -3.546(-100.400-152.255) |  |
| Pakistan | 0.381(-0.000-1.885) | -0.071(-0.706-6.440) | 7.935(-0.000-40.616) | 6.143(-56.149-730.064) |  | 9.514(-0.000-46.806) | -0.073(-0.368-6.655) | 7.876(-0.000-40.354) | 6.266(-13.791-744.263) |  |
| Palau | 1.491(0.000-7.569) | -0.245(-0.987--0.016) | 7.861(0.000-39.776) | 1.159(-98.399-11.972) |  | 34.998(0.000-183.433) | -0.234(-1.238-0.015) | 7.400(0.000-38.065) | 1.945(-129.844-16.714) |  |
| Palestine | 0.314(0.000-1.872) | -0.486(-1.008-1.527) | 4.915(-0.000-29.723) | -3.091(-101.516-436.316) |  | 7.367(-0.000-43.417) | -0.491(-1.085-1.268) | 5.274(-0.000-31.502) | -1.153(-115.421-366.334) |  |
| Panama | 0.812(0.000-4.313) | -0.306(-1.087--0.029) | 8.153(0.000-41.063) | -0.269(-112.345-30.764) |  | 18.865(0.000-99.692) | -0.291(-1.038--0.005) | 8.108(0.000-40.849) | -0.269(-105.722-26.285) |  |
| Papua New Guinea | 1.127(0.000-5.959) | -0.215(-0.954-0.137) | 7.947(0.000-40.331) | 2.450(-93.440-21.010) |  | 26.739(0.000-145.562) | -0.229(-0.938-0.173) | 7.579(0.000-39.071) | 4.126(-90.381-34.965) |  |
| Paraguay | 0.703(0.000-3.633) | -0.174(-1.089-0.208) | 8.085(0.000-41.059) | -0.603(-109.854-44.432) |  | 16.131(0.000-83.901) | -0.182(-0.917-0.217) | 8.045(0.000-40.853) | -0.490(-89.480-50.733) |  |
| Peru | 1.596(0.000-8.332) | -0.316(-0.900--0.015) | 8.040(0.000-40.866) | -0.854(-80.739-22.790) |  | 36.039(0.000-187.320) | -0.343(-0.890--0.019) | 7.974(0.000-40.624) | -0.679(-84.139-29.741) |  |
| Philippines | 0.363(0.000-1.857) | -0.222(-0.810--0.039) | 8.242(0.000-41.202) | -0.241(-75.137-1.744) |  | 9.206(0.000-47.207) | -0.221(-0.853--0.066) | 8.180(0.000-40.869) | 0.009(-80.658-1.150) |  |
| Poland | 0.698(0.000-3.457) | -0.587(-0.744--0.387) | 8.256(0.000-41.239) | 0.879(-37.013-45.805) |  | 16.245(0.000-80.577) | -0.595(-0.946--0.335) | 8.220(0.000-41.094) | 1.190(-86.534-63.659) |  |
| Portugal | 0.784(-0.000-4.147) | -0.601(-0.676-1.585) | 7.286(-0.000-38.516) | 12.160(-4.486-611.012) |  | 18.459(-0.000-94.948) | -0.607(-0.812-1.179) | 7.596(-0.000-39.455) | 9.594(-44.652-486.824) |  |
| Puerto Rico | 0.321(-0.000-1.688) | -0.655(-1.079--0.562) | 7.542(-0.000-39.348) | -2.245(-124.753-16.366) |  | 7.390(-0.000-39.458) | -0.629(-1.053--0.492) | 7.440(-0.000-38.958) | -1.511(-114.033-26.838) |  |
| Qatar | 0.289(0.000-1.752) | -0.659(-0.981-0.788) | 4.957(-0.000-29.503) | -5.908(-94.797-375.706) |  | 6.168(-0.000-36.753) | -0.672(-0.997-0.430) | 5.356(-0.000-31.131) | -4.003(-99.263-319.666) |  |
| Romania | 0.867(0.000-4.332) | -0.310(-0.927--0.191) | 8.332(0.000-41.504) | -0.058(-89.616-0.291) |  | 21.421(0.000-106.860) | -0.346(-0.980--0.243) | 8.311(0.000-41.413) | 0.061(-96.557-0.389) |  |
| Russian Federation | 1.018(0.000-5.154) | -0.589(-1.069--0.488) | 7.842(0.000-40.268) | -0.083(-116.582-25.896) |  | 25.259(0.000-127.115) | -0.625(-1.002--0.532) | 7.940(0.000-40.500) | -0.019(-100.273-26.679) |  |
| Rwanda | 0.545(0.000-2.814) | -0.507(-1.011--0.359) | 7.942(0.000-40.625) | -3.265(-102.125--0.888) |  | 12.826(0.000-66.626) | -0.562(-0.995--0.422) | 7.731(0.000-39.940) | -4.793(-98.955--1.478) |  |
| Saint Kitts and Nevis | 0.692(-0.000-3.840) | -0.472(-1.001--0.223) | 7.558(-0.000-39.299) | -0.760(-100.350-43.802) |  | 14.902(-0.000-82.489) | -0.507(-0.969--0.125) | 7.476(-0.000-39.174) | 0.069(-93.796-80.150) |  |
| Saint Lucia | 0.825(-0.000-4.550) | -0.512(-1.005--0.324) | 7.563(-0.000-39.582) | -1.361(-101.211-32.303) |  | 18.382(-0.000-102.011) | -0.510(-1.000--0.286) | 7.447(-0.000-39.072) | -0.691(-99.346-42.382) |  |
| Saint Vincent and the Grenadines | 0.791(-0.000-4.214) | -0.424(-1.010--0.034) | 7.566(-0.000-39.478) | -0.736(-101.820-74.116) |  | 18.104(-0.000-96.884) | -0.423(-1.041-0.133) | 7.440(-0.000-39.043) | -0.281(-107.557-96.623) |  |
| Samoa | 0.650(0.000-3.766) | -0.136(-4.832-7.388) | 6.192(0.000-34.870) | 7.815(-571.403-933.325) |  | 14.279(0.000-85.359) | -0.131(-5.926-8.449) | 5.464(0.000-32.021) | 6.638(-783.367-1020.240) |  |
| San Marino | 0.708(0.000-3.937) | -0.649(-0.906-0.113) | 7.469(0.000-38.801) | 0.214(-62.302-202.869) |  | 14.903(0.000-83.829) | -0.644(-0.887-0.093) | 7.567(0.000-39.104) | 0.579(-59.987-180.080) |  |
| Sao Tome and Principe | 1.124(0.000-5.836) | -0.083(-1.035-3.544) | 7.341(0.000-38.732) | 2.391(-103.915-420.258) |  | 24.091(0.000-124.388) | -0.113(-1.044-3.763) | 7.334(0.000-38.688) | 2.011(-104.728-451.578) |  |
| Saudi Arabia | 0.188(0.000-1.109) | -0.458(-1.032-1.517) | 5.204(-0.000-30.914) | -2.791(-106.222-368.127) |  | 4.696(-0.000-27.443) | -0.459(-1.048-1.382) | 5.470(-0.000-31.948) | -2.320(-109.706-375.655) |  |
| Senegal | 0.783(0.000-4.272) | -0.136(-1.098-2.129) | 7.337(0.000-38.734) | 2.050(-110.457-256.308) |  | 17.811(0.000-98.072) | -0.185(-1.000-2.314) | 7.304(0.000-38.640) | 1.848(-100.128-320.025) |  |
| Serbia | 0.646(0.000-3.181) | -0.444(-0.830--0.291) | 8.333(0.000-41.534) | -0.098(-72.521-0.252) |  | 15.002(-0.000-74.134) | -0.444(-0.811--0.276) | 8.316(-0.000-41.464) | 0.019(-65.576-0.460) |  |
| Seychelles | 0.482(0.000-2.418) | -0.446(-0.951--0.353) | 7.997(0.000-40.610) | -0.996(-91.747-0.452) |  | 11.354(-0.000-57.089) | -0.487(-0.958--0.394) | 7.656(-0.000-39.559) | -1.819(-92.289-0.685) |  |
| Sierra Leone | 0.768(0.000-4.238) | -0.084(-0.977-2.356) | 7.345(-0.000-38.883) | 2.028(-97.417-287.419) |  | 17.950(-0.000-99.255) | -0.096(-0.914-2.771) | 7.308(-0.000-38.683) | 1.593(-89.801-337.103) |  |
| Singapore | 0.376(0.000-1.868) | -0.726(-0.914--0.697) | 8.294(0.000-41.407) | -0.313(-68.369-2.062) |  | 7.616(0.000-37.782) | -0.763(-0.901--0.732) | 8.250(0.000-41.167) | -0.565(-58.533-5.297) |  |
| Slovakia | 0.684(0.000-3.444) | -0.545(-0.972--0.443) | 8.329(0.000-41.508) | -0.176(-93.058-0.228) |  | 15.655(0.000-78.594) | -0.571(-0.933--0.470) | 8.303(0.000-41.391) | -0.247(-81.877-0.242) |  |
| Slovenia | 0.565(-0.000-2.808) | -0.647(-0.860--0.575) | 8.337(-0.000-41.523) | -0.064(-61.655-0.520) |  | 11.928(-0.000-59.182) | -0.686(-0.834--0.618) | 8.314(-0.000-41.430) | -0.042(-41.442-0.342) |  |
| Solomon Islands | 1.518(0.000-7.895) | -0.209(-1.047-0.081) | 7.847(0.000-40.076) | 1.744(-105.881-12.543) |  | 38.267(0.000-203.857) | -0.192(-1.255-0.184) | 7.452(0.000-38.753) | 3.002(-136.105-23.311) |  |
| Somalia | 0.939(0.000-4.853) | -0.217(-0.960--0.038) | 7.890(0.000-40.288) | -4.097(-95.022--1.441) |  | 23.870(0.000-123.469) | -0.243(-0.876--0.053) | 7.680(0.000-39.486) | -5.853(-84.632--2.173) |  |
| South Africa | 0.370(0.000-2.095) | -0.244(-0.962--0.122) | 5.973(0.000-33.649) | -9.891(-95.263--2.299) |  | 9.274(0.000-51.662) | -0.310(-1.034--0.203) | 6.128(0.000-34.142) | -11.329(-104.298--4.669) |  |
| South Korea | 1.110(0.000-5.497) | -0.759(-0.954--0.675) | 8.331(0.000-41.486) | 0.176(-80.907-16.791) |  | 24.078(0.000-117.949) | -0.797(-0.986--0.710) | 8.297(0.000-41.304) | 0.341(-92.796-17.961) |  |
| South Sudan | 0.753(0.000-3.940) | -0.161(-0.973-0.118) | 7.892(0.000-40.317) | -4.162(-96.676--1.519) |  | 18.487(0.000-97.004) | -0.180(-1.004-0.106) | 7.676(0.000-39.528) | -6.037(-100.456--2.491) |  |
| Spain | 0.356(0.000-2.034) | -0.616(-0.982-3.874) | 5.870(0.000-33.232) | 4.085(-97.761-1191.919) |  | 8.780(0.000-47.772) | -0.609(-1.258-3.258) | 6.526(0.000-35.551) | 6.722(-172.795-1079.590) |  |
| Sri Lanka | 0.294(0.000-1.542) | -0.598(-0.905--0.449) | 8.275(0.000-41.377) | -0.170(-76.077-0.735) |  | 7.182(0.000-37.421) | -0.593(-0.930--0.439) | 8.219(0.000-41.143) | 0.156(-82.792-0.929) |  |
| Sudan | 0.778(0.000-5.004) | -0.289(-0.993-2.086) | 5.164(-0.000-30.624) | -2.284(-98.507-357.687) |  | 19.545(-0.000-123.649) | -0.330(-0.980-2.267) | 5.360(-0.000-31.328) | -1.759(-96.642-420.156) |  |
| Suriname | 0.521(-0.000-2.873) | -0.388(-1.090--0.053) | 7.495(-0.000-39.415) | -1.445(-112.850-43.048) |  | 12.638(-0.000-69.060) | -0.376(-1.074-0.161) | 7.385(-0.000-38.846) | -0.946(-111.162-75.687) |  |
| Swaziland | 0.616(0.000-3.488) | -0.199(-1.075-0.236) | 6.465(-0.000-36.241) | -5.764(-110.585-39.856) |  | 16.448(-0.000-93.580) | -0.163(-1.264-0.373) | 6.696(-0.000-37.091) | -5.287(-126.185-49.338) |  |
| Sweden | 0.253(0.000-1.324) | -0.639(-1.014--0.400) | 7.456(0.000-38.907) | -0.266(-104.253-64.512) |  | 5.160(0.000-26.963) | -0.661(-0.942--0.428) | 7.532(0.000-39.377) | -0.229(-83.758-69.634) |  |
| Switzerland | 0.282(0.000-1.493) | -0.643(-0.883-0.071) | 7.497(0.000-39.428) | 0.955(-66.045-196.096) |  | 6.111(0.000-31.852) | -0.655(-0.919-0.070) | 7.615(0.000-39.583) | 1.570(-74.640-206.723) |  |
| Syria | 0.305(0.000-1.815) | -0.273(-1.007-1.001) | 4.990(-0.000-30.135) | -3.624(-100.924-163.784) |  | 7.231(-0.000-42.501) | -0.339(-1.010-0.687) | 5.291(-0.000-31.648) | -3.078(-101.720-135.020) |  |
| Taiwan(China) | 0.721(0.000-3.655) | -0.546(-0.965--0.469) | 8.157(0.000-41.181) | -0.810(-92.482-14.058) |  | 15.520(0.000-79.596) | -0.593(-0.967--0.523) | 8.026(0.000-40.807) | -1.317(-91.901-6.462) |  |
| Tajikistan | 1.122(0.000-5.997) | -0.516(-0.995--0.407) | 7.775(0.000-40.267) | -4.389(-99.147--1.339) |  | 28.123(0.000-150.177) | -0.549(-0.995--0.439) | 7.713(0.000-40.007) | -4.561(-99.127--1.395) |  |
| Tanzania | 0.505(0.000-2.533) | -0.350(-1.007--0.154) | 8.187(0.000-41.067) | -1.010(-100.963-2.865) |  | 12.123(0.000-60.892) | -0.376(-0.925--0.163) | 8.052(0.000-40.601) | -1.882(-90.129-2.138) |  |
| Thailand | 0.597(0.000-3.183) | -0.370(-1.002--0.171) | 8.262(0.000-41.268) | -0.514(-100.351-0.148) |  | 16.019(-0.000-85.399) | -0.337(-0.964--0.100) | 8.193(-0.000-40.916) | -0.680(-92.964-0.054) |  |
| The Bahamas | 0.660(-0.000-3.609) | -0.363(-0.963-0.041) | 7.477(-0.000-39.202) | -1.115(-94.110-55.260) |  | 15.773(-0.000-86.670) | -0.393(-0.942-0.359) | 7.360(-0.000-38.738) | -0.916(-89.449-126.452) |  |
| The Gambia | 0.256(0.000-1.364) | -0.148(-1.026-2.011) | 7.330(0.000-38.820) | 1.737(-103.282-281.891) |  | 6.221(0.000-33.069) | -0.169(-1.010-1.704) | 7.289(0.000-38.697) | 1.381(-101.492-247.778) |  |
| Timor-Leste | 0.643(0.000-3.311) | -0.273(-0.876-0.046) | 8.273(0.000-41.301) | -0.369(-85.199-0.537) |  | 15.321(0.000-78.813) | -0.315(-0.874--0.035) | 8.210(0.000-41.001) | -0.410(-79.970-0.383) |  |
| Togo | 0.864(0.000-4.822) | -0.018(-0.771-3.818) | 7.355(0.000-38.763) | 2.334(-74.087-426.057) |  | 20.307(0.000-115.372) | -0.047(-0.723-3.740) | 7.329(0.000-38.731) | 2.207(-69.301-435.945) |  |
| Tokelau | 0.855(-0.000-4.388) | -0.386(-0.967--0.174) | 7.892(-0.000-40.106) | 1.103(-95.047-12.290) |  | 19.867(-0.000-102.665) | -0.380(-1.008--0.125) | 7.439(-0.000-38.518) | 1.554(-101.443-21.548) |  |
| Tonga | 1.331(0.000-6.725) | -0.191(-0.993-0.098) | 7.969(0.000-40.379) | 1.755(-99.082-16.372) |  | 30.564(0.000-159.747) | -0.193(-0.984-0.211) | 7.616(0.000-39.085) | 3.142(-98.099-29.152) |  |
| Trinidad and Tobago | 0.386(0.000-2.021) | -0.556(-1.020--0.033) | 7.496(0.000-39.470) | -0.086(-104.992-117.618) |  | 9.440(0.000-49.149) | -0.520(-1.052-0.051) | 7.446(0.000-39.293) | -0.430(-111.935-109.840) |  |
| Tunisia | 0.214(0.000-1.282) | -0.374(-0.990-2.401) | 5.028(-0.000-29.550) | -1.942(-98.537-429.953) |  | 5.290(-0.000-31.842) | -0.370(-1.020-1.838) | 5.313(-0.000-30.676) | -1.180(-101.812-367.104) |  |
| Turkey | 0.506(0.000-3.176) |  | 4.351(0.000-27.256) |  |  | 12.533(0.000-76.780) |  | 4.661(0.000-28.563) |  |  |
| Turkmenistan | 0.745(-0.000-3.898) | -0.581(-1.008--0.476) | 7.741(-0.000-40.073) | -4.538(-102.080--1.252) |  | 20.093(-0.000-105.895) | -0.577(-1.023--0.465) | 7.696(-0.000-39.788) | -4.499(-105.613--1.326) |  |
| Tuvalu | 1.183(0.000-6.121) | -0.333(-0.910--0.143) | 7.899(0.000-40.152) | 2.484(-86.807-21.737) |  | 28.449(0.000-151.147) | -0.343(-1.096--0.100) | 7.517(0.000-38.744) | 4.243(-111.574-26.570) |  |
| Uganda | 0.501(0.000-2.583) | -0.331(-0.918--0.131) | 7.895(0.000-40.122) | -4.139(-87.987--1.356) |  | 11.949(0.000-61.488) | -0.351(-0.959--0.151) | 7.660(0.000-39.228) | -6.223(-93.210--2.315) |  |
| Ukraine | 0.713(-0.000-3.961) | -0.610(-1.138--0.277) | 6.833(-0.000-37.257) | -1.266(-133.634-77.334) |  | 19.948(-0.000-110.437) | -0.613(-1.018--0.263) | 6.943(-0.000-37.565) | -0.981(-105.139-85.183) |  |
| United Arab Emirates | 0.392(0.000-2.409) | -0.493(-0.997-0.734) | 4.921(-0.000-29.760) | -6.618(-99.438-195.030) |  | 8.116(-0.000-48.988) | -0.573(-0.997-0.714) | 5.185(-0.000-30.768) | -5.619(-99.277-308.563) |  |
| United Kingdom | 0.315(-0.000-1.705) | -0.612(-1.116--0.103) | 6.819(-0.000-37.299) | 2.310(-130.497-137.661) |  | 6.218(-0.000-33.883) | -0.626(-0.939--0.009) | 6.664(-0.000-36.547) | 3.789(-82.966-177.930) |  |
| United States | 0.217(0.000-1.126) | -0.424(-0.466-6.870) | 7.655(0.000-39.747) | 8.627(2.088-1402.213) |  | 5.381(0.000-27.397) | -0.397(-0.703-8.709) | 7.775(0.000-40.033) | 8.929(-45.522-1660.285) |  |
| Uruguay | 0.872(0.000-4.420) | -0.354(-0.942--0.027) | 8.123(0.000-41.128) | -0.486(-90.507-45.771) |  | 20.111(0.000-102.223) | -0.347(-0.892--0.046) | 8.096(0.000-40.960) | -0.568(-83.148-45.283) |  |
| Uzbekistan | 0.595(0.000-3.033) | -0.645(-1.007--0.582) | 7.740(0.000-39.517) | -4.567(-101.690--0.920) |  | 15.990(0.000-81.552) | -0.656(-1.023--0.588) | 7.696(0.000-39.403) | -4.609(-106.476--0.978) |  |
| Vanuatu | 1.287(0.000-6.629) | -0.207(-0.924-0.032) | 7.879(0.000-40.101) | 1.728(-90.472-12.113) |  | 31.586(0.000-167.940) | -0.193(-0.914-0.137) | 7.488(0.000-38.515) | 3.081(-88.846-24.992) |  |
| Venezuela | 0.878(0.000-4.499) | -0.466(-0.787--0.091) | 8.136(0.000-40.995) | -0.302(-60.818-48.115) |  | 21.229(0.000-108.692) | -0.446(-0.784--0.066) | 8.070(0.000-40.661) | -0.759(-62.320-52.539) |  |
| Vietnam | 0.641(0.000-3.154) | -0.477(-1.126--0.294) | 8.292(0.000-41.417) | -0.384(-128.224-0.390) |  | 16.316(0.000-81.424) | -0.490(-1.087--0.303) | 8.259(0.000-41.275) | -0.432(-120.219-0.260) |  |
| Virgin Islands, U.S. | 0.418(-0.000-2.151) | -0.540(-1.083--0.271) | 7.602(-0.000-39.441) | -0.727(-113.844-59.952) |  | 9.819(-0.000-51.139) | -0.530(-0.993--0.157) | 7.451(-0.000-38.640) | -0.829(-95.953-88.104) |  |
| Yemen | 0.980(0.000-6.441) | -0.246(-1.055-2.119) | 5.102(-0.000-30.342) | -4.147(-105.970-235.160) |  | 24.511(-0.000-160.971) | -0.299(-1.103-1.607) | 5.324(-0.000-31.185) | -4.119(-110.742-224.212) |  |
| Zambia | 0.589(0.000-3.102) | -0.313(-0.961--0.115) | 7.877(0.000-40.230) | -4.135(-93.865--1.389) |  | 14.424(0.000-77.576) | -0.338(-0.990--0.137) | 7.639(0.000-39.309) | -6.035(-98.713--2.372) |  |
| Zimbabwe | 1.023(0.000-5.367) | 0.166(-0.719-1.646) | 7.375(0.000-38.623) | 0.159(-73.609-127.270) |  | 26.082(0.000-140.730) | 0.250(-0.669-1.381) | 7.444(0.000-38.760) | -0.096(-70.424-98.993) |  |

Note: DALY=disability adjusted life-years; PAF=population attributable fraction.

**Table S2. Age-standardized deaths and DALYs attributable to smoking in 2021 and percentage change from 1990 to 2021, by gender, SDI quintile and 204 countries.**

|  | Deaths | | | |  | DALYs | | | | |  |
| --- | --- | --- | --- | --- | --- | --- | --- | --- | --- | --- | --- |
|  | 2021 age-standardized  rate per  100,000 people | Percentage  change in age-standardized rate, 1990–2021 | 2021 age-standardized PAF | Percentage  change in age standardized  PAF, 1990–2021 |  | 2021 age-standardized  rate per  100,000 people | Percentage  change in age-standardized rate, 1990–2021 | 2021 age-standardized PAF | Percentage  change in age standardized  PAF, 1990–2021 |  |  |
|  |  |  |  |  |  |  |  |  |  |  | |
|  |  |  |  |  |  |  |  |  |  |  | |
| Global | 1.254(0.981-1.605) | -0.553(-0.629--0.459) | 11.181(9.283-13.158) | -0.592(-0.664--0.503) |  | 29.006(22.746-37.324) | -12.303(-20.147--3.864) | 11.020(9.138-12.851) | -13.289(-21.018--4.752) |  | |
| Sex |  |  |  |  |  |  |  |  |  |  | |
| Male | 2.562(1.990-3.298) | -0.547(-0.626--0.446) | 15.959(13.456-18.706) | -0.586(-0.662--0.488) |  | 57.567(44.842-74.594) | -14.184(-19.085--8.552) | 15.488(13.071-18.018) | -14.434(-18.710--9.376) |  | |
| Female | 0.163(0.129-0.203) | -0.674(-0.717--0.628) | 2.292(1.827-2.768) | -0.687(-0.725--0.644) |  | 3.588(2.877-4.421) | -31.233(-37.654--24.333) | 2.168(1.753-2.591) | -29.270(-35.139--23.309) |  | |
| Socio-demographic index |  |  |  |  |  |  |  |  |  |  | |
| low | 0.357(0.233-0.445) | -0.389(-0.480--0.256) | 4.216(3.319-5.078) | -0.423(-0.506--0.303) |  | 8.645(5.720-10.792) | -14.130(-22.995--5.662) | 4.114(3.263-4.897) | -14.215(-22.786--5.416) |  | |
| Low-middle | 0.558(0.428-0.686) | -0.437(-0.514--0.336) | 7.225(5.886-8.589) | -0.461(-0.532--0.362) |  | 13.640(10.453-16.825) | -23.726(-28.810--17.546) | 7.076(5.814-8.346) | -23.227(-28.227--17.336) |  | |
| Middle | 1.633(1.231-2.178) | -0.534(-0.642--0.388) | 11.877(9.738-14.233) | -0.585(-0.685--0.452) |  | 37.476(28.132-50.226) | -4.593(-16.876-9.340) | 11.669(9.543-13.879) | -6.579(-18.103-6.821) |  | |
| High-Middle | 1.950(1.502-2.548) | -0.510(-0.604--0.392) | 13.032(10.866-15.222) | -0.558(-0.644--0.438) |  | 46.469(35.851-61.162) | 1.880(-7.594-12.699) | 13.129(10.956-15.248) | 0.255(-8.182-10.021) |  | |
| High | 0.734(0.598-0.891) | -0.696(-0.722--0.669) | 10.743(8.755-12.796) | -0.727(-0.747--0.703) |  | 15.836(12.986-18.913) | -29.505(-33.828--24.706) | 10.838(8.980-12.699) | -28.657(-32.676--24.456) |  | |
| Countries |  |  |  |  |  |  |  |  |  |  | |
| Afghanistan | 1.236(0.485-1.829) | -0.016(-0.324-0.417) | 3.559(2.072-4.689) | 18.731(-9.673-54.185) |  | 29.466(12.340-43.151) | -0.015(-0.336-0.416) | 3.231(1.919-4.212) | 23.559(-3.594-56.437) |  | |
| Albania | 2.074(1.471-2.931) | -0.376(-0.558--0.130) | 20.031(15.610-24.373) | 6.230(-14.855-30.318) |  | 42.553(29.860-58.757) | -0.402(-0.584--0.133) | 18.837(14.826-22.505) | 7.341(-13.653-32.284) |  | |
| Algeria | 0.440(0.295-0.606) | -0.503(-0.651--0.305) | 11.599(9.093-14.488) | -15.465(-30.171-5.180) |  | 8.228(5.487-11.427) | -0.520(-0.650--0.329) | 10.074(8.009-12.479) | -14.808(-27.658-2.233) |  | |
| American Samoa | 1.179(0.869-1.585) | -0.297(-0.463--0.089) | 6.684(5.312-8.188) | -15.936(-30.631--1.602) |  | 32.892(24.022-44.008) | -0.281(-0.454--0.056) | 7.575(6.053-9.122) | -13.885(-27.539--0.451) |  | |
| Andorra | 0.631(0.376-0.972) | -0.635(-0.787--0.446) | 9.260(6.595-12.688) | -28.735(-45.212--6.591) |  | 13.886(8.330-21.259) | -0.642(-0.795--0.437) | 9.826(7.297-13.094) | -26.748(-42.781--6.000) |  | |
| Angola | 0.387(0.256-0.526) | -0.467(-0.628--0.219) | 4.406(3.449-5.593) | -15.136(-28.240-0.166) |  | 10.212(6.675-13.961) | -0.476(-0.641--0.217) | 4.750(3.728-5.981) | -12.156(-25.309-5.457) |  | |
| Antigua and Barbuda | 0.521(0.393-0.674) | -0.489(-0.580--0.383) | 5.198(4.019-6.643) | -11.970(-24.095-3.181) |  | 11.481(8.855-14.637) | -0.516(-0.601--0.419) | 5.305(4.162-6.583) | -10.493(-21.824-2.822) |  | |
| Argentina | 0.694(0.554-0.854) | -0.500(-0.573--0.409) | 8.272(6.752-9.915) | -15.278(-23.913--5.131) |  | 18.227(14.703-22.235) | -0.505(-0.578--0.416) | 9.228(7.657-10.980) | -16.959(-25.015--8.326) |  | |
| Armenia | 1.776(1.428-2.156) | -0.529(-0.608--0.428) | 16.467(13.689-19.164) | -4.712(-14.116-6.898) |  | 41.747(33.890-50.550) | -0.584(-0.651--0.504) | 16.099(13.588-18.580) | -5.644(-13.874-5.172) |  | |
| Australia | 0.215(0.167-0.277) | -0.711(-0.755--0.659) | 5.690(4.537-7.076) | -43.777(-50.359--36.103) |  | 5.123(4.085-6.266) | -0.709(-0.750--0.661) | 6.298(5.136-7.641) | -41.974(-48.204--35.385) |  | |
| Austria | 0.486(0.382-0.605) | -0.718(-0.764--0.670) | 10.543(8.571-12.634) | 0.725(-10.644-12.904) |  | 11.567(9.202-14.327) | -0.711(-0.753--0.663) | 11.478(9.417-13.548) | 1.025(-8.776-11.001) |  | |
| Azerbaijan | 2.022(1.438-3.019) | -0.346(-0.524--0.050) | 14.074(11.163-17.379) | 17.884(-1.431-47.051) |  | 47.873(33.712-71.828) | -0.432(-0.587--0.168) | 13.635(11.006-16.606) | 13.803(-2.626-38.982) |  | |
| Bahrain | 0.708(0.476-1.021) | -0.596(-0.717--0.459) | 10.421(7.581-14.200) | -15.380(-35.636-7.274) |  | 13.457(9.272-19.399) | -0.630(-0.742--0.505) | 10.126(7.392-13.860) | -14.982(-34.389-7.609) |  | |
| Bangladesh | 0.552(0.339-0.761) | -0.551(-0.673--0.376) | 10.130(8.211-12.054) | -16.504(-26.019--6.084) |  | 12.979(8.022-18.220) | -0.591(-0.709--0.432) | 9.734(7.771-11.529) | -15.360(-24.981--3.831) |  | |
| Barbados | 0.366(0.248-0.506) | -0.593(-0.716--0.439) | 3.741(2.926-4.839) | -28.074(-41.466--14.167) |  | 7.651(5.178-10.478) | -0.607(-0.725--0.463) | 3.576(2.846-4.456) | -28.732(-39.047--16.625) |  | |
| Belarus | 1.600(1.161-2.102) | -0.610(-0.718--0.483) | 12.911(10.566-15.385) | -1.434(-16.053-15.958) |  | 42.267(30.734-55.201) | -0.625(-0.727--0.505) | 13.013(10.705-15.345) | -2.148(-14.891-12.855) |  | |
| Belgium | 0.502(0.385-0.629) | -0.726(-0.769--0.676) | 11.247(9.043-13.635) | -26.676(-35.250--17.272) |  | 11.068(8.678-13.619) | -0.713(-0.753--0.665) | 11.570(9.450-13.838) | -26.298(-33.380--18.571) |  | |
| Belize | 0.478(0.368-0.616) | -0.355(-0.485--0.201) | 5.090(4.150-6.210) | -16.156(-26.944--2.869) |  | 11.682(9.095-14.921) | -0.355(-0.488--0.198) | 5.186(4.272-6.285) | -17.513(-27.278--5.955) |  | |
| Benin | 0.167(0.102-0.226) | -0.547(-0.686--0.370) | 1.719(1.340-2.120) | -41.130(-52.501--28.529) |  | 4.170(2.548-5.662) | -0.555(-0.701--0.370) | 1.868(1.494-2.300) | -39.272(-50.669--26.046) |  | |
| Bermuda | 0.298(0.217-0.399) | -0.636(-0.718--0.532) | 6.106(4.700-7.736) | -2.151(-15.901-13.512) |  | 6.797(5.030-8.921) | -0.649(-0.731--0.548) | 6.502(5.153-7.883) | -1.340(-14.337-13.766) |  | |
| Bhutan | 0.260(0.156-0.412) | -0.382(-0.597--0.025) | 4.606(3.274-6.465) | -8.396(-28.818-20.225) |  | 5.598(3.359-8.914) | -0.459(-0.648--0.146) | 4.163(3.028-5.832) | -8.986(-28.601-14.496) |  | |
| Bolivia | 1.235(0.849-1.816) | -0.457(-0.612--0.239) | 3.802(2.990-4.777) | -14.467(-27.510--0.928) |  | 28.954(19.610-42.842) | -0.487(-0.644--0.256) | 4.044(3.212-4.981) | -13.393(-25.285-0.334) |  | |
| Bosnia and Herzegovina | 1.352(0.945-1.873) | -0.327(-0.490--0.106) | 15.552(12.594-18.553) | 7.071(-8.330-28.698) |  | 30.994(21.771-42.802) | -0.360(-0.517--0.151) | 15.493(12.743-18.228) | 8.977(-5.220-28.933) |  | |
| Botswana | 0.370(0.234-0.514) | -0.531(-0.675--0.307) | 6.435(5.119-7.835) | -12.101(-26.386-4.987) |  | 9.034(5.682-12.709) | -0.548(-0.692--0.327) | 6.748(5.432-8.166) | -10.710(-24.036-5.037) |  | |
| Brazil | 0.740(0.587-0.922) | -0.744(-0.778--0.708) | 7.541(6.050-9.268) | -48.323(-54.575--41.946) |  | 17.274(13.917-21.202) | -0.747(-0.775--0.717) | 7.233(5.884-8.768) | -50.219(-55.562--44.520) |  | |
| Brunei | 0.949(0.669-1.292) | -0.736(-0.802--0.647) | 9.355(7.138-11.734) | -38.613(-49.995--25.796) |  | 20.997(15.029-28.487) | -0.744(-0.809--0.655) | 8.888(6.871-11.093) | -38.197(-49.662--25.032) |  | |
| Bulgaria | 1.353(1.024-1.758) | -0.547(-0.653--0.410) | 11.963(9.752-14.115) | -9.422(-21.495-6.319) |  | 36.894(28.188-47.955) | -0.543(-0.650--0.409) | 13.433(10.999-15.748) | -9.997(-21.227-3.119) |  | |
| Burkina Faso | 0.217(0.129-0.297) | -0.262(-0.450--0.015) | 1.813(1.400-2.342) | -10.808(-25.510-8.361) |  | 5.781(3.441-7.835) | -0.285(-0.481--0.015) | 2.093(1.636-2.661) | -9.940(-25.086-7.368) |  | |
| Burundi | 0.234(0.146-0.332) | -0.606(-0.720--0.441) | 2.964(2.325-3.769) | -36.631(-48.266--21.801) |  | 6.144(3.777-8.648) | -0.615(-0.731--0.441) | 3.131(2.485-3.963) | -34.202(-46.145--17.830) |  | |
| Cambodia | 1.351(0.940-1.892) | -0.440(-0.603--0.180) | 12.913(10.467-15.903) | -10.407(-25.458-7.490) |  | 30.432(20.827-43.632) | -0.472(-0.639--0.221) | 11.820(9.523-14.508) | -8.790(-22.833-8.450) |  | |
| Cameroon | 0.226(0.130-0.321) | -0.366(-0.574--0.067) | 2.168(1.681-2.642) | -21.799(-35.798--3.893) |  | 6.056(3.531-8.783) | -0.383(-0.585--0.059) | 2.490(1.963-3.040) | -21.624(-36.434--3.611) |  | |
| Canada | 0.412(0.318-0.523) | -0.702(-0.755--0.652) | 10.662(8.495-13.352) | -40.568(-47.840--32.217) |  | 8.635(6.851-10.831) | -0.717(-0.760--0.670) | 10.225(8.254-12.468) | -42.434(-48.077--35.828) |  | |
| Cape Verde | 0.554(0.384-0.771) | -0.398(-0.566--0.170) | 2.292(1.819-2.853) | -18.903(-30.497--3.346) |  | 13.973(9.600-20.022) | -0.426(-0.595--0.184) | 2.726(2.194-3.357) | -17.253(-29.145--2.327) |  | |
| Central African Republic | 0.376(0.235-0.525) | -0.439(-0.597--0.200) | 2.846(2.115-3.674) | -24.635(-38.212--6.706) |  | 10.651(6.613-15.160) | -0.432(-0.603--0.165) | 3.089(2.344-3.936) | -22.648(-36.900--4.728) |  | |
| Chad | 0.380(0.250-0.540) | -0.096(-0.365-0.293) | 2.797(2.195-3.569) | -20.183(-32.881-0.997) |  | 9.305(5.944-13.388) | -0.098(-0.372-0.308) | 2.879(2.240-3.614) | -18.668(-32.566-1.962) |  | |
| Chile | 0.836(0.655-1.040) | -0.655(-0.709--0.595) | 5.517(4.452-6.717) | -22.083(-30.321--14.050) |  | 22.905(18.135-27.966) | -0.657(-0.711--0.596) | 6.828(5.626-8.107) | -20.623(-28.472--12.609) |  | |
| China | 3.097(2.271-4.300) | -0.503(-0.637--0.318) | 14.365(11.849-17.079) | 6.284(-7.816-22.542) |  | 71.071(52.056-99.293) | -0.556(-0.685--0.386) | 14.141(11.618-16.785) | 4.478(-8.197-18.969) |  | |
| Colombia | 0.469(0.349-0.624) | -0.750(-0.803--0.688) | 3.470(2.827-4.210) | -41.621(-48.782--33.498) |  | 12.217(9.102-16.203) | -0.747(-0.802--0.682) | 3.694(3.031-4.420) | -43.383(-49.727--36.278) |  | |
| Comoros | 0.287(0.182-0.418) | -0.467(-0.632--0.242) | 4.090(3.100-5.270) | -24.464(-36.497--8.181) |  | 6.628(4.252-9.651) | -0.495(-0.661--0.263) | 3.875(2.955-4.973) | -24.300(-36.447--8.102) |  | |
| Congo | 0.307(0.195-0.412) | -0.373(-0.549--0.147) | 3.458(2.639-4.397) | 7.020(-14.461-36.029) |  | 7.750(4.876-10.499) | -0.405(-0.582--0.179) | 3.556(2.748-4.474) | 6.933(-12.998-33.849) |  | |
| Cook Islands | 0.390(0.278-0.533) | -0.550(-0.661--0.397) | 6.294(4.955-7.887) | -14.699(-28.703-1.320) |  | 10.301(7.194-13.804) | -0.544(-0.667--0.376) | 7.021(5.674-8.737) | -13.261(-27.036-1.066) |  | |
| Costa Rica | 0.991(0.763-1.254) | -0.712(-0.763--0.654) | 5.957(4.753-7.207) | -37.389(-45.606--29.236) |  | 22.929(17.646-28.561) | -0.702(-0.752--0.644) | 5.796(4.713-6.934) | -37.848(-44.707--30.920) |  | |
| Cote d'Ivoire | 0.090(0.059-0.123) | -0.353(-0.526--0.073) | 2.605(2.046-3.277) | -15.551(-30.635-3.783) |  | 2.323(1.473-3.172) | -0.360(-0.544--0.079) | 2.792(2.192-3.428) | -14.872(-28.223-3.415) |  | |
| Croatia | 1.229(0.934-1.557) | -0.705(-0.764--0.627) | 14.517(11.921-17.313) | -16.419(-26.613--3.765) |  | 26.902(20.453-34.073) | -0.713(-0.772--0.637) | 14.403(11.976-17.048) | -16.624(-26.107--5.690) |  | |
| Cuba | 0.516(0.396-0.660) | -0.460(-0.585--0.303) | 9.402(7.637-11.224) | -22.374(-34.570--8.843) |  | 12.535(9.661-15.908) | -0.443(-0.568--0.291) | 9.878(8.146-11.675) | -20.787(-31.780--9.387) |  | |
| Cyprus | 0.691(0.470-0.943) | -0.552(-0.690--0.374) | 11.719(9.198-14.608) | -9.000(-26.303-12.587) |  | 14.366(9.901-19.378) | -0.534(-0.674--0.354) | 12.814(10.336-15.492) | -5.451(-21.151-13.771) |  | |
| Czech Republic | 0.580(0.441-0.734) | -0.737(-0.793--0.657) | 10.616(8.673-12.739) | -18.046(-29.952--3.870) |  | 13.765(10.487-17.521) | -0.742(-0.798--0.665) | 11.242(9.190-13.277) | -18.274(-29.259--6.898) |  | |
| Democratic Republic of the Congo | 0.203(0.128-0.288) | -0.364(-0.545--0.096) | 2.305(1.714-2.921) | -20.375(-34.924--4.506) |  | 5.539(3.484-7.752) | -0.346(-0.544--0.060) | 2.560(1.942-3.213) | -17.190(-32.441--0.522) |  | |
| Denmark | 0.644(0.494-0.802) | -0.665(-0.718--0.609) | 13.978(11.105-17.001) | -33.147(-40.410--26.047) |  | 13.468(10.445-16.606) | -0.676(-0.724--0.625) | 13.432(10.911-16.144) | -31.719(-37.930--25.199) |  | |
| Djibouti | 0.511(0.319-0.728) | -0.200(-0.451-0.196) | 6.764(5.255-8.392) | -3.310(-21.087-19.913) |  | 12.137(7.620-17.182) | -0.237(-0.490-0.174) | 6.652(5.218-8.280) | -4.064(-20.343-17.946) |  | |
| Dominica | 0.778(0.563-1.074) | -0.394(-0.521--0.205) | 3.828(3.011-4.744) | -13.063(-24.289-0.877) |  | 19.063(13.784-26.111) | -0.379(-0.510--0.186) | 4.198(3.363-5.149) | -11.756(-21.978-1.383) |  | |
| Dominican Republic | 0.610(0.418-0.841) | -0.342(-0.545--0.051) | 8.709(6.906-10.822) | -20.654(-36.290--4.074) |  | 13.362(9.300-18.349) | -0.305(-0.508--0.015) | 7.762(6.286-9.487) | -19.321(-33.219--4.380) |  | |
| Ecuador | 0.733(0.531-0.986) | -0.613(-0.711--0.494) | 3.930(3.166-4.750) | -34.906(-43.247--25.287) |  | 17.208(12.316-23.161) | -0.619(-0.721--0.495) | 4.069(3.323-4.921) | -35.244(-42.957--26.382) |  | |
| Egypt | 0.798(0.485-1.088) | 0.681(-0.248-1.434) | 11.060(8.731-13.628) | 5.567(-21.661-27.947) |  | 17.711(10.393-24.459) | 0.509(-0.276-1.156) | 10.504(8.400-12.814) | 4.602(-20.610-24.843) |  | |
| El Salvador | 0.600(0.423-0.820) | -0.035(-0.278-0.252) | 3.407(2.704-4.240) | 7.983(-8.119-25.909) |  | 16.326(11.676-22.073) | -0.013(-0.257-0.302) | 3.772(3.029-4.591) | 12.103(-2.381-28.825) |  | |
| Equatorial Guinea | 0.211(0.128-0.296) | -0.619(-0.747--0.373) | 3.210(2.385-4.139) | -10.873(-29.113-13.795) |  | 5.509(3.301-7.837) | -0.642(-0.769--0.406) | 3.542(2.607-4.617) | -7.711(-27.709-19.465) |  | |
| Eritrea | 0.174(0.107-0.248) | -0.494(-0.640--0.288) | 1.709(1.201-2.299) | -30.301(-45.116--11.746) |  | 5.174(3.127-7.684) | -0.514(-0.665--0.295) | 1.997(1.406-2.708) | -27.957(-43.742--7.712) |  | |
| Estonia | 0.913(0.698-1.168) | -0.654(-0.735--0.560) | 9.261(7.522-11.230) | -9.902(-22.342-4.553) |  | 23.614(18.109-29.719) | -0.685(-0.757--0.605) | 10.261(8.502-12.200) | -8.905(-19.107-3.148) |  | |
| Ethiopia | 0.096(0.063-0.136) | -0.672(-0.777--0.527) | 1.437(1.028-1.863) | -21.946(-41.556-0.475) |  | 2.424(1.595-3.414) | -0.704(-0.801--0.573) | 1.470(1.060-1.918) | -21.535(-41.688-1.452) |  | |
| Federated States of Micronesia | 1.430(0.984-2.016) | -0.313(-0.516--0.002) | 8.318(6.621-9.905) | -6.220(-19.297-9.274) |  | 41.935(28.085-58.743) | -0.307(-0.524-0.039) | 9.426(7.584-11.148) | -3.809(-16.269-10.570) |  | |
| Fiji | 0.429(0.177-0.627) | -0.456(-0.628--0.212) | 5.101(3.673-6.273) | -31.304(-41.572--17.865) |  | 11.607(4.765-17.229) | -0.467(-0.646--0.222) | 5.856(4.387-7.051) | -28.264(-38.188--15.857) |  | |
| Finland | 0.270(0.200-0.354) | -0.762(-0.804--0.713) | 6.486(5.027-8.077) | -25.328(-36.430--14.550) |  | 6.519(4.949-8.300) | -0.772(-0.809--0.727) | 7.315(5.829-8.887) | -24.355(-33.026--15.779) |  | |
| France | 0.403(0.311-0.512) | -0.692(-0.741--0.632) | 8.987(7.251-10.951) | -25.831(-34.746--15.061) |  | 9.835(7.749-12.294) | -0.658(-0.710--0.594) | 9.736(7.968-11.645) | -22.728(-30.829--13.887) |  | |
| Gabon | 0.246(0.154-0.329) | -0.331(-0.530--0.062) | 3.098(2.357-3.844) | 5.772(-12.263-29.213) |  | 6.537(4.068-8.882) | -0.340(-0.546--0.050) | 3.446(2.642-4.259) | 8.820(-8.831-32.141) |  | |
| Georgia | 1.576(1.267-1.925) | -0.237(-0.358--0.088) | 13.209(11.069-15.484) | 11.093(-3.161-25.162) |  | 39.945(32.357-48.327) | -0.314(-0.425--0.186) | 13.576(11.407-15.801) | 10.819(-2.556-23.550) |  | |
| Germany | 0.613(0.476-0.772) | -0.696(-0.748--0.623) | 10.575(8.396-12.886) | -23.301(-33.763--11.055) |  | 14.382(11.198-17.744) | -0.681(-0.733--0.609) | 10.958(8.818-13.163) | -22.411(-32.108--11.161) |  | |
| Ghana | 0.142(0.098-0.195) | -0.347(-0.552--0.025) | 1.831(1.399-2.309) | -17.604(-33.339-3.090) |  | 3.190(2.176-4.423) | -0.350(-0.556--0.049) | 1.823(1.398-2.284) | -12.979(-29.784-6.228) |  | |
| Greece | 1.245(1.008-1.531) | -0.588(-0.647--0.514) | 16.924(13.759-20.143) | -7.745(-17.915-4.839) |  | 27.369(22.214-33.215) | -0.582(-0.636--0.514) | 17.025(14.072-20.059) | -9.263(-18.789-1.800) |  | |
| Greenland | 1.115(0.776-1.575) | -0.683(-0.765--0.573) | 12.746(9.825-16.645) | -27.157(-40.324--11.271) |  | 28.232(20.224-39.146) | -0.690(-0.773--0.583) | 13.002(10.299-16.542) | -26.254(-39.429--10.572) |  | |
| Grenada | 0.310(0.229-0.394) | -0.525(-0.617--0.397) | 3.607(2.796-4.413) | -18.882(-30.303--7.112) |  | 7.827(5.886-9.946) | -0.545(-0.637--0.413) | 4.010(3.175-4.863) | -15.952(-26.906--4.763) |  | |
| Guam | 0.314(0.230-0.399) | -0.350(-0.483--0.185) | 6.376(5.134-7.712) | 23.868(6.808-43.996) |  | 9.948(7.340-12.621) | -0.258(-0.417--0.072) | 6.867(5.624-8.268) | 7.421(-6.425-25.037) |  | |
| Guatemala | 0.752(0.564-0.977) | -0.350(-0.490--0.183) | 3.080(2.438-3.764) | -13.201(-25.899-1.721) |  | 18.287(13.693-23.845) | -0.309(-0.453--0.128) | 3.142(2.518-3.795) | -12.961(-24.219-1.263) |  | |
| Guinea | 0.290(0.189-0.410) | -0.181(-0.436-0.242) | 2.938(2.280-3.645) | -7.567(-24.169-13.376) |  | 7.344(4.793-10.343) | -0.182(-0.445-0.273) | 3.023(2.346-3.727) | -6.340(-23.240-14.601) |  | |
| Guinea-Bissau | 0.295(0.191-0.393) | -0.125(-0.355-0.274) | 1.831(1.419-2.305) | 7.043(-11.185-27.839) |  | 8.131(5.329-10.952) | -0.124(-0.375-0.297) | 2.055(1.598-2.574) | 13.005(-6.698-36.618) |  | |
| Guyana | 0.327(0.223-0.463) | -0.528(-0.663--0.372) | 4.089(3.231-4.958) | -17.302(-26.953--6.268) |  | 8.858(6.031-12.702) | -0.509(-0.657--0.334) | 4.393(3.546-5.319) | -16.950(-26.396--5.473) |  | |
| Haiti | 0.492(0.309-0.711) | -0.525(-0.691--0.321) | 2.624(2.025-3.360) | -26.613(-36.283--15.206) |  | 12.442(7.663-17.915) | -0.565(-0.723--0.359) | 2.741(2.145-3.416) | -30.490(-40.065--20.018) |  | |
| Honduras | 1.011(0.697-1.392) | -0.038(-0.304-0.326) | 4.879(3.749-6.208) | -17.289(-31.778-6.811) |  | 23.339(16.550-31.957) | -0.106(-0.353-0.221) | 5.057(3.928-6.388) | -14.242(-28.977-9.604) |  | |
| Hungary | 0.761(0.579-0.967) | -0.723(-0.782--0.651) | 10.720(8.729-12.847) | -20.842(-30.731--7.545) |  | 19.439(14.795-24.573) | -0.720(-0.779--0.648) | 11.480(9.474-13.708) | -21.555(-31.174--9.872) |  | |
| Iceland | 0.504(0.373-0.646) | -0.796(-0.836--0.749) | 11.552(9.005-14.452) | -36.231(-43.774--26.318) |  | 10.433(7.836-13.362) | -0.807(-0.845--0.763) | 11.566(9.297-14.216) | -35.806(-43.068--27.153) |  | |
| India | 0.358(0.266-0.473) | -0.475(-0.584--0.340) | 6.120(4.916-7.420) | -28.908(-37.327--19.392) |  | 8.685(6.517-11.604) | -0.517(-0.611--0.392) | 5.808(4.690-7.051) | -29.515(-37.717--20.359) |  | |
| Indonesia | 0.655(0.475-0.897) | -0.149(-0.380-0.239) | 8.736(6.832-10.532) | 8.738(-8.410-29.075) |  | 15.905(11.430-21.933) | -0.202(-0.426-0.153) | 8.869(6.950-10.710) | 12.679(-5.544-32.820) |  | |
| Iran | 0.974(0.552-1.248) | -0.447(-0.550--0.312) | 7.318(5.701-9.048) | -0.013(-15.091-17.568) |  | 21.766(12.855-27.371) | -0.484(-0.580--0.357) | 7.414(5.884-9.048) | -0.691(-14.196-15.927) |  | |
| Iraq | 0.697(0.461-0.943) | -0.317(-0.537--0.023) | 13.217(10.658-15.960) | -14.283(-27.552-3.384) |  | 14.987(9.822-20.354) | -0.392(-0.594--0.108) | 11.817(9.553-14.203) | -13.677(-27.152-4.411) |  | |
| Ireland | 0.446(0.344-0.565) | -0.818(-0.848--0.783) | 9.671(7.651-11.933) | -46.568(-53.437--38.095) |  | 8.872(7.125-10.820) | -0.820(-0.848--0.790) | 9.395(7.569-11.292) | -45.276(-51.255--37.972) |  | |
| Israel | 0.465(0.363-0.590) | -0.661(-0.718--0.596) | 9.034(7.181-11.155) | -27.401(-38.322--15.229) |  | 10.182(8.066-12.637) | -0.660(-0.715--0.597) | 9.396(7.624-11.396) | -26.789(-36.296--15.695) |  | |
| Italy | 0.686(0.525-0.848) | -0.740(-0.780--0.699) | 9.433(7.424-11.471) | -30.046(-39.686--20.043) |  | 14.938(11.862-18.231) | -0.748(-0.781--0.711) | 9.839(7.926-11.794) | -29.692(-38.348--21.042) |  | |
| Jamaica | 0.550(0.369-0.784) | -0.538(-0.669--0.359) | 6.014(4.736-7.574) | -21.931(-32.780--9.835) |  | 13.068(8.665-18.721) | -0.534(-0.666--0.341) | 6.172(4.886-7.572) | -24.102(-33.897--13.737) |  | |
| Japan | 1.306(1.042-1.621) | -0.754(-0.787--0.717) | 9.899(7.997-12.090) | -37.013(-44.621--27.815) |  | 27.015(21.993-32.522) | -0.779(-0.804--0.750) | 10.000(8.169-11.866) | -35.283(-42.741--26.680) |  | |
| Jordan | 0.572(0.392-0.762) | -0.519(-0.675--0.316) | 14.740(12.192-17.716) | -8.357(-22.772-6.599) |  | 12.378(8.564-16.625) | -0.550(-0.692--0.362) | 14.118(11.721-16.932) | -5.119(-19.149-10.051) |  | |
| Kazakhstan | 0.840(0.658-1.060) | -0.725(-0.784--0.646) | 7.787(6.367-9.316) | -14.282(-28.126-1.565) |  | 23.633(18.789-29.236) | -0.737(-0.789--0.671) | 8.443(6.977-9.958) | -14.197(-27.183--0.226) |  | |
| Kenya | 0.271(0.196-0.370) | -0.290(-0.477-0.043) | 3.288(2.533-4.169) | -31.944(-43.579--17.027) |  | 6.742(4.896-9.097) | -0.282(-0.467-0.053) | 3.371(2.573-4.251) | -28.138(-40.098--12.314) |  | |
| Kiribati | 2.528(1.781-3.338) | -0.142(-0.359-0.212) | 11.451(9.510-13.395) | -3.663(-13.177-7.376) |  | 69.905(48.829-92.782) | -0.141(-0.376-0.232) | 12.134(10.187-14.128) | -0.532(-9.109-9.526) |  | |
| Kuwait | 0.304(0.217-0.408) | -0.305(-0.506--0.003) | 11.720(9.079-14.635) | 11.567(-10.455-41.020) |  | 6.080(4.330-8.194) | -0.399(-0.571--0.133) | 11.060(8.742-13.670) | 7.232(-13.119-33.024) |  | |
| Kyrgyzstan | 2.146(1.633-2.759) | -0.453(-0.576--0.294) | 14.028(11.585-16.505) | 12.112(-2.023-27.469) |  | 56.095(42.595-72.160) | -0.500(-0.611--0.367) | 13.753(11.367-16.193) | 10.212(-2.479-23.104) |  | |
| Laos | 0.947(0.656-1.355) | -0.527(-0.670--0.312) | 11.105(8.640-13.493) | -2.637(-18.400-16.817) |  | 22.205(15.551-31.486) | -0.568(-0.699--0.372) | 10.504(8.316-12.659) | -2.445(-16.130-14.429) |  | |
| Latvia | 1.024(0.774-1.312) | -0.600(-0.696--0.487) | 9.534(7.736-11.371) | -9.696(-21.169-3.844) |  | 28.582(21.594-36.245) | -0.613(-0.704--0.503) | 10.678(8.718-12.533) | -7.287(-16.881-4.492) |  | |
| Lebanon | 0.875(0.612-1.183) | -0.389(-0.546--0.165) | 16.210(13.023-19.409) | 21.180(3.181-42.733) |  | 18.758(13.495-25.255) | -0.445(-0.588--0.227) | 15.786(12.865-18.855) | 21.514(4.423-40.649) |  | |
| Lesotho | 1.012(0.630-1.390) | 0.381(-0.061-1.176) | 8.084(6.244-10.151) | 4.204(-20.318-40.384) |  | 26.509(16.024-36.586) | 0.564(0.066-1.483) | 8.186(6.418-10.068) | 10.119(-15.077-43.465) |  | |
| Liberia | 0.215(0.128-0.311) | -0.282(-0.509-0.062) | 2.099(1.614-2.633) | -19.806(-33.915--3.524) |  | 5.673(3.404-8.217) | -0.274(-0.515-0.100) | 2.385(1.879-2.953) | -17.342(-32.382--0.661) |  | |
| Libya | 0.632(0.405-0.891) | -0.229(-0.509-0.314) | 9.526(7.472-11.627) | -7.234(-27.358-24.929) |  | 14.424(9.386-20.265) | -0.245(-0.519-0.261) | 8.998(7.039-11.015) | -7.749(-26.275-23.541) |  | |
| Lithuania | 0.937(0.709-1.187) | -0.610(-0.700--0.522) | 8.817(7.222-10.512) | -11.076(-21.676-1.428) |  | 25.088(19.027-31.674) | -0.616(-0.703--0.520) | 9.441(7.816-11.153) | -8.311(-17.684-2.395) |  | |
| Luxembourg | 0.450(0.314-0.626) | -0.736(-0.798--0.646) | 10.088(7.025-13.923) | -21.004(-38.380-4.715) |  | 9.416(6.727-13.051) | -0.763(-0.817--0.684) | 10.658(7.632-14.606) | -20.946(-37.288-2.922) |  | |
| Macedonia | 1.952(1.357-2.658) | -0.437(-0.595--0.217) | 13.851(10.928-17.104) | -12.039(-27.736-8.036) |  | 44.710(31.053-60.371) | -0.474(-0.622--0.280) | 14.794(11.953-18.146) | -8.200(-22.985-10.007) |  | |
| Madagascar | 0.144(0.091-0.210) | -0.608(-0.721--0.448) | 2.231(1.714-2.764) | -45.530(-54.135--33.918) |  | 3.561(2.217-5.216) | -0.612(-0.726--0.451) | 2.167(1.658-2.690) | -44.816(-53.465--33.536) |  | |
| Malawi | 0.181(0.124-0.251) | -0.219(-0.416-0.096) | 5.572(4.341-6.944) | -2.771(-20.050-20.012) |  | 4.364(2.929-6.014) | -0.182(-0.392-0.161) | 5.330(4.099-6.668) | 2.843(-16.015-28.213) |  | |
| Malaysia | 0.455(0.341-0.595) | -0.470(-0.592--0.326) | 7.164(5.680-8.905) | -25.680(-38.581--9.638) |  | 10.420(8.091-13.888) | -0.480(-0.591--0.347) | 6.993(5.611-8.591) | -23.509(-35.553--9.003) |  | |
| Maldives | 0.382(0.270-0.527) | -0.710(-0.787--0.573) | 13.628(10.838-16.610) | -1.230(-16.610-17.482) |  | 7.494(5.341-10.343) | -0.746(-0.817--0.634) | 12.215(9.858-14.727) | 1.950(-12.857-20.856) |  | |
| Mali | 0.565(0.403-0.778) | 0.021(-0.265-0.404) | 3.310(2.620-4.040) | 35.339(13.404-61.512) |  | 13.251(9.273-18.485) | -0.024(-0.313-0.352) | 3.153(2.512-3.852) | 34.260(11.849-60.252) |  | |
| Malta | 0.472(0.358-0.613) | -0.739(-0.794--0.665) | 10.059(8.001-12.475) | -24.941(-36.561--10.620) |  | 10.906(8.443-14.028) | -0.737(-0.792--0.666) | 10.939(8.863-13.349) | -23.950(-34.834--10.948) |  | |
| Marshall Islands | 1.024(0.655-1.443) | -0.263(-0.500-0.052) | 5.699(4.314-7.307) | -3.080(-20.440-18.549) |  | 28.630(18.692-40.496) | -0.264(-0.501-0.067) | 6.077(4.639-7.753) | -3.288(-20.713-17.510) |  | |
| Mauritania | 0.225(0.142-0.316) | -0.445(-0.610--0.207) | 2.470(1.915-3.016) | -22.711(-36.145--4.612) |  | 5.704(3.615-7.955) | -0.484(-0.647--0.262) | 2.825(2.215-3.412) | -22.260(-35.632--5.887) |  | |
| Mauritius | 0.783(0.642-0.950) | -0.383(-0.479--0.289) | 7.849(6.536-9.326) | -1.401(-11.950-9.072) |  | 19.866(16.197-23.923) | -0.392(-0.486--0.298) | 7.868(6.595-9.225) | -4.967(-13.970-3.366) |  | |
| Mexico | 0.379(0.293-0.475) | -0.705(-0.755--0.642) | 4.098(3.338-5.002) | -46.901(-54.489--38.498) |  | 8.970(6.976-11.222) | -0.676(-0.728--0.610) | 4.013(3.288-4.802) | -47.815(-54.148--40.600) |  | |
| Moldova | 1.212(0.896-1.597) | -0.542(-0.654--0.395) | 14.463(11.228-18.282) | 10.552(-8.151-35.341) |  | 32.864(24.393-42.913) | -0.558(-0.666--0.416) | 14.762(11.475-18.558) | 8.772(-9.614-33.109) |  | |
| Monaco | 0.760(0.502-1.123) | -0.532(-0.676--0.321) | 10.697(7.655-15.094) | -20.679(-38.218-2.011) |  | 16.968(11.082-25.416) | -0.543(-0.695--0.330) | 11.075(8.136-15.451) | -20.948(-38.135--0.865) |  | |
| Mongolia | 3.417(2.443-4.493) | -0.200(-0.440-0.121) | 9.137(7.267-11.141) | 20.778(1.555-45.416) |  | 92.885(66.289-126.458) | -0.206(-0.452-0.121) | 9.976(7.984-12.034) | 24.911(6.827-48.729) |  | |
| Montenegro | 1.161(0.820-1.568) | -0.243(-0.438-0.011) | 14.696(11.491-18.127) | -16.802(-31.423-2.654) |  | 27.150(19.447-36.658) | -0.300(-0.479--0.051) | 15.955(12.656-19.321) | -12.857(-28.574-5.938) |  | |
| Morocco | 0.116(0.073-0.159) | -0.467(-0.626--0.219) | 4.597(3.573-5.769) | -31.273(-44.349--12.567) |  | 2.876(1.844-3.967) | -0.478(-0.645--0.226) | 4.785(3.768-5.961) | -28.941(-43.349--12.853) |  | |
| Mozambique | 0.294(0.189-0.397) | -0.174(-0.402-0.157) | 3.633(2.830-4.517) | -13.141(-28.195-3.566) |  | 7.079(4.639-9.753) | -0.128(-0.377-0.206) | 3.832(3.039-4.696) | -9.348(-23.138-6.494) |  | |
| Myanmar | 0.578(0.417-0.790) | -0.722(-0.802--0.590) | 7.831(6.355-9.416) | -41.043(-48.311--32.990) |  | 13.444(9.655-18.509) | -0.744(-0.821--0.620) | 7.367(6.005-8.820) | -40.169(-47.192--31.556) |  | |
| Namibia | 0.154(0.108-0.204) | -0.388(-0.554--0.164) | 5.618(4.611-6.873) | -22.345(-33.937--9.484) |  | 3.449(2.452-4.612) | -0.393(-0.570--0.169) | 4.982(4.116-6.022) | -21.793(-32.269--9.877) |  | |
| Nauru | 1.514(1.031-2.034) | -0.348(-0.538--0.066) | 7.256(5.865-8.705) | -15.380(-26.902--2.657) |  | 45.032(30.782-61.490) | -0.322(-0.524--0.029) | 8.079(6.613-9.649) | -12.224(-23.193--0.625) |  | |
| Nepal | 0.496(0.332-0.687) | -0.526(-0.662--0.326) | 7.931(6.478-9.470) | -32.812(-39.870--25.549) |  | 11.027(7.432-15.441) | -0.575(-0.700--0.381) | 7.180(5.852-8.581) | -33.912(-40.620--26.750) |  | |
| Netherlands | 0.513(0.395-0.644) | -0.793(-0.828--0.756) | 11.096(8.815-13.622) | -42.361(-49.252--33.653) |  | 10.292(8.108-12.632) | -0.805(-0.836--0.774) | 10.863(8.805-13.151) | -43.123(-49.371--36.176) |  | |
| New Zealand | 0.362(0.286-0.454) | -0.660(-0.706--0.607) | 7.896(6.343-9.673) | -29.429(-36.453--22.145) |  | 8.440(6.838-10.387) | -0.666(-0.708--0.620) | 8.175(6.692-9.731) | -29.673(-34.743--24.183) |  | |
| Nicaragua | 0.466(0.338-0.637) | -0.382(-0.518--0.201) | 4.766(3.761-5.931) | -12.053(-26.890-4.646) |  | 11.554(8.332-15.648) | -0.378(-0.523--0.202) | 4.904(3.914-6.069) | -11.281(-24.538-3.431) |  | |
| Niger | 0.186(0.116-0.259) | -0.225(-0.436-0.072) | 1.606(1.229-2.075) | -10.038(-27.203-10.402) |  | 4.018(2.439-5.592) | -0.290(-0.485--0.025) | 1.519(1.155-1.930) | -12.222(-27.057-4.383) |  | |
| Nigeria | 0.028(0.019-0.041) | -0.571(-0.722--0.321) | 1.057(0.794-1.352) | -40.105(-53.096--24.948) |  | 0.700(0.473-1.035) | -0.570(-0.717--0.297) | 1.152(0.875-1.472) | -36.096(-50.318--19.012) |  | |
| Niue | 0.672(0.474-0.936) | -0.298(-0.485--0.047) | 5.783(4.402-7.665) | -9.992(-28.143-11.755) |  | 17.822(12.616-24.846) | -0.322(-0.518--0.052) | 6.369(4.955-8.132) | -10.906(-27.481-8.728) |  | |
| North Korea | 2.134(1.480-2.890) | -0.247(-0.460-0.050) | 9.893(7.894-11.979) | -3.004(-17.765-14.469) |  | 60.682(41.477-83.614) | -0.232(-0.454-0.086) | 10.356(8.310-12.363) | -3.019(-16.152-12.034) |  | |
| Northern Mariana Islands | 0.766(0.581-1.002) | -0.369(-0.534--0.190) | 5.613(4.506-6.968) | -18.641(-31.505--3.931) |  | 20.532(15.666-26.663) | -0.370(-0.527--0.180) | 6.531(5.304-8.014) | -16.374(-29.094--2.771) |  | |
| Norway | 0.228(0.178-0.282) | -0.829(-0.857--0.800) | 5.986(4.757-7.284) | -50.576(-57.698--42.560) |  | 5.099(4.084-6.164) | -0.834(-0.856--0.812) | 6.622(5.357-7.896) | -48.017(-54.092--42.033) |  | |
| Oman | 0.249(0.165-0.344) | -0.635(-0.760--0.453) | 4.656(3.565-5.804) | -23.547(-38.108--5.555) |  | 5.405(3.663-7.338) | -0.679(-0.788--0.521) | 4.663(3.633-5.625) | -23.057(-36.343--7.104) |  | |
| Pakistan | 0.325(0.225-0.453) | -0.468(-0.627--0.236) | 6.688(5.150-8.190) | -39.699(-50.505--26.396) |  | 7.751(5.347-10.904) | -0.456(-0.620--0.212) | 6.345(4.826-7.864) | -38.124(-49.865--23.770) |  | |
| Palau | 0.962(0.666-1.334) | -0.383(-0.564--0.124) | 5.073(3.975-6.402) | -16.957(-32.229-1.821) |  | 26.185(18.246-35.944) | -0.360(-0.551--0.085) | 5.534(4.394-6.873) | -14.242(-27.942-3.672) |  | |
| Palestine | 0.722(0.473-1.012) | -0.540(-0.674--0.313) | 11.315(8.505-14.715) | -13.116(-29.786-10.760) |  | 15.377(10.381-21.104) | -0.538(-0.677--0.295) | 11.044(8.474-14.354) | -9.496(-26.933-15.431) |  | |
| Panama | 0.409(0.298-0.549) | -0.557(-0.681--0.421) | 4.079(3.262-5.038) | -36.846(-45.728--26.898) |  | 9.206(6.667-12.411) | -0.550(-0.672--0.409) | 3.919(3.171-4.765) | -37.268(-45.325--28.633) |  | |
| Papua New Guinea | 0.685(0.448-0.988) | -0.368(-0.576--0.053) | 4.828(3.799-6.070) | -16.657(-30.126--2.127) |  | 19.774(12.812-29.110) | -0.367(-0.585--0.032) | 5.578(4.362-6.973) | -13.748(-27.311-1.947) |  | |
| Paraguay | 0.818(0.553-1.159) | -0.453(-0.612--0.244) | 9.363(7.454-11.489) | -34.729(-46.280--23.453) |  | 17.935(12.104-25.037) | -0.445(-0.603--0.226) | 8.902(7.142-10.820) | -32.976(-43.708--22.035) |  | |
| Peru | 0.638(0.432-0.877) | -0.332(-0.524--0.070) | 3.194(2.504-3.973) | -4.195(-17.932-11.382) |  | 14.996(10.072-20.721) | -0.344(-0.540--0.081) | 3.289(2.645-4.021) | -2.149(-15.500-12.264) |  | |
| Philippines | 0.399(0.296-0.567) | -0.451(-0.571--0.295) | 9.102(7.430-11.051) | -29.674(-37.835--18.401) |  | 10.205(7.561-14.410) | -0.437(-0.561--0.252) | 9.094(7.418-10.964) | -27.713(-35.551--18.788) |  | |
| Poland | 0.901(0.722-1.113) | -0.704(-0.745--0.660) | 10.690(8.795-12.772) | -27.620(-35.089--20.025) |  | 22.280(18.062-27.100) | -0.715(-0.752--0.673) | 11.314(9.457-13.246) | -28.466(-35.038--21.760) |  | |
| Portugal | 0.708(0.565-0.872) | -0.729(-0.777--0.669) | 6.586(5.274-8.003) | -23.498(-35.333--8.012) |  | 18.693(15.154-22.644) | -0.712(-0.761--0.653) | 7.692(6.262-9.147) | -19.454(-30.521--6.459) |  | |
| Puerto Rico | 0.242(0.169-0.359) | -0.713(-0.785--0.608) | 5.706(4.214-7.729) | -18.699(-34.243-0.586) |  | 5.755(4.055-8.339) | -0.689(-0.767--0.582) | 5.804(4.462-7.605) | -17.282(-32.111--0.088) |  | |
| Qatar | 0.371(0.229-0.549) | -0.749(-0.835--0.605) | 6.367(4.498-8.743) | -30.550(-45.938--7.485) |  | 7.755(4.797-11.482) | -0.759(-0.850--0.620) | 6.721(4.885-9.101) | -29.188(-44.489--5.560) |  | |
| Romania | 0.999(0.766-1.267) | -0.403(-0.520--0.260) | 9.622(7.957-11.379) | -13.730(-25.688--3.073) |  | 27.354(21.184-33.960) | -0.418(-0.528--0.289) | 10.639(8.923-12.418) | -11.240(-21.195--1.466) |  | |
| Russian Federation | 1.338(1.084-1.613) | -0.582(-0.637--0.527) | 10.288(8.636-12.123) | 1.465(-9.157-11.889) |  | 35.894(29.318-43.065) | -0.617(-0.666--0.570) | 11.258(9.458-13.137) | 1.776(-7.245-10.813) |  | |
| Rwanda | 0.532(0.363-0.730) | -0.433(-0.613--0.200) | 7.698(6.165-9.380) | 11.877(-5.416-31.212) |  | 11.878(8.134-16.242) | -0.484(-0.652--0.265) | 7.112(5.776-8.555) | 12.856(-4.145-32.253) |  | |
| Saint Kitts and Nevis | 0.321(0.227-0.428) | -0.547(-0.652--0.415) | 3.506(2.691-4.494) | -14.887(-28.244-1.776) |  | 7.206(5.065-9.658) | -0.572(-0.679--0.448) | 3.616(2.843-4.543) | -12.907(-25.063-1.936) |  | |
| Saint Lucia | 0.518(0.379-0.691) | -0.596(-0.684--0.480) | 4.743(3.749-5.917) | -18.352(-28.659--7.280) |  | 12.441(9.235-16.574) | -0.602(-0.689--0.489) | 5.029(4.055-6.112) | -19.204(-29.154--10.034) |  | |
| Saint Vincent and the Grenadines | 0.443(0.333-0.573) | -0.432(-0.543--0.285) | 4.239(3.423-5.243) | -2.207(-14.120-11.075) |  | 11.224(8.509-14.411) | -0.445(-0.559--0.303) | 4.609(3.763-5.584) | -4.171(-14.798-7.801) |  | |
| Samoa | 0.889(0.541-1.176) | -0.324(-0.486--0.105) | 8.411(6.656-10.343) | -15.329(-30.702-2.884) |  | 22.503(13.932-29.539) | -0.323(-0.496--0.101) | 8.537(6.826-10.394) | -16.430(-30.593--0.408) |  | |
| San Marino | 0.981(0.561-1.631) | -0.727(-0.837--0.579) | 10.277(7.382-14.277) | -22.611(-40.938-3.347) |  | 20.919(11.877-34.646) | -0.727(-0.843--0.572) | 10.531(7.694-14.453) | -23.901(-40.846--0.356) |  | |
| Sao Tome and Principe | 0.293(0.203-0.400) | -0.040(-0.264-0.298) | 1.896(1.441-2.412) | 6.817(-11.451-31.097) |  | 7.453(5.289-10.131) | -0.060(-0.286-0.312) | 2.243(1.721-2.793) | 7.584(-10.505-31.170) |  | |
| Saudi Arabia | 0.236(0.156-0.391) | -0.438(-0.614--0.108) | 6.533(4.756-8.558) | 1.349(-20.014-32.207) |  | 5.725(3.760-9.969) | -0.444(-0.630--0.109) | 6.674(4.897-8.759) | 0.786(-20.472-31.607) |  | |
| Senegal | 0.236(0.147-0.329) | -0.460(-0.630--0.208) | 2.181(1.650-2.701) | -36.992(-48.123--22.920) |  | 6.449(4.073-9.065) | -0.486(-0.643--0.230) | 2.606(2.015-3.204) | -36.410(-46.782--21.999) |  | |
| Serbia | 0.882(0.637-1.194) | -0.430(-0.585--0.238) | 11.438(9.323-13.981) | 2.811(-11.357-21.814) |  | 22.434(16.232-30.394) | -0.426(-0.588--0.234) | 12.478(10.207-15.046) | 3.563(-8.947-19.649) |  | |
| Seychelles | 0.592(0.441-0.798) | -0.503(-0.617--0.361) | 9.811(7.843-12.108) | -11.571(-24.224-5.747) |  | 14.227(10.511-18.964) | -0.531(-0.636--0.398) | 9.571(7.821-11.565) | -10.635(-22.551-5.847) |  | |
| Sierra Leone | 0.312(0.200-0.434) | -0.391(-0.567--0.147) | 2.977(2.326-3.673) | -31.856(-42.388--19.764) |  | 8.265(5.354-11.522) | -0.390(-0.578--0.135) | 3.364(2.682-4.120) | -30.833(-41.579--18.860) |  | |
| Singapore | 0.222(0.168-0.288) | -0.849(-0.881--0.809) | 4.889(3.818-6.257) | -44.827(-55.530--33.272) |  | 4.701(3.657-5.911) | -0.864(-0.891--0.833) | 5.088(4.043-6.261) | -42.821(-53.252--32.585) |  | |
| Slovakia | 0.722(0.502-0.970) | -0.631(-0.732--0.524) | 8.667(6.792-10.565) | -20.221(-32.953--4.956) |  | 17.980(12.719-23.879) | -0.647(-0.749--0.540) | 9.408(7.510-11.240) | -18.983(-30.002--5.594) |  | |
| Slovenia | 0.737(0.546-0.980) | -0.680(-0.745--0.593) | 10.852(8.498-13.483) | -10.197(-21.926-3.482) |  | 17.637(13.252-23.177) | -0.710(-0.769--0.629) | 12.245(9.756-14.895) | -8.171(-19.700-5.329) |  | |
| Solomon Islands | 1.690(1.172-2.361) | -0.281(-0.499-0.024) | 8.665(7.079-10.265) | -8.107(-17.938-3.339) |  | 48.688(33.652-68.672) | -0.256(-0.493-0.113) | 9.411(7.757-11.088) | -5.585(-14.126-4.470) |  | |
| Somalia | 0.388(0.231-0.562) | -0.351(-0.542--0.092) | 3.246(2.286-4.296) | -20.864(-36.542--0.916) |  | 10.447(6.040-15.287) | -0.341(-0.548--0.028) | 3.348(2.352-4.426) | -18.307(-36.039-4.261) |  | |
| South Africa | 0.322(0.242-0.397) | -0.534(-0.607--0.447) | 5.194(4.260-6.231) | -44.425(-50.891--36.706) |  | 8.997(6.923-11.059) | -0.527(-0.596--0.445) | 5.943(4.900-7.083) | -39.309(-45.102--32.513) |  | |
| South Korea | 1.777(1.302-2.397) | -0.818(-0.859--0.749) | 13.407(10.585-16.532) | -23.917(-35.573--9.479) |  | 34.857(26.255-46.052) | -0.853(-0.885--0.792) | 12.068(9.689-14.572) | -27.022(-37.358--14.620) |  | |
| South Sudan | 0.344(0.195-0.502) | -0.330(-0.554--0.034) | 3.603(2.635-4.720) | -23.760(-37.976--5.177) |  | 8.665(4.792-12.736) | -0.336(-0.572--0.005) | 3.594(2.615-4.764) | -24.183(-39.414--5.215) |  | |
| Spain | 0.662(0.523-0.827) | -0.745(-0.784--0.700) | 10.946(8.732-13.363) | -30.869(-39.797--21.715) |  | 15.326(12.305-18.642) | -0.746(-0.782--0.704) | 11.408(9.252-13.662) | -30.547(-38.593--22.160) |  | |
| Sri Lanka | 0.180(0.107-0.266) | -0.735(-0.833--0.613) | 5.007(3.993-6.077) | -34.943(-45.423--22.714) |  | 4.451(2.571-6.544) | -0.724(-0.832--0.596) | 5.035(4.008-6.050) | -32.761(-43.063--20.054) |  | |
| Sudan | 1.177(0.415-1.865) | -0.308(-0.560-0.050) | 7.672(4.940-10.464) | -5.101(-25.497-21.545) |  | 25.845(9.421-41.432) | -0.363(-0.601--0.015) | 6.953(4.531-9.434) | -7.192(-26.996-19.030) |  | |
| Suriname | 0.474(0.312-0.713) | -0.497(-0.658--0.308) | 6.704(5.386-8.217) | -19.990(-31.862--6.087) |  | 12.577(8.352-18.775) | -0.486(-0.644--0.283) | 7.209(5.871-8.762) | -19.358(-31.037--6.669) |  | |
| Swaziland | 0.273(0.176-0.375) | -0.408(-0.602--0.146) | 2.839(2.164-3.624) | -29.938(-43.373--12.715) |  | 6.861(4.431-9.576) | -0.394(-0.582--0.135) | 2.769(2.135-3.490) | -30.888(-43.200--16.110) |  | |
| Sweden | 0.348(0.258-0.445) | -0.705(-0.757--0.648) | 10.314(8.168-13.010) | -18.055(-28.830--5.887) |  | 7.038(5.381-8.969) | -0.721(-0.765--0.669) | 10.315(8.270-12.641) | -17.487(-26.881--7.133) |  | |
| Switzerland | 0.439(0.341-0.566) | -0.735(-0.779--0.675) | 11.769(9.431-14.146) | -24.713(-34.828--14.060) |  | 9.129(7.225-11.634) | -0.750(-0.791--0.698) | 11.441(9.394-13.711) | -26.214(-34.675--15.916) |  | |
| Syria | 0.577(0.387-0.796) | -0.408(-0.590--0.084) | 9.411(7.381-11.431) | -22.154(-34.634--4.384) |  | 12.296(8.271-17.230) | -0.463(-0.636--0.158) | 8.967(7.185-10.763) | -21.909(-32.343--6.572) |  | |
| Taiwan(China) | 1.031(0.795-1.304) | -0.672(-0.741--0.584) | 11.635(9.459-14.116) | -28.202(-39.340--14.841) |  | 20.815(16.264-26.053) | -0.709(-0.769--0.630) | 10.741(8.769-12.892) | -29.285(-39.585--17.518) |  | |
| Tajikistan | 1.187(0.794-1.758) | -0.708(-0.806--0.569) | 8.143(5.930-10.692) | -42.031(-56.164--24.521) |  | 28.604(19.204-42.416) | -0.735(-0.828--0.599) | 7.765(5.695-10.170) | -43.655(-57.427--25.729) |  | |
| Tanzania | 0.295(0.194-0.409) | -0.528(-0.657--0.353) | 4.778(3.800-5.942) | -27.281(-36.856--15.879) |  | 7.455(4.805-10.538) | -0.527(-0.662--0.347) | 4.941(3.960-6.099) | -24.990(-34.287--13.201) |  | |
| Thailand | 0.601(0.293-0.878) | -0.586(-0.718--0.396) | 8.207(6.215-9.881) | -35.192(-44.058--24.089) |  | 15.786(7.478-23.325) | -0.554(-0.700--0.344) | 7.948(6.023-9.589) | -33.886(-42.224--22.922) |  | |
| The Bahamas | 0.410(0.287-0.557) | -0.419(-0.580--0.204) | 4.660(3.565-6.079) | -9.721(-26.231-10.216) |  | 9.835(6.940-13.182) | -0.451(-0.601--0.249) | 4.603(3.585-5.769) | -10.425(-26.632-8.067) |  | |
| The Gambia | 0.113(0.073-0.154) | -0.481(-0.659--0.238) | 3.190(2.460-3.921) | -38.450(-48.596--26.047) |  | 3.004(1.948-4.120) | -0.493(-0.664--0.239) | 3.477(2.672-4.270) | -38.527(-48.766--26.958) |  | |
| Timor-Leste | 0.616(0.412-0.931) | -0.334(-0.544-0.024) | 7.953(6.091-10.150) | -7.739(-23.826-12.469) |  | 15.089(9.894-23.054) | -0.347(-0.556-0.025) | 8.094(6.209-10.272) | -4.052(-19.891-18.268) |  | |
| Togo | 0.443(0.272-0.640) | -0.359(-0.568--0.030) | 3.764(2.911-4.809) | -33.264(-48.609--15.689) |  | 11.107(6.727-16.062) | -0.335(-0.560-0.023) | 3.991(3.124-4.946) | -28.763(-44.323--10.763) |  | |
| Tokelau | 0.644(0.466-0.884) | -0.476(-0.611--0.279) | 5.867(4.577-7.406) | -15.095(-31.055-4.376) |  | 17.277(12.484-24.419) | -0.462(-0.608--0.250) | 6.385(5.044-7.891) | -13.637(-27.955-4.145) |  | |
| Tonga | 1.647(1.177-2.311) | -0.352(-0.527--0.121) | 9.832(7.853-12.416) | -19.644(-33.670--4.658) |  | 39.484(27.822-55.382) | -0.343(-0.534--0.098) | 9.771(7.881-11.976) | -17.622(-30.068--3.725) |  | |
| Trinidad and Tobago | 0.280(0.191-0.384) | -0.639(-0.731--0.511) | 5.392(4.317-6.513) | -19.077(-28.701--7.840) |  | 7.365(5.044-10.189) | -0.616(-0.718--0.478) | 5.770(4.670-6.808) | -20.856(-29.437--10.667) |  | |
| Tunisia | 0.547(0.352-0.774) | -0.442(-0.623--0.163) | 12.720(10.180-15.506) | -12.961(-28.009-9.159) |  | 11.670(7.302-16.371) | -0.438(-0.627--0.152) | 11.591(9.366-14.056) | -12.182(-27.672-8.232) |  | |
| Turkey | 1.400(0.978-1.933) | -0.654(-0.750--0.520) | 12.094(9.497-14.674) | -26.210(-37.822--11.243) |  | 33.060(22.675-45.918) | -0.676(-0.765--0.552) | 12.361(9.821-14.774) | -23.531(-35.026--10.227) |  | |
| Turkmenistan | 0.821(0.583-1.133) | -0.704(-0.785--0.599) | 8.565(6.815-10.394) | -32.327(-40.946--22.606) |  | 21.599(15.276-29.743) | -0.717(-0.794--0.614) | 8.291(6.629-10.026) | -35.850(-44.187--26.835) |  | |
| Tuvalu | 1.060(0.784-1.427) | -0.387(-0.548--0.181) | 7.043(5.637-8.682) | -7.072(-23.626-13.318) |  | 29.764(22.052-39.914) | -0.386(-0.554--0.161) | 7.814(6.253-9.676) | -4.147(-20.102-15.372) |  | |
| Uganda | 0.170(0.123-0.235) | -0.439(-0.591--0.212) | 2.680(2.189-3.274) | -18.983(-32.111--2.376) |  | 4.286(3.061-6.025) | -0.436(-0.601--0.184) | 2.740(2.248-3.364) | -18.034(-30.935--2.508) |  | |
| Ukraine | 0.990(0.638-1.438) | -0.697(-0.797--0.566) | 9.660(7.072-12.203) | -22.277(-36.780--5.549) |  | 28.394(18.021-41.408) | -0.699(-0.801--0.569) | 10.062(7.450-12.577) | -21.794(-35.627--7.010) |  | |
| United Arab Emirates | 0.427(0.290-0.615) | -0.603(-0.722--0.449) | 5.290(3.924-6.891) | -26.985(-44.286--3.904) |  | 7.907(5.496-11.205) | -0.680(-0.774--0.561) | 4.986(3.819-6.399) | -29.176(-44.175--8.062) |  | |
| United Kingdom | 0.508(0.392-0.638) | -0.764(-0.793--0.733) | 11.020(8.567-13.723) | -37.634(-45.029--29.840) |  | 10.013(7.918-12.198) | -0.780(-0.804--0.756) | 10.758(8.536-13.124) | -38.917(-45.205--31.945) |  | |
| United States | 0.348(0.273-0.430) | -0.623(-0.658--0.584) | 12.256(9.767-14.921) | -28.822(-34.761--21.985) |  | 8.093(6.515-9.847) | -0.635(-0.665--0.600) | 11.701(9.477-14.092) | -34.105(-39.242--27.886) |  | |
| Uruguay | 0.997(0.778-1.228) | -0.392(-0.475--0.298) | 9.291(7.471-11.152) | -6.034(-16.281-3.677) |  | 25.534(20.316-30.936) | -0.403(-0.481--0.315) | 10.284(8.376-12.172) | -8.920(-17.869--0.640) |  | |
| Uzbekistan | 0.436(0.318-0.573) | -0.502(-0.631--0.350) | 5.697(4.537-6.814) | 34.110(16.403-53.318) |  | 12.256(9.054-15.841) | -0.538(-0.653--0.400) | 5.912(4.765-7.057) | 28.061(11.262-45.726) |  | |
| Vanuatu | 0.813(0.570-1.130) | -0.431(-0.578--0.219) | 4.979(3.911-6.093) | -27.214(-36.906--17.554) |  | 22.185(15.406-31.498) | -0.426(-0.577--0.210) | 5.268(4.215-6.373) | -26.572(-35.182--17.227) |  | |
| Venezuela | 0.527(0.366-0.736) | -0.640(-0.732--0.521) | 4.827(3.856-5.891) | -33.685(-42.092--24.909) |  | 13.152(9.040-17.997) | -0.633(-0.732--0.508) | 4.939(4.014-5.993) | -35.126(-42.576--27.244) |  | |
| Vietnam | 0.817(0.592-1.184) | -0.536(-0.683--0.297) | 10.615(8.518-12.787) | -9.963(-21.386-7.849) |  | 21.416(15.359-29.918) | -0.529(-0.685--0.285) | 10.869(8.781-13.046) | -6.236(-18.057-11.689) |  | |
| Virgin Islands, U.S. | 0.286(0.170-0.431) | -0.617(-0.755--0.420) | 5.167(3.443-7.277) | -17.757(-39.171-11.627) |  | 6.597(4.048-9.666) | -0.623(-0.753--0.421) | 4.955(3.452-6.763) | -20.772(-40.613-5.974) |  | |
| Yemen | 2.221(0.811-3.287) | -0.218(-0.479-0.209) | 11.361(7.925-14.120) | -0.861(-17.645-20.686) |  | 51.037(18.686-75.409) | -0.299(-0.525-0.102) | 10.907(7.835-13.415) | -4.332(-19.445-14.126) |  | |
| Zambia | 0.275(0.180-0.380) | -0.399(-0.564--0.151) | 3.664(2.831-4.492) | -15.889(-30.961-1.491) |  | 6.694(4.377-9.292) | -0.397(-0.562--0.144) | 3.547(2.729-4.324) | -13.702(-29.427-3.199) |  | |
| Zimbabwe | 0.798(0.585-1.058) | -0.100(-0.354-0.270) | 5.807(4.478-7.363) | -22.005(-36.578--4.045) |  | 20.165(14.501-26.980) | -0.034(-0.331-0.345) | 5.799(4.566-7.454) | -21.981(-36.070--4.895) |  | |

Note: DALY=disability adjusted life-years; PAF=population attributable fraction
